# The innate immune receptor NLRX1 is a novel required modulator for mPTP opening: implications for cardioprotection

**DOI:** 10.1007/s00395-025-01124-x

**Published:** 2025-06-19

**Authors:** Y. Xiao, X. Hu, C. F. Rudolphi, E. E. Nollet, R. Nederlof, Q. Wang, D. Bakker, Panagiota Efstathia Nikolaou, J. C. Knol, R. R. Goeij-de Haas, A. A. Henneman, T. V. Pham, C. R. Jimenez, A. E. Grootemaat, N. N. van der Wel, S. E. Girardin, N. Kaludercic, J. van der Velden, Z. Onódi, P. Leszek, Z. V. Varga, P. Ferdinandy, B. Preckel, N. C. Weber, M. W. Hollmann, F. Di Lisa, C. J. Zuurbier

**Affiliations:** 1 Laboratory of Experimental Intensive Care and Anesthesiology, Department of Anesthesiology, Amsterdam UMC, University of Amsterdam, Meibergdreef 9, 1105 AZ Amsterdam, The Netherlands; 2 Amsterdam Cardiovascular Sciences, Atherosclerosis & Ischemic Syndromes, Heart Failure & Arrhythmias, Amsterdam, The Netherlands; 3 Department of Cardiology, Renmin Hospital of Wuhan University, Wuhan, 430060 People’s Republic of China; 4 Department of Physiology, Amsterdam UMC, Vrije Universiteit Amsterdam, Amsterdam, The Netherlands; 5 Institut Für Herz- Und Kreislaufphysiologie, Medizinische Fakultät Und Universitätsklinikum Düsseldorf, Heinrich-Heine-Universität Düsseldorf, Düsseldorf, Germany; 6 Laboratory of Pharmacology, Faculty of Pharmacy, National and Kapodistrian University of Athens, Athens, Greece; 7 Proteomics Core Resource, Amsterdam UMC, Location VUmc, Amsterdam, The Netherlands; 8 Department of Medical Oncology, Vrije Universiteit Amsterdam, Amsterdam UMC, Location VUmc, Amsterdam, The Netherlands; 9 Electron Microscopy Centre Amsterdam, Medical Biology, Amsterdam University Medical Centre (UMC), Amsterdam, The Netherlands; 10 Department of Laboratory Medicine and Pathobiology, University of Toronto, Toronto, ON Canada; 11 Neuroscience Institute, National Research Council of Italy, Via U. Bassi 58/B, 35121 Padua, Italy; 12 Department of Biomedical Sciences, University of Padova, Via U. Bassi 58/B, 35121 Padua, Italy; 13 Fondazione Istituto Di Ricerca Pediatrica Città Della Speranza, Corso Stati Uniti 4, 35127 Padua, Italy; 14 HCEMM-SE Cardiometabolic Immunology Research Group, Department of Pharmacology and Pharmacotherapy, Semmelweis University, Budapest, Hungary; 15 MTA-SE Momentum Cardio-Oncology and Cardioimmunology Research Group, Semmelweis University, Budapest, Hungary; 16 Department of Heart Failure and Transplantology, Cardinal Stefan Wyszyński National Institute of Cardiology, 04-628 Warsaw, Poland; 17 Pharmahungary Group, Szeged, Hungary

**Keywords:** Mitochondrial transition pore opening, I/R injury, Mitochondria, RISK pathway, NLRX1, AMPK

## Abstract

NLRX1 is the only NOD-like innate immune receptor that localises to mitochondria. We previously demonstrated that NLRX1 deletion increased infarct size in isolated mouse hearts subjected to ischemia–reperfusion injury (IRI); however, underlying mechanisms are yet to be identified. Given the crucial role played by mitochondria in cardiac IRI, we here hypothesise that NLRX1 affects key mechanisms of cardiac IRI. Cardiac IRI was evaluated in isolated C57BL/6J (WT) and NLRX1 knock out (KO) mouse hearts. The following known modulators of IRI were explored in isolated hearts, isolated mitochondria; or permeabilised cardiac fibres: 1) mTOR/RISK/autophagy regulation, 2) AMPK and mitochondrial energy production, and 3) mitochondrial permeability transition pore (mPTP) opening. NLRX1 deletion increased IRI, and cardiac NLRX1 was decreased after IRI in mouse and pig hearts. NLRX1 ablation caused decreased mTOR and RISK pathway (Akt, ERK, and S6K) activation following IR, without affecting autophagy/inflammation/oxidative stress markers. The RISK activator Urocortin dissipated NLRX1 effects on mTOR, RISK pathway and IRI, indicating that increased cardiac IRI with NLRX1 deletion is, at least partly, due to impaired RISK activation. The energy sensor AMPK was activated in NLRX1 KO hearts, possibly due to slowed mitochondrial respiratory responses (impaired mitochondrial permeability) towards palmitoylcarnitine in permeabilised cardiac fibres. NLRX1 deletion completely abolished calcium-induced mPTP opening, and cyclosporine A (CsA) effects on mPTP, both before and after IR, and was associated with increased mitochondrial calcium content after IR. Mitochondrial sub-fractionation studies localised NLRX1 to the inner mitochondrial membrane. NLRX1 deletion associated with decreased phosphorylation of mitochondrial Got2, Cx43, Myl2, Ndufb7 and MICOS10. The mPTP inhibitor CsA abolished IRI differences between KO and WT hearts, suggesting that the permanent closure of mPTP due to NLRX1 deletion contributed to the increased IR sensitivity of NLRX1 KO hearts. This is the first demonstration that the mitochondrial NLRX1 is a novel factor required for mPTP opening and contributes to cardioprotection against acute IRI through RISK pathway activation and prevention of permanent mPTP closure.

**Supplementary Information:**

The online version contains supplementary material available at 10.1007/s00395-025-01124-x.

## Introduction

The innate immune system plays an important role in cardiac ischemia–reperfusion injury (IRI) [88]. The pro-inflammatory toll-like receptors (TLR2 [3, 4], TLR3 [44], TLR4 [53]) and the not-constitutively expressed NACHT, LRR, and PYD domains-containing protein 3 (NLRP3) inflammasome [46, 60, 74] aggravate cardiac IRI through pro-inflammatory cytokines and pyroptosis. In contrast, the nucleotide-binding oligomerisation domain (NOD)-like receptor X1 (NLRX1) is considered to be anti-inflammatory, is constitutively expressed in most tissues including the heart, and contains a mitochondrial-targeting domain [14, 71]. We have shown previously that deletion of NLRX1 aggravated cardiac IRI in the isolated mouse heart for both cardiac functional parameters and cell death (infarct size) and was associated with decreased Akt signalling at early reperfusion and activated glucose metabolism at baseline [83]. However, whether these changes were causally related to increased IRI, and whether NLRX1 exerted IR effects through other well-known mechanisms of cardiac IRI remained unexplored [83]. Aside from the well-known anti-inflammatory function, NLRX1 has been reported to regulate autophagy, a typical housekeeping process, in various pathological conditions in tissues [28, 38, 39, 80] other than heart. NLRX1 has been found to be a novel mitophagy receptor which directly associates with microtubule-associated protein 1-light chain 3, LC3, a central protein in the autophagy pathway [86]. NLRX1 also regulates mitophagy via FUNDC1-NIPSNAP1/NIPSNAP2 signalling pathway in intestinal IRI [42]. Although autophagy is involved in cardiac IRI [79], it is unknown whether the autophagic machinery during the early phase of acute IRI is also modulated by NLRX1 in the heart. The RISK/mTOR pathway is tightly associated with changes in autophagy and is another major regulator of cardiac IRI, activation of which provides protection against cell death [64, 66, 87]. The RISK pathway has been identified as a key component in ischemic preconditioning [11, 56], ischemic postconditioning [20], and remote ischemic preconditioning [69] cardioprotective interventions. In our previous work [83], we observed diminished p-Akt at early reperfusion in the NLRX1 KO hearts, suggestive of NLRX1 affecting the RISK pathway. Thus, the first goal of the present study was to examine NLRX1 effects on autophagy, RISK/mTOR pathways and inflammation during IRI in the heart.

NLRX1 has also been found to regulate energy metabolism and mitochondrial activity, factors that can also modulate cardiac IRI. Recent studies demonstrated that NLRX1 regulated the mitochondrial RNA processing of complex I and complex IV [68]. Deletion of NLRX1 increased mitochondrial fatty acid-dependent oxidative phosphorylation (OXPHOS), decreased glycolysis in hepatocytes and protected against diet-induced metabolic syndrome and non-alcoholic fatty liver disease (NAFLD) development [35]. In contrast, glucose oxidation in the heart was increased with NLRX1 deletion [83], demonstrating NLRX1 effects on metabolism to be organ specific. In addition, NLRX1 protected against mitochondrial damage and epithelial cell apoptosis during renal IRI by decreasing OXPHOS and oxidative stress [72]. Notably NLRX1-dependent renal protection could be abolished by the mPTP inhibitor cyclosporine A, suggesting that NLRX1 effects were possibly mediated through mPTP regulation. However, the mPTP was not further examined in this kidney IRI study [72], whereas mPTP figures prominently in cardiac IRI. Thus, the second goal of the study was to examine NLRX1 effects on mitochondrial-associated factors with known effects on cardiac IRI [26, 61, 76], such as the energy sensor AMPK [47], mitochondrial substrate oxidation [89], mPTP regulation [7, 70], and mitochondrial ROS production [16, 27].

Employing Langendorff-perfused mouse heart IR models, permeabilised cardiac fibres, and isolated mitochondria, the present study indicates that NLRX1 exerts its effects on cardiac IRI through regulation of the RISK pathway and mPTP opening, whereby NLRX1 emerges as a novel important regulator of cardiac mitochondrial transition pore opening, mandatory to prevent permanent closure of the mPTP.

## Methods

Detailed methods are available in Supplementary material. All uncut Western blots can be found in Supplementary file 2.

### Animals

NLRX1^−/−^ mice on a C57BL/6J background were generated as reported [83] and bred at our Animal Research Institute AMC (ARIA) institute, Amsterdam UMC, University of Amsterdam. Male NLRX1^−/−^ mice and age (11–28 weeks) and weight matched C57BL/6J wild-type (WT) mice (Janvier Labs, France) were used. All mice were housed in standard housing conditions and had food and water ad libitum. All mice experimental protocols were registered and approved by the Animal Ethics Committee of the Academic Medical Center, Amsterdam, The Netherlands, and conducted pursuant to the Guide for the Use and Care of Laboratory Animals.

The investigation of pig heart tissues conforms to the Guide for the Care and Use of Laboratory Animals published by the US National Institutes of Health (NIH publication No. 85–23, revised 1996), to the EU Directive (2010/63/EU) and was approved by the animal ethics committee of Hungarian National Food Chain Safety Office (SOI/31/26–11/2014).

### Ex vivo mouse cardiac IR model

Induction of myocardial IR injury was conducted as previously reported [78, 83]. Briefly, all mice were anesthetized and heparinized with an intraperitoneal injection of sodium pentobarbital (95 mg/kg body weight) and heparin (15 IU). After confirmation of proper anesthesia with pedal withdrawal reflexes, mice were intratracheally ventilated, then hearts were quickly perfused by aorta cannulation in chest. Subsequently, excised hearts were connected to Langendorff setup and perfused under a constant flow at initial perfusion pressure of 80 mmHg at 37 °C with Krebs–Henseleit (KH) buffer [ (in mmol/L), 118 NaCl, 4.7 KCl, 1.2 MgSO_4_, 1.2 KH_2_PO_4_, 25 NaHCO_3_, 0.5 EDTA, 2.50 CaCl_2_, 5.5 D-glucose, 0.5 L-glutamine, 1 lactate, 0.1 pyruvate, 1% (g/L) albumin – 0.2mM palmitic acid sodium salt, 0.05 L-carnitine, and 30 mU/L insulin]. Cardiac function was continuously monitored throughout each protocol via a water-filled polyethylene balloon inserted into left ventricular cavity.

### Experimental protocols and hypotheses tested

**Fig. 1:**
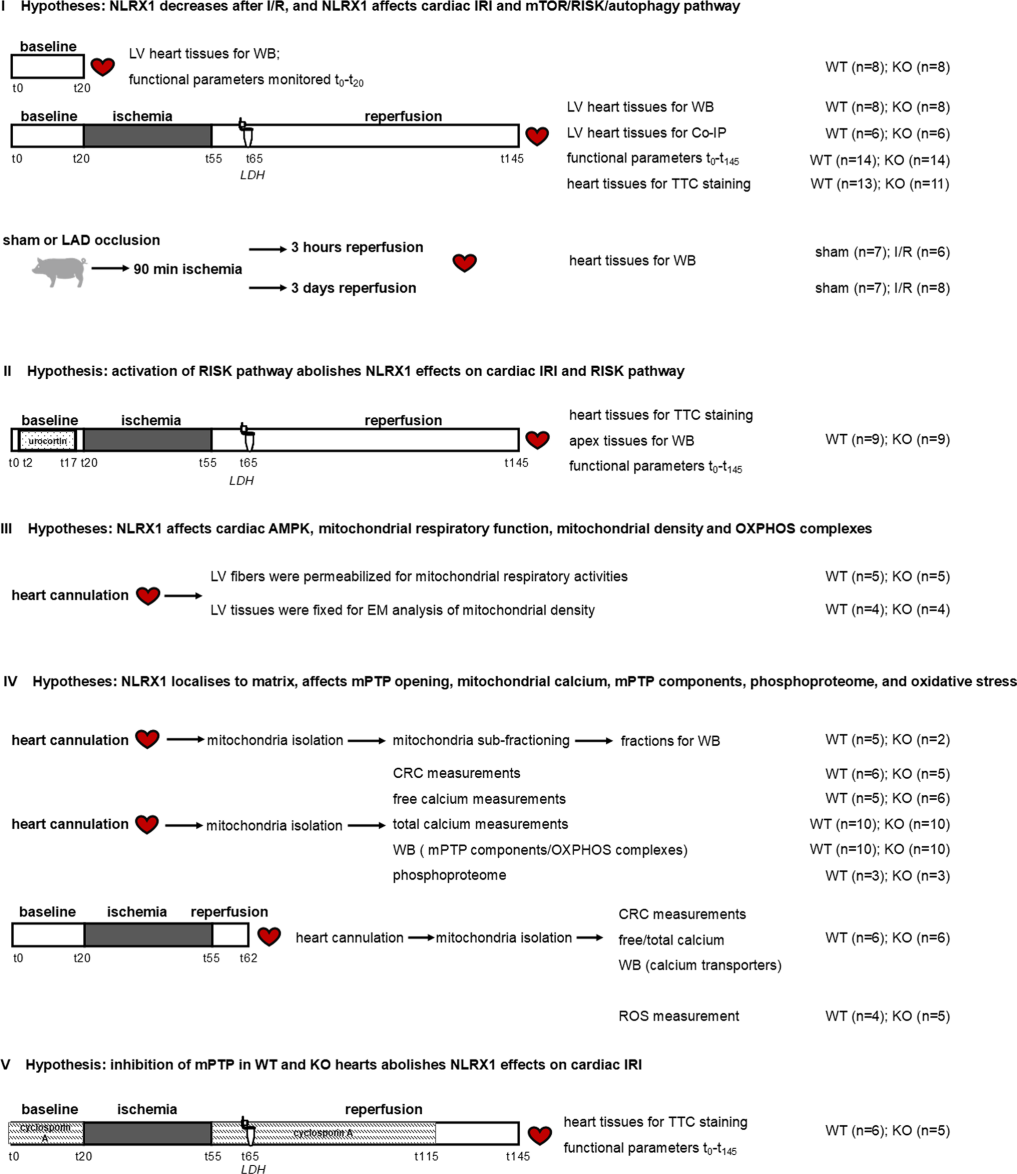
Experimental series and hypotheses being tested (I) *NLRX1 and cardiac IRI and mTOR/RISK/autophagy pathways*: Cardiac NLRX1 levels were determined in post-ischemic mouse and pig hearts, WT and NLRX1^−/−^ isolated mouse hearts were subjected to 20 min baseline normoxic perfusion or to 20 min baseline perfusion followed by 35 min global ischemia (I) and 90 min reperfusion (R) and cardiac IR injury and signalling pathways evaluated; (II) *NLRX1 and the role of RISK pathway activation in cardiac IRI*: WT and NLRX1^−/−^ hearts were pretreated with 2 nM urocortin during 15min baseline, followed by I/R as series I; (III) *NLRX1 and cardiac energy regulation*: cardiac AMPK was determined from experimental series I, cardiac fibres permeabilised for mitochondrial respiratory measurements in Oxygraph-2k, hearts fixated for EM analysis of mitochondrial density, and OXPHOS components determined in isolated mitochondria from series IV; (IV) *NLRX1 and mPTP regulation*: Mitochondria were isolated from WT and KO hearts for determination of NLRX1 localisation and calcium retention capacity (CRC) to determine Ca^2+^-sensitivity of mPTP opening, mitochondrial calcium and mPTP components and phosphoproteome analysis for hearts immediately sacrificed or subjected to 20 min baseline perfusion followed by 35 min I and 7 R, and mitochondrial ROS production at early reperfusion; (V) *NLRX1 and the role of mPTP inhibition in cardiac IRI*: WT and NLRX1^−/−^ hearts were treated with 500 nM cyclosporine A during 20min baseline and the first 60min of reperfusion, I/R procedure as series I

In the first experimental series I (I; Fig. 1), the primary hypotheses being tested were whether NLRX1 decreased after IR, and whether NLRX1 ablation affected the mTOR/RISK and autophagy pathways before and/or after IR. Additionally, we used this series also to confirm our previous work [83] that NLRX1 ablation increased IRI, employing three of the four IR outcome parameters [end-diastolic pressure (EDP), % rate-pressure recovery (%RPP) and LDH release]. Infarct size could not be determined in this series because heart tissue was needed for extended molecular analysis of the different protective pathways. After 20 min equilibration, the hearts from WT and NLRX1^−/−^ mice were subjected either to 20 min baseline normoxic perfusion or to baseline perfusion followed by 35 min global ischemia (I) and 90 min reperfusion (R). However, because quantification of infarct size is the gold standard for cardiac IRI evaluation [9], infarct size was determined in an additional series of experiments. Effects of IR on cardiac NLRX1 were also examined in pig studies subjected to 90 min ischemia and 3 h or 3 days of reperfusion.

In experimental series II (II, Fig. 1), the hypothesis was tested whether the observed changes in the RISK pathway from series #1 may explain, at least partly, NLRX1 effects on cardiac IRI. To this end, the RISK pathway was activated in both WT and KO hearts by treatment with 2 nM urocortin during the last 15 min baseline perfusion to dissipate the genotype differences in RISK pathway activation. Hearts were subjected to the IRI protocol from series I, and IRI between genotypes compared.

In experimental series III (III, Fig. 1), the hypothesis was tested that NLRX1 affects whole heart cardiac energy status and the underlying energy production by mitochondria in permeabilised cardiac fibres, with focus on substrate dependency of mitochondrial maximal respiration and responsiveness of mitochondria to substrates. Cardiac fibres from WT and KO were permeabilised and mitochondrial respiration determined using an Oxygraph-2K apparatus (see below for further description).

Then, in experimental series IV (IV, Fig. 1), because of the observed effects of NLRX1 on mitochondrial respiration and slowed responsiveness in series III, NLRX1 effects on mitochondria were further characterized. First, NLRX1 submitochondrial location and a possible role for NLRX1 in mPTP regulation through the use of a calcium retention capacity assay was determined. Additionally, NLRX1 effects on 1) total and free mitochondrial calcium before and after I/R, 2) known mPTP components, 3) Ca^2+^-transporters, 4) phosphoproteome of mitochondrial proteins, and 5) ROS production of mitochondria isolated at 7 min reperfusion were also examined.

Finally, in experimental series V (V, Fig. 1), the observation from experimental series IV, that mPTP opening was abolished in NLRX1 deleted mitochondria, was tested as explanation for increased IRI in NLRX1 KO hearts. To this end, both WT and KO hearts were treated with an mPTP inhibitor to test whether pharmacological abolishment of mPTP opening dissipated the cardiac IRI differences between genotypes. Hearts were subjected to a similar I/R procedure as in series II, with administration of 500 nM cyclosporine A during the last 20 min baseline and the first 60min of reperfusion.

### Porcine infarction model and tissue collection

To examine whether cardiac IR also decreases NLRX1 in larger animals than mice, we examined cardiac NLRX1 in sham Landrace pigs and cardiac IR Landrace pigs (90 min ischemia followed by either 3 h (*n* = 7 sham, *n* = 6 I/R) or 3 days (*n* = 7 sham, *n* = 8 I/R) recovery employing a porcine myocardial IR model reported previously [10]. Heart tissues were collected from ischemic and non-ischemic cardiac regions.

### Lactate dehydrogenase (LDH) measurement

Lactate dehydrogenase (LDH) release was monitored in the coronary effluent by spectrophotometry, and LDH activity as index of cell death was blindly determined. Effluent was collected at 10 min reperfusion (see Fig. 1), knowing that at this time point LDH release is maximal and correlated well with infarct size in the isolated mouse heart[54, 83].

### Infarct size determination

After 90 min reperfusion heart was stored at − 20 °C. 2, 3, 5-triphenyltetrazolium chloride (TTC, Sigma–Aldrich, St. Louis, MO, USA) staining was performed within 1 week. Infarct size was quantified relative to ventricular area (excluding atria) and analysed with SigmaScan Pro5 software by an investigator not aware of treatment allocation.

### Measurement of mitochondrial respiratory activities

Mitochondrial function was assessed using respirometry by an investigator not aware of genotype allocation. Hearts were excised following aorta cannulation and perfused with high potassium KH buffer. The left ventricle free wall was quickly cut and placed in ice cold BIOPS buffer. Fresh left ventricular fiber bundles (≈2 mg) were gently separated using ultra-thin forceps and permeabilised in ice-cold BIOPS solution containing 50 µg/mL saponin for 25 min. Next, fiber bundles were washed twice for 10 min in ice-cold mitochondrial respiration medium, rapidly blotted dry, weighed, and inserted into a high-resolution respirometer (Oxygraph-2k; Oroboros Instruments). All experimental protocols were performed *in duplo* at 37 °C under oxygen levels above 300 µM throughout the experiment to avoid oxygen supply limitations.

### Electron microscopy

Electron microscopy was performed to evaluate mitochondria density and structure. Briefly, LV tissues were cut into longitudinal strips and fixed for 4 h at room temperature. Then samples were transferred to storage buffer in 4 °C. After washing, tissue was embedded and sectioned for imaging. Mitochondria number was quantified by intersection points counting (points hitting the mitochondrion. Twenty different images were quantified, and the average was calculated for each heart.

### Calcium retention capacity

We measured calcium retention capacity (CRC) in the isolated mitochondria which could provide important information on the resistance of the mitochondria to Ca^2+^-induced mPTP opening. 0.25 mg/ml mitochondria suspension was prepared, then 0.5 µM membrane impermeable Calcium Green-5N probe (C3737, Invitrogen™) was added. 5 μM CaCl_2_ pulses were applied every 4 min and fluorescence signal was determined with Ex/Em = 485/532 nm at 25 °C. The CRC was defined as the total amount of CaCl_2_ required to trigger the massive increase of fluorescence signal or no further decrease of signal and was expressed as nmol per mg of mitochondrial protein.

### Mitochondrial ROS production

Mitochondria were isolated from WT (*N* = 4) or NLRX1 KO hearts (*n* = 5) excised after 35 min ischemia and 7 min reperfusion. Mitochondria isolation for H_2_O_2_ measurement was performed as described [48], and mitochondrial protein level was determined by BCA. Mitochondrial H_2_O_2_ production was measured with Amplex Red (5 µM, A12222, Thermo fisher) horseradish peroxidase (4 µg/ml) fluorescence method. Succinate (5 mM), without or with rotenone (2 μM), was added to the mitochondria. Fluorescence signal was determined with Ex/Em = 540/590 nm (at 37 °C) over a 30 min period. H_2_O_2_ production was calculated from a H_2_O_2_ standard curve and was normalized to mitochondrial protein (pmol H_2_0_2_/min/mg).

### Statistical analysis

Results are expressed as Mean ± SD or Median ± IQ (violin plot), n is the sample size, representing the number of biological replicates (equals number of animals). Shapiro–Wilk test was used to test the normality distribution of data. Comparisons of two groups: Student *t* test was performed when data were normally distributed. Non-normally distributed data were analysed with independent-samples Mann–Whitney U test. Comparisons with more than two groups were evaluated by one-way ANOVA followed by Games–Howell multiple comparisons test. Calcium retention capacity data and NADH curve were analysed by two-way repeated measures ANOVA with Bonferroni adjustment for multiple comparisons. Statistics were conducted using IBM SPSS statistics version 26 (International Business Machines Corp., Armond, NY, USA). Figures were made in GraphPad Prism 8.0 (GraphPad Software, Inc., La Jolla, CA, USA). In all tests, significance was accepted for *P* < 0.05.

## Results

### NLRX1 decreases in post-ischemic mouse and pig hearts and NLRX1 protects against IRI and facilitates mTOR and RISK pathway activation after ischemia

**Fig. 2:**
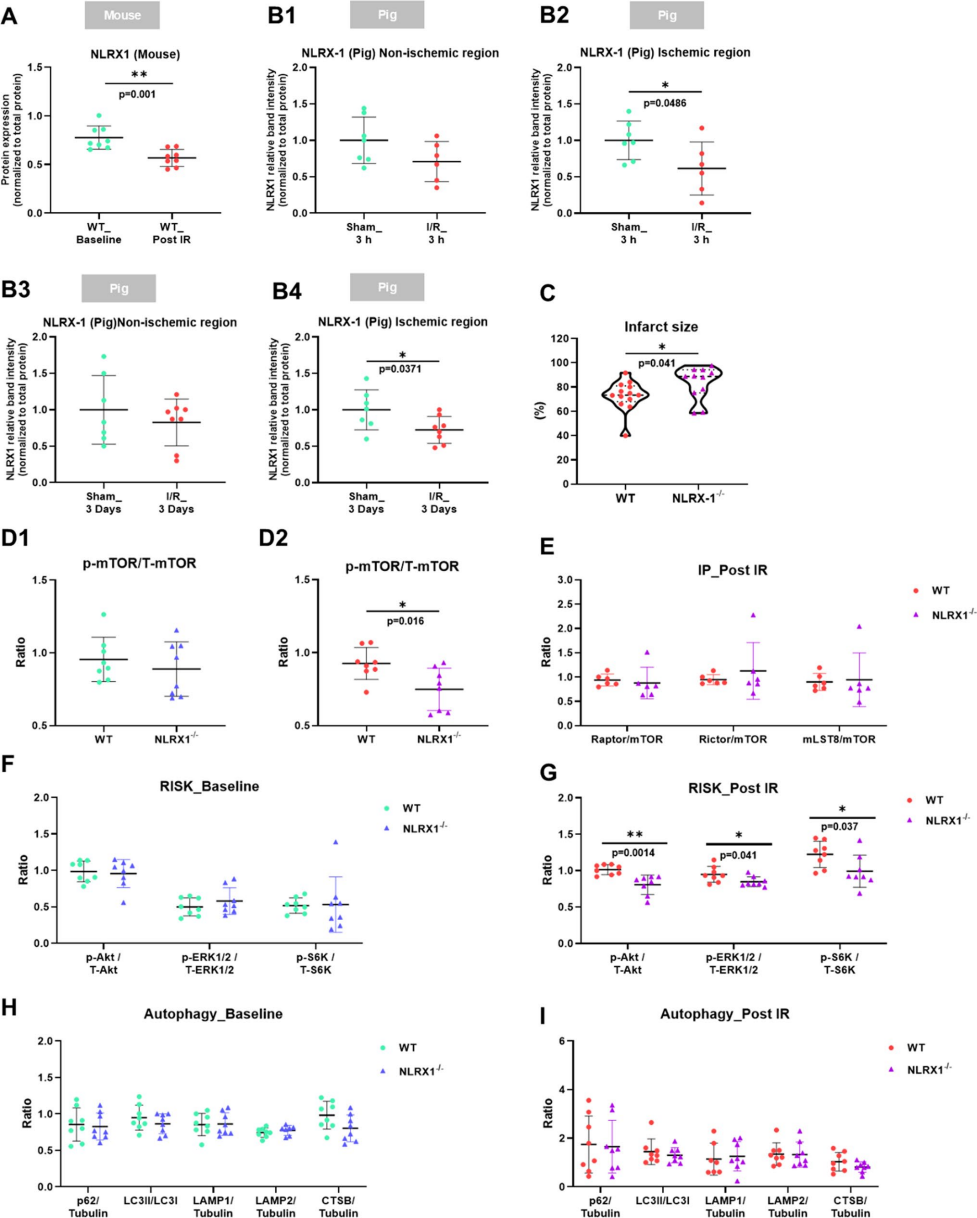
NLRX1 protects against cardiac IRI and is associated with mTOR and RISK pathway activation. Hearts from WT and NLRX1^−/−^ mice were subjected to 20 min normoxic perfusion (Baseline) followed by 35 min ischemia and 90 min reperfusion (Post IR). There was less protein expression of NLRX1 in WT mouse heart in post IR group than in baseline group (**A**, *n* = 8 per group), similar findings were observed after 3 h (*n* = 7 sham, n = 6 IR) and 3 d (*n* = 7 sham, *n* = 8 IR) reperfusion for NLRX1 in the ischemic regions of post IR pig hearts. (**B1-B4**). Data shown are mean ± SD (**A**-**B**).More severe IRI in NLRX1^−/−^ than in WT evident from increased infarct size (**C**, *n* = 13 WT, *n* = 11 KO). Data shown are median ± IQ (**C**). **D1** and **D2**, Representative expression analysis of phospho-mTOR/total mTOR at baseline and after IR in heart lysate (*n* = 8 per group). **E**, Co-immunoprecipitation (IP) was performed with hearts tissues from WT and NLRX1^−/−^ mice after IR procedure (*n* = 6 per group). mTOR complexes proteins (Raptor, Rictor, mTOR, mLST8) and NLRX1 protein levels were shown in. **F** and **G**, Reperfusion injury salvage kinase (RISK) survival signalling pathway at baseline and post IR were investigated (*n* = 8 per group). Expression analysis of phospho-Akt/total Akt, phospho-ERK/total ERK, and phospho-S6K/total S6K at baseline (**F**) and after IR (**G**) (*n* = 8 per group). Expression analysis of autophagic parameters (p62, LC3, LAMP1, LAMP2, CTSB) at baseline **(H)** condition or after IRI **(I)** (*n* = 8 per group). Data shown are mean ± SD (**D**-**I**). Statistical significance was evaluated by nonpaired t test (normally distributed data) or Mann–Whitney test (non-normally distributed data). *P < 0.05, **P < 0.01, ***P < 0.001, and ****P < 0.0001 between the groups indicated by the solid lines

The protein level of NLRX1 was decreased after IR in isolated WT mouse hearts, as well as in pig hearts at 3 h or 3 days of reperfusion (Fig. 2A, B1-B4), . NLRX1 KO was confirmed by the absence of NLRX1 in heart (Figure S1A). There were no differences in body weight, heart weight or cardiac function of isolated hearts between WT and NLRX1^−/−^ mice (Table S1). Furthermore, NLRX1 deletion was without effect on maintenance of cardiac performance during 20 min baseline perfusion (Figure S1B through G). When intact hearts were subjected to IR, the absence of NLRX1 significantly increased IR injury, as reflected by decreased cardiac function (increased end-diastolic pressure (EDP), reduced rate-pressure-product (RPP) recovery), and increased cell death (LDH release) in NLRX1^−/−^ compared to WT hearts, Figure S1H-J, respectively. In an additional experimental series, where the whole heart was used for determination of infarct size, NLRX1 deletion was also associated with an increased infarct size (Fig. 2C).

Then we explored whether NLRX1 effects on cardiac IRI were related to changes in mTOR/Akt pathway. We did observe impaired mTOR activity in NLRX1^−/−^ hearts after IR, but not at baseline (Fig. 2D1-D2). Co-immunoprecipitation (IP) for mTOR using heart tissue lysate further indicated that NLRX1 did not specifically affected mTORC1 or mTORC2, (Fig. 2E). The effect of NLRX1 on mTOR is not through direct interaction, because NLRX1 protein was not detected in IP samples. At baseline, NLRX1 deletion was without effect on the RISK pathway (Fig. 2F). Following IR, however, NLRX1 deletion caused decreased activation of the major components of the RISK pathway: decreased p-Akt, p-ERK1/2, and p-S6K (Fig. 2G). These data suggest that NLRX1 is needed to maintain optimal activation of the mTOR/RISK pathway after a stress-insult such as IR.

Because mTOR is a major regulator of autophagy, and NLRX1 is suggested to affect autophagy in different experimental models, we evaluated whether NLRX1 effects on cardiac IRI were related to changes in autophagy. However, no effect on autophagic parameters (p62, LC3, LAMP1, LAMP2, CTSB) at baseline condition or after IRI were observed (Fig. 2H-I). In addition, NLRX1 is reported to mitigate inflammation and oxidative stress in other tissues than the heart and may protect through these mechanisms against acute cardiac IRI. Therefore, inflammatory cytokines and protein level of 4-hydroxynonenal (4-HNE) and 3-nitrotyrosine (3-NT), two important biomarkers of oxidative/nitrative stress, were evaluated. However, although cytokines did increase in post IR hearts relative to the baseline condition, no differences in expression of IL-1β, IL-6 or TNFα level between WT and NLRX1^−/−^ hearts at baseline nor after IR were observed (Figure S2A through C). Furthermore, although IR increased myocardial 4-HNE and 3-NT level, confirming IR-induced oxidative stress in our IR model (S2D and S2E), NLRX1 deletion was without effect on these oxidative/nitrative stress markers at baseline and after IR (Figure S2F-I). Thus, changes in autophagy, inflammation or oxidative/nitrative stress cannot explain NLRX1 effects on the early phase of acute cardiac IRI.

### NLRX1 effects on IR injury is dependent on cardioprotective RISK signalling axis

**Fig. 3:**
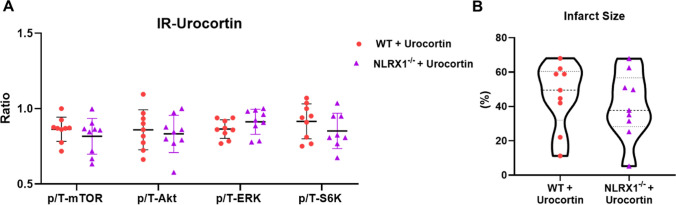
RISK activator urocortin negates genotype effects on post IR RISK pathway activation and thereby abrogates genotype effects on IRI. Each heart was perfused with 2 nM urocortin during baseline, then subjected to IR procedure (*n* = 9 per group). **A**, Representative protein expression analysis of phospho-mTOR /total mTOR, phospho-Akt/total Akt, phospho-ERK/total ERK, and phospho-S6K/total S6K (data shown are mean ± SD). **B**, Infarct size (% of area at risk) of 2, 3, 5-triphenyltetrazolium chloride (TTC) staining in the hearts after 90 min reperfusion. Data shown are median ± IQ (**B**). Statistical significance was evaluated by nonpaired t test (normally distributed data) or Mann–Whitney test (non-normally distributed data)

The diminished RISK pathway activation may contribute to the detrimental effects of NLRX1 deletion on IR injury. To test for a cause-effect relationship, we compared IRI for WT and KO hearts following pretreatment with the RISK activator urocortin before ischemia. There were no differences in cardiac functional parameters between urocortin perfused NLRX1^−/−^ and WT hearts at T0 (Table S1) or after 20 min of normoxic perfusion (Figure S3A through D). Urocortin was able to dissipate the genotype effects on post IR mTOR, Akt, ERK1/2 or S6K phosphorylation (Fig. 3A). In this condition of similar RISK pathway activation, the genotype effects on IRI were also dissipated: both functional recovery (Figure S3E and F) and cell death parameters (Fig. 3B and S3G) were now similar between the two genotypes. These data, therefore, indicate that attenuated RISK pathway activation, at least partly, contributes to the increased cardiac IRI in NLRX1 KO hearts.

### NLRX1 deletion increase cardiac energy stress and alters mitochondrial respiration regulation

**Fig. 4:**
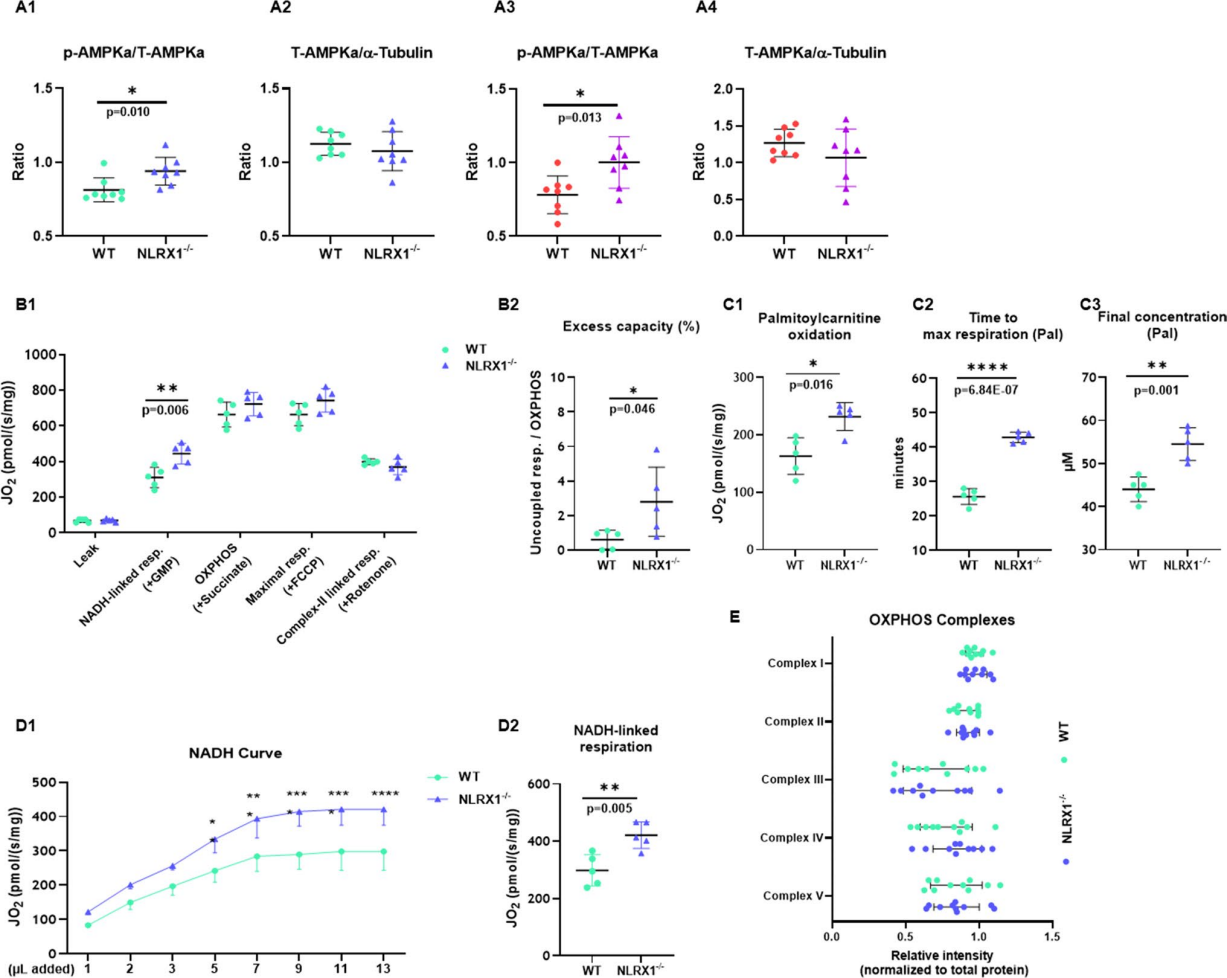
NLRX1 deletion increased capacity of complex 1 and delayed respiratory responses to FA carnitines. A1 and A2, Hearts from WT and NLRX1^−/−^ mice were subjected to 20min normoxic perfusion (Baseline) followed by 35min ischemia and 90 min reperfusion (Post IR). Representative immunoblots and analysis of phospho-AMPKα/total AMPKα at baseline (**A1, A2**) and after IR (**A3, A4**) in heart lysate (*n* = 8 per group). **B1-D2**, Mitochondrial respiration was detected in isolated heart fibers (*n* = 5 per group): **B1**, Rates of mitochondrial oxygen consumption during substrates-uncoupler-inhibitor Titration (SUIT) protocol. **B2**, Excess capacity calculated as the ratio of uncoupled respiration to OXPHOS. **C1**, Rates of mitochondrial oxygen consumption during palmitoylcarnitine oxidation. **C2**, The timing to reach maximal palmitoylcarnitine oxidation. **C3**, The final concentration to reach plateau of palmitoylcarnitine oxidation. **D1**, Rates of mitochondrial oxygen consumption during NADH saturate gradually. **D2**, Rates of mitochondrial oxygen consumption upon NADH saturation. **E**, Immunoblots’ analysis of OXPHOS complex I, II, III, IV, and V measured in isolated mitochondria lysate (*n* = 10 per group). Data shown are mean ± SD. Statistical significance was evaluated by nonpaired t test (normally distributed data) or Mann–Whitney test (non-normally distributed data). NADH curve (C1) was analysed by two-way repeated measures ANOVA with Bonferroni adjustment for multiple comparisons. *P < 0.05, **P < 0.01, ***P < 0.001, and ****P < 0.0001 between WT and NLRX1^−/−^ group

We then focused whether NLRX1 affected cardiac energy status and mitochondrial function, because these factors also play an important role in cardiac IR and previous literature by our group had shown that NLRX1 affected cardiac glucose metabolism. Therefore, we first explored whether NLRX1 affected the energy status of the heart by examining the energy sensor p-AMPK. Additionally, p-AMPK changes may also explain whether the impaired mTOR/Akt activation following IR is a results of increased p-AMPK signalling in the KO hearts, knowing that AMPK activation inhibits activation of mTOR/Akt during stress conditions [85]. Indeed, increased phosphorylation of the energy sensor AMPKα was observed in NLRX1 deleted hearts, both at baseline and after IRI (Fig. 4A1–A4), indicative that the ablation of NLRX1 caused an energy-deprivation status within the heart. To explore possible mitochondrial causes of this energy-deprivation condition with NLRX1 deletion, mitochondrial function was determined in isolated permeabilised cardiac fibres employing Oroboros high-resolution respirometry. The results showed that NLRX1^−/−^ heart tissues had higher complex I related respiration, without changes in leak, (CI + CII) OXPHOS, uncoupled or CII respiration (Fig. 4B1). When normalizing the uncoupled respiration (maximal respiration (+ FCCP)—OXPHOS) to OXPHOS respiration (the excess-capacity of the electron transferring complexes), NLRX1 ablation caused this excess-capacity to be increased (Fig. 4B2). However, because OXPHOS is not decreased in NLRX1^−/−^ heart fibers, the increase in excess capacity does not reflect mitochondrial inefficiency of ATP production but probably reflects hyperactivity of the electron transport chain (ETC) with NLRX1 deletion. NLRX1^−/−^ heart fibers also displayed higher palmitoylcarnitine oxidation (Fig. 4C1). Remarkably, NLRX1-deleted mitochondria needed much more time to reach maximal respiration (Fig. 4C2 and Suppl. Figure S4A), indicating that NLRX1 ablation impaired mitochondrial respiratory responses to FA substrates. In addition, in the absence of NLRX1 mitochondria could oxidize higher concentrations of palmitoylcarnitine (Fig. 4C3). To further explore whether this delay in mitochondrial response to FA substrate is localised in complex I and also is present for carbohydrates, we applied a titration curve for metabolic substrates that only generate NADH for complex I (Fig. 4D1 and D2).

NLRX1 KO hearts showed higher complex I-linked respiration than WT mice under any concentration of NADH-producing substrates (i.e. glutamate, malate, and pyruvate). However, importantly, we did not observe a delay in the response of mitochondrial oxygen consumption to NADH-producing substrates (Suppl. Figure S4B) and the concentration needed to achieve maximal respiration was similar in NLRX1 KO and WT mice, indicating that the delayed responses with NLRX1 ablation were specific for FA carnitines. The protein expression of OXPHOS complexes was investigated and no difference was observed between WT and KO hearts (Fig. 4E). In summary, mitochondria without NLRX1 showed an increased capacity or hyperactivity of mostly complex I without uncoupling, whereas respiration towards FA substrates displayed delayed responses that may be due to complex II activity and/or due to impaired mitochondrial permeability of FA substrates. These mitochondrial alterations due to NLRX1 deletion could not be explained by changes in other OXPHOS complexes.

### No detectable Ca^2+^-induced mPTP opening in NLRX1^−/−^ mitochondria

**Fig. 5:**
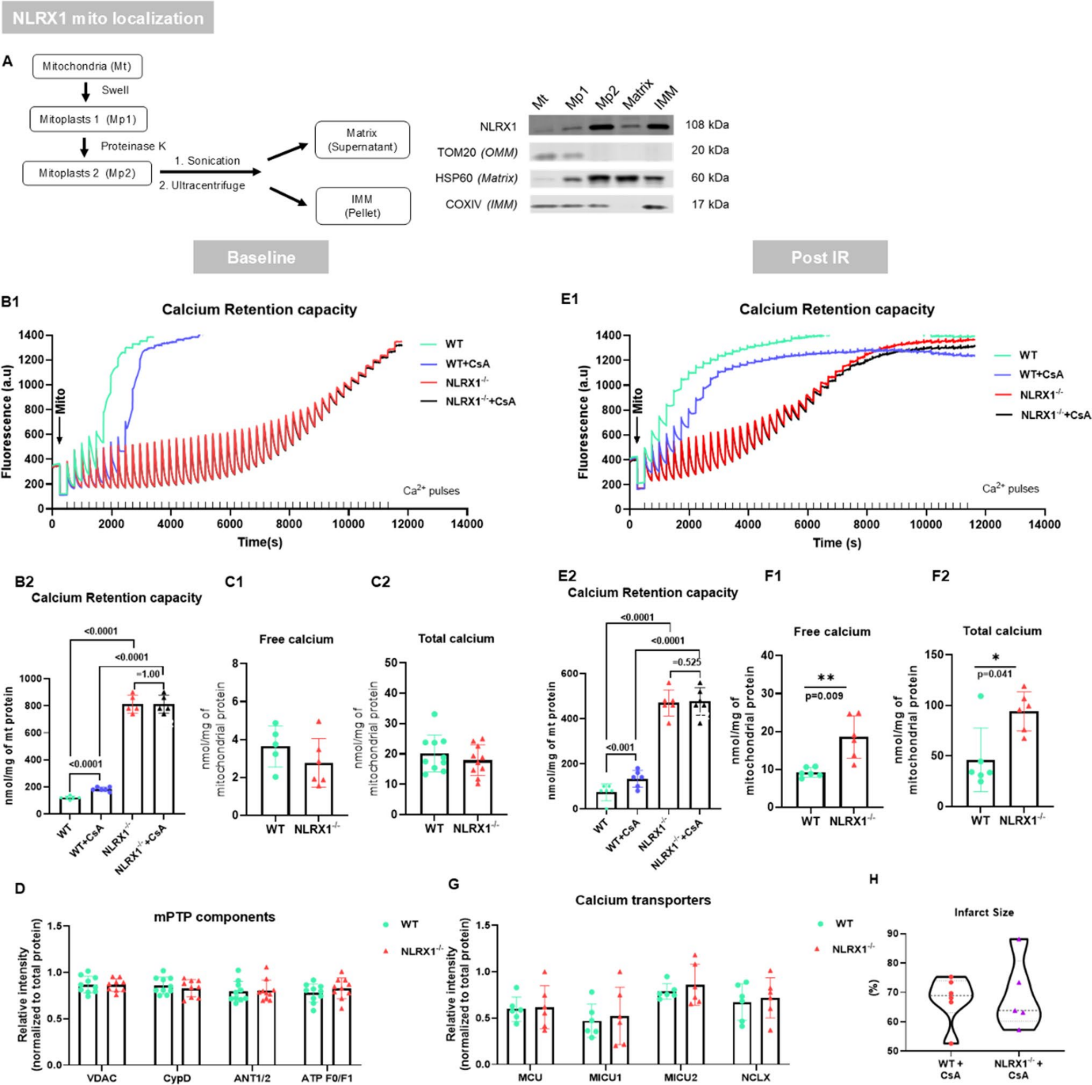
NLRX1 deletion blocks mPTP opening, and NLRX1’s effect on IR injury is dependent on mPTP opening **A**, Mitochondria were isolated from hearts excised directly after in-chest cannulation and further separated into different enrichment fractions, NLRX1 protein was analysed in all sub-fractions by western blot (*n* = 5). **B1-D**, Mitochondria were isolated from the hearts excised directly after in-chest cannulation (Baseline). The sensitivity of mPTP opening was measured by calcium retention capacity assay (**B1**) and analysed (**B2**) in baseline mitochondria (*n* = 6 [WT] and [WT + CsA], 5 [NLRX1^−/−^] and [NLRX1^−/−^ + CsA]). Free calcium content (**C1**, *n* = 5 [WT], 6 [NLRX1^−/−^]) and total calcium content (**C2**, *n* = 10 per group) were measured in baseline mitochondria. **D**, Protein expression analysis of mPTP components in basal mitochondria (*n* = 9–10 per group), following components were examined: voltage-dependent anion channel (VDAC), cyclophilin D (Cyp D), adenine nucleotide translocator (ANT), and ATP F0/F1 proteins (one value missing in KO group for VDAC and Cyp D due to air bubble in lane blot). **E1-G**, Mitochondria were isolated from the hearts after 35 min ischemia and 7min reperfusion (Post IR, *n* = 6 for each group). The sensitivity of the mPTP opening was measured by calcium retention capacity assay (**E1**) and analysis (**E2**) in Post IR mitochondria. Free calcium content (**F1**) and total calcium content (**F2**) were measured in Post IR mitochondria. **G**, Representative analysis of important mitochondrial calcium transporters expression in Post IR mitochondria. The following calcium transporter proteins were examined: mitochondrial calcium uniporter (MCU), mitochondrial calcium uptake 1 and 2 (MICU1 and MICU2), and mitochondrial sodium calcium exchanger (NCLX) proteins. Data shown are mean ± SD. Hearts from WT and NLRX1-/- mice were subjected to IR procedure with cyclosporine A (CsA, mPTP inhibitor) administrated during 20 min baseline and first 60 min reperfusion (*n* = 6 [WT + CsA], 5 [NLRX1-/- + CsA]). CsA equalized IRI between WT and KO, as indicated by infarct size (**H**, % of area at risk) of 2, 3, 5-triphenyltetrazolium chloride (TTC) staining in the hearts after 90 min reperfusion. Data shown are median ± IQ. Statistical significance was evaluated by nonpaired t test (normally distributed data) or Mann–Whitney test (non-normally distributed data). Calcium retention capacity data (**B2** and **E2**) were analysed by two-way repeated measures ANOVA with Bonferroni adjustment for multiple comparisons. *P < 0.05, **P < 0.01 between the groups indicated by the solid lines

The delayed mitochondrial response to palmitoylcarnitine with NLRX1 deletion, together with the increased ratio of p-AMPK/p-mTOR, is indicative of impaired permeability of mitochondria for fatty acids, as was previously also observed when mitochondrial permeability was decreased by porin inhibition [23]. Therefore, we shifted our focus to the mPTP, knowing that this pore also regulates mitochondrial permeability and is heavily involved in cardiac I/R injury. We first focussed on where NLRX1 is actually localised in cardiac mitochondria, for which knowledge is currently missing. We determined the mitochondrial localization of NLRX1 in cardiac cells through sub-fractionation of mitochondria. Using specific markers for each mitochondrial compartment, it is shown that the NLRX1 signal is enriched for mitoplast 2 (= mitochondria fraction without OMM and intermembrane proteins) and the inner mitochondrial membrane (IMM) fraction, suggesting that NLRX1 is mainly localised on the IMM (Fig. 5A). Next, we tested whether NLRX1 affected the mPTP, knowing that this pore is also localised at the IMM and plays a crucial role in cardiac IRI.

#### Baseline condition

Electron microscopy data of intact hearts showed that NLRX1 deletion did not affect gross mitochondrial structure or mitochondria density (Suppl Figure S5A and B). To examine directly whether NLRX1 affects mPTP opening, cardiac mitochondria were isolated from freshly isolated hearts and subjected to calcium retention capacity assay. WT mitochondria showed a clear mPTP opening (sudden increase in the extra-mitochondrial Ca^2+^) after approximately five Ca^2+^ pulses, while this was significantly delayed in the presence of the mPTP inhibitor cyclosporine A (CsA). Remarkably, no mPTP opening was observed in NLRX1^−/−^ mitochondria. In addition, CsA was without any effect in NLRX1^−/−^ mitochondria (Fig. 5B1 and B2). To examine whether the inability of the mPTP to open in the mitochondria without NLRX1 resulted in the accumulation of mitochondrial calcium, total and free amounts of calcium in mitochondria were determined. However, under baseline condition, NLRX1 deletion had no effect on free calcium and total calcium content within the mitochondria (Fig. 5C1 and C2). The absence of mPTP opening in NLRX1^−/−^ mitochondria could not be explained by changes in protein expression of known mPTP components: voltage-dependent anion channel (VDAC), cyclophilin D (Cyp D), adenine nucleotide translocator (ANT), or ATP F0/F1 proteins were similar between genotypes (Fig. 5D). In a separate series of experiments, we also employed phosphoproteome analysis on isolated mitochondria, showing that five proteins (Got2, Cx43, Myl2, Ndufb7, and MICOS10) were observed with decreased phosphorylation in NLRX1^−/−^ mitochondria, and with functional enrichment analysis showing that NLRX1 mainly affects respiratory function, but also transmembrane transport characteristics of the mitochondria (Suppl. Figure S6A and B). Future research will be needed to further explore the functional consequences of these findings.

#### Post IR injury

To examine whether IR stress was able to facilitate mPTP opening in NLRX1^−/−^ mitochondria, mitochondria were obtained from isolated hearts at 7 min reperfusion following ischemia. There was no difference in mitochondria yields (Figure S5C) between genotypes after IR. For WT mitochondria, IR sensitized the mPTP to calcium-induced opening as compared to baseline conditions, and CsA was still able to delay mPTP opening. However, mPTP opening in NLRX1^−/−^ mitochondria was still not observed after IR and CsA remained ineffective (Fig. 5E1 and E2). Examining mitochondrial calcium after IR, both free calcium and total calcium were now significantly higher in NLRX1^**−/−**^ mitochondria compared to WT mitochondria (Fig. 5F1 and F2). These mitochondrial calcium alterations after IR could not be explained by changes in protein levels of important mitochondrial calcium transporters after IR, such as mitochondrial calcium uniporter (MCU), its regulators mitochondrial calcium uptake 1 and 2 (MICU1 and MICU2), or the mitochondrial sodium calcium exchanger (NCLX) (Fig. 5G). Thus, post IR, persistent mPTP pore inhibition in the NLRX1^−/−^ mitochondria remained and was associated with mitochondrial calcium loading that could not be explained by alteration of important Ca-regulation mitochondrial proteins. Finally, it was examined whether NLRX1 affected mitochondrial ROS production, knowing that ROS can be one of the inducers of mPTP. Mitochondria were isolated from WT and NLRX1^−/−^ hearts after ischemia and 7 min reperfusion, but NLRX1 deletion did not affect mitochondrial H_2_O_2_ production at early reperfusion (suppl. Figure 7A and B).

### NLRX1 effects on IR injury is dependent on mPTP opening

Finally, we explored whether the genotype effects on cardiac IRI can be explained by impaired opening of the mPTP in the NLRX1^−/−^ hearts. To test this hypothesis, impaired mPTP opening in the WT was now also induced by CsA treatment, and cardiac IRI was compared between WT and KO hearts both treated with CsA. There was no difference in cardiac functional parameters between CsA perfused NLRX1^−/−^ and WT hearts at T0 (Table S1) or after 20 min normoxic perfusion (Figure S8A through D). Most importantly, all four IRI outcome parameters showed abrogated genotype effects on cardiac IRI: differences in functional recovery (Figure S8E and F) and cell death parameters (Fig. 5H and S8G) between WT and NLRX1^−/−^ hearts were dissipated, indirectly suggesting that NLRX1 effects on whole heart IRI were localised to different regulation of the mPTP.

## Discussion

**Fig. 6:**
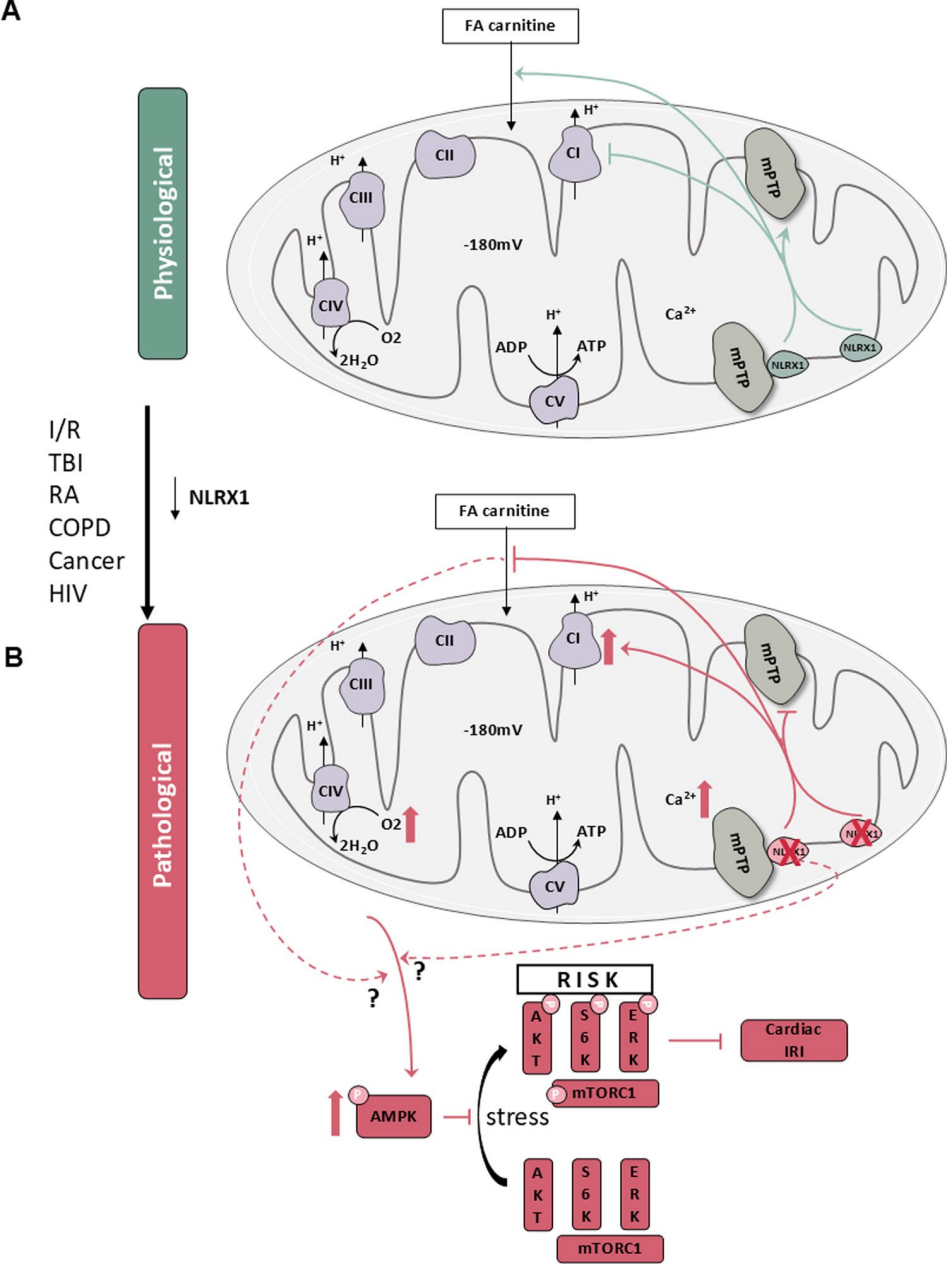
Summary of NLRX1 working scheme. **A**, In physiological conditions, NLRX1 is needed for mPTP regulation and opening, maintaining a proper level of mitochondrial respiratory activity by regulating complex I activity and mitochondrial response to fatty acid (FA) carnitine. **B**, Under pathological conditions (IR [41, 72], Traumatic brain injury (TBI) [73], Rheumatoid arthritis (RA) [32], Chronic obstructive pulmonary disease (COPD) [30], Cancer [12, 77], and HIV [51]), NLRX1 is downregulated. The loss of NLRX1 results in the inhibition of the mPTP, while inducing higher complex I respiratory capacity and slower mitochondrial responses to FA substrates possibly due to impaired mitochondrial permeability of FA. This mitochondrial dysregulation then, possibly due to the impaired inner membrane permeability, activates AMPKα which impairs stress-induced activation of mTOR and the cardioprotective RISK pathway. Further studies are still needed to explore in detail how mitochondrial dysfunction induced by NLRX1 deletion activates AMPKα

We here present novel findings concerning the role of the innate immune receptor NLRX1 within the heart: 1) NLRX1 deletion increased cardiac IR injury due to impaired mTOR/RISK pathway activation at reperfusion, since mTOR/RISK pathway activation was decreased at reperfusion and pharmacological RISK pathway activation by urocortin mitigates NLRX1 effects on IR injury. 2) NLRX1 is needed for healthy mitochondrial function, since NLRX1 deletion caused activation of the energy-deficient sensor AMPK in the intact heart and hyperactivity of mostly complex I and retardation of FA carnitine-induced mitochondrial respiration in permeabilised cardiac fibres. 3) NLRX1 may be a long sought-after crucial component and/or regulator of the mPTP, since it is localised at the IMM, deletion fully blocked mPTP opening and abrogated CsA effects on mPTP opening in isolated mitochondria, and pharmacological mPTP inhibition by cyclosporin A in WT and KO mitigated genotype effects on cardiac IRI. Thus, the present study establishes that the mitochondrial NLRX1 is critically involved in the major cardiac IR cardioprotective pathways of mTOR, RISK, and mPTP. The possible working scheme of NLRX1 is summarized in Fig. 6.

Given that specific NLRX1 agonists are currently in development [75] evaluating these compounds within the recently established small-animal multicenter consortium—designed to test cardioprotective agents against acute cardiac ischemia–reperfusion injury (IRl) [24, 33, 36] appears to be a valuable approach.

### NLRX1 and cardiac IRI

NLRX1 was decreased in patients with acute myocardial infarction [41], and we also observed decreased NLRX1 protein level in post-IR mouse and pig heart. Although our findings demonstrated no Ca^2+^-induced pore opening in mitochondria without NLRX1, cardiac IRI was increased in intact hearts without NLRX1. Thus, in our isolated murine heart model, mPTP inhibition worsened IRI. Whereas mPTP inhibition has most often reported to cause protection against IR, other studies can be found that also reported neutral or opposite effects of mPTP inhibition [29, 52, 58]. Although the ablation of cyclophilin D reduced infarct size following 60 min ischemia in isolated mouse hearts, it actually increased infarct size when a 30 min ischemia period was applied [58]. Another study showed that cyclophilin D deletion protected against 30–45 min ischemia, but not against 60–90 min ischemia in an in vivo model of cardiac IR [29]. Whether mPTP inhibition contributes or protects against cardiac IRI seems to be model-dependent and may also depend, among other factors, on the duration/severity of ischemia examined. This dependency on ischemia duration may also possibly explain why in a large clinical trial CsA failed to be beneficial in AMI patients [52], knowing that ischemia duration is highly variable in AMI patients[19, 81]. Most importantly, however, for the current work was that when mPTP was also inhibited by CsA in WT hearts, difference in IRI between WT and KO disappeared, indirectly suggesting that the different IRI between the genotypes is indeed related to the status of the mPTP.

NLRX1 ablation disturbed mitochondrial function, probably resulting in increased AMPK activity in NLRX1^−/−^ hearts. The increased AMPK activity may be a consequence of impaired permeability of mitochondrial membrane for fatty acids with NLRX1 deletion, as suggested by the slowed mitochondrial response to fatty acid substrates, reminiscent of observations of increased AMPK activity (and decreased mTOR activity) with impaired mitochondrial permeability due to porin inhibition [23]. Increased AMPKα can explain the decrease in mTORC1 and RISK pathway activation after IR, knowing that AMPK controls mTORC and S6 kinase/Akt activity especially during stress conditions [25, 62, 85]. A downregulated mTORC activity can contribute to cardiac IRI possibly through alterations in autophagy [62, 63, 82]. However, no alterations in autophagic markers were observed in our study, possibly because we have focused on acute cardiac IRI (< 2 h reperfusion), whereas most studies report changes in autophagy following longer reperfusion periods.

Additionally, the decreased RISK pathway signalling in the reperfused heart may contribute to the increased IRI in the NLRX1 deleted hearts. The RISK pathway plays a causal role in acute cardiac IRI, and impaired signalling of this pathway exacerbates cardiac IRI [21, 84]. Indeed, pharmacological activation of this pathway in our model by urocortin mitigated NLRX1 effects on cardiac IRI and the activation status of ERK/S6/Akt pathway, suggesting that NLRX1 deletion effects on cardiac IRI are also mediated through differential activation of the RISK pathway. That the RISK pathway is modulated only at reperfusion through AMPK, is comparable with the observation that changes in AMPKα only affect Akt phosphorylation during stress conditions but not during non-stress, e.g. baseline, conditions.

Although NLRX1 is known as an anti-inflammatory NLR which attenuates NF-κB activation and pro-inflammatory cytokine production in multiple chronic diseases [17], no differences in IL-1β, IL-6 or TNFα between WT and NLRX1 deletion were observed in our acute IR heart model. Similarly, NLRX1 was also reported to dampen oxidative stress (4-HNE) in kidney cells 1 day after IR [72]. However, at 90 min after ischemia NLRX1 was without effect on oxidative/nitrative stress. Our data demonstrate that NLRX1 effects on the early phase of acute cardiac IRI are not mediated through increased inflammation or oxidative/nitrative stress.

### NLRX1 and mitochondrial activity

Mitochondrial respiratory activities in permeabilised cardiac fibres demonstrated that the absence of NLRX1 resulted in hyperactivity of complex I, whereas respiration towards FA substrates displayed delayed responses that may be related to impaired mitochondrial permeability towards fatty acids with NLRX1 deletion. These specific mitochondrial alterations could possibly explain why in the intact heart the deletion of NLRX1 shifted cardiac metabolism towards glucose oxidation and away from fatty acid oxidation, together with higher cardiac oxygen consumption [83].

The data are suggestive for that the presence of NLRX1 functioning as a brake in energy turnover, which may not be tissue specific, as increased oxygen consumption in hepatocyte cells[35], increased activity of complex I and complex III in cancer cells[67], increased OXPHOS by regulating mitochondrial genome encoded transcripts for key components of complex I and complex IV[68], and increased OXPHOS in renal epithelial cells[72] were all observed with deletion of NLRX1. The delayed respiratory response of NLRX1^−/−^-mitochondria towards the administration of FA substrates indicates that NLRX1 facilitates FA carnitine handling in cardiac mitochondria. Knowing that the heart derives most of its energy from oxidation of FA [55, 59], this retarded response of the mitochondria within the cardiac fibers could hamper ATP production of the intact heart. Studies in other organs than the heart indeed showed that NLRX1 deletion increased oxygen consumption but decreased ATP level in tubular epithelial cells[72] or resulted in a near-complete loss of mitochondrial ATP-linked respiration in bronchial epithelial cells[31]. Overall, it seems that NLRX1 ablation disrupts normal mitochondrial functioning in the heart, possibly due to impaired permeability of the IMM to fatty acid substrates, which could then also offer an explanation why the energy sensor AMPK is activated, already under baseline conditions, in NLRX1 deleted hearts.

### NLRX1 and the mPTP

The mPTP located in the inner mitochondrial membrane was discovered in 1979 [22]. The functional properties of mPTP are well defined but its exact molecular identity remains uncertain. Several components have been suggested so far: Cyclophilin D [5], adenine nucleotide translocator (ANT) [57], voltage-dependent anion channel (VDAC) [15], phosphate carrier (PiC) [40], FoF1 ATP synthase [1, 8], translocator protein (TSPO) [13]. However, the involvement of several of these components (such as ANT [34], VDAC [6], PiC [18], TPSO [65]) were questioned due to partly preserved or unaltered mitochondrial permeability transition in component-knockout or knockdown models. Thus, “what constitutes the mPTP?” remains the major pertaining question in the field. The present study suggests that NLRX1 may be an important constituent of the pore, because 1) calcium-induced pore opening was absent in isolated mitochondria from NLRX1 KO hearts, 2) CsA was without effect on cardiac IRI and calcium-induced pore opening in NLRX1 KO isolated hearts and mitochondria, respectively, and 3) mitochondrial sub-fractionation studies demonstrated NLRX1 to localize at the mitochondrial location of the pore, the IMM. The loss of pore opening in NLRX1 deleted hearts could not be explained by previously suggested regulators of the pore, because these components were all present in equal amounts between WT and KO hearts. However, we cannot exclude the possibility that changes in assemblage of e.g. FoF1 ATPase occurred in the NLRX1 deleted mitochondria. In addition, the phosphoproteome analysis demonstrated that NLRX1 ablation decreased phosphorylation of specific mitochondrial proteins such as Got2, Cx43, Myl2, Ndufb7 and MICOS10. It is unknown whether any of these proteins may affect the mPTP, for which dedicated gene modulating interventions of these proteins are needed. The increase in mitochondrial calcium at reperfusion in the NLRX1 KO hearts is also an indirect indication that the pore is not present in these hearts, knowing that the mPTP can facilitate calcium release [2]. Finally, it has long been known that fatty acids may trigger mitochondrial swelling and uncoupling for unknown reasons [37]. It is possible that these fatty acids-induced mitochondrial effects are mediated through NLRX1-mPTP interactions, knowing that fatty acid compounds are suggested as specific ligands for NLRX1 [45]. In summary, the data are suggestive of NLRX1 being a novel and crucial component of mPTP. Although other groups have reported NLRX1 localised on the outer mitochondrial membrane in HeLa cells [49] or in the mitochondrial matrix in human embryonic kidney 293T (HEK293T) cells [50], suggestive of NLRX1 function to be cell-type specific and context-dependent [43], the current report indicates for the first time that within the heart NLRX1 is part of the IMM.

## Conclusion

In conclusion, the results demonstrate, for the first time, that the innate immune receptor NLRX1 is localised at the IMM of cardiac mitochondria and is mandatory for mPTP opening, suggesting that NLRX1 may be a long sought-after crucial component of the mPTP. We also demonstrate that the inability to open the mPTP can sensitize the heart to IRI, at least in our isolated mouse heart model, possibly through mitochondrial calcium loading. In addition, NLRX1 is needed for normal functioning of the energy-producing organelles in the heart, the mitochondria. Without NLRX1 present, the energy sensor AMPKα is continuously activated in the heart, thereby preventing mTOR and RISK pathway activation during early reperfusion of the ischemic hearts, contributing to the increased sensitivity of the heart to ischemia–reperfusion injury.

## Supplementary Information

Below is the link to the electronic supplementary material.

Supplementary file1 (DOCX 1926 KB)

Supplementary file2 (PPTX 82440 KB)

## Data Availability

The authors declare that the datasets used and/or analysed in the current study are available from the corresponding author upon reasonable request.
