# Supplementary material for: The innate immune receptor NLRX1 is a novel required modulator for mPTP opening: implications for cardioprotection
Source: Basic Res Cardiol. 2025 Jun 19;120(4):707–25. doi: 10.1007/s00395-025-01124-x (PMC12325489; doi:10.1007/s00395-025-01124-x)
Supplement: Supplementary file 1 — Supplementary file1 (DOCX 1926 KB) [file 395_2025_1124_MOESM1_ESM.docx]

Supplemental Material

**The innate immune receptor NLRX1 is a novel required modulator for mPTP opening: implications for cardioprotection**

Xiao Y, Hu X, Rudolphi CF, Nollet EE, Nederlof R, Wang Q, Bakker D, Panagiota Efstathia Nikolaou, Knol JC, Goeij- de Haas RR^7^, Henneman AA, Pham TV^7^, Jimenez CR^, 8^Grootemaat AE, van der Wel NN, Girardin SE, Kaludercic N, van der Velden J, Onodi Z, Leszek P, Varga ZV, Ferdinandy P, Preckel B, Weber NC, Hollmann MW, Di Lisa F, Zuurbier CJ

This file includes

1. Expanded Materials and Methods

2. Figures S1-S8

3. Table S1-S2

4. Supplemental References 2

**Expanded Methods**

**Animals**

NLRX1^-/-^ mice were generated as reported [4] and bred at our Animal Research Institute AMC (ARIA) institute, Amsterdam UMC, University of Amsterdam. Male NLRX1^-/-^ mice and age (11-28 weeks) and weight matched C57BL/6J wild-type (WT) mice (Janvier labs, France) were used. Mice were housed in a specific pathogen-free facility with ventilated caging system using group housing, with routine checks of each animals’ health status; no mice demonstrated any adverse effects. All mice experimental protocols were registered and approved by the Animal Ethics Committee of the Academic Medical Center, Amsterdam, The Netherlands and conducted pursuant to the Guide for the Use and Care of Laboratory Animals. A total of 198 mice were used for this study, from which 169 mice were included as successful experiments. 29 mice failed to be included due to problems with anesthesia, in-chest aorta cannulation, or proper left ventricle balloon positioning. All experiments were randomized to time of day, such that each genotype had an equal number of experiments performed in the morning and afternoon, to avoid different circadian rhythm effects [2].

The investigation of pig heart tissues conforms to the Guide for the Care and Use of Laboratory Animals published by the US National Institutes of Health (NIH publication No. 85–23, revised 1996), to the EU Directive (2010/63/EU) and was approved by the animal ethics committee of Hungarian National Food Chain Safety Office (SOI/31/26-11/2014).

**Ex vivo mice cardiac IR model**

Induction of myocardial IR injury was conducted as previously reported [3, 4]. Briefly, all mice were anesthetized and heparinized with an intraperitoneal injection of sodium pentobarbital (95 mg/kg body weight) and heparin (15 IU). After confirmation of proper anesthesia with pedal withdrawal reflexes, mice were intratracheally ventilated, then hearts were quickly perfused by in-chest aorta cannulation. Subsequently, excised hearts were connected to Langendorff setup and perfused under a constant flow with Krebs-Henseleit (KH) buffer [ (in mmol/L), 118 NaCl, 4.7 KCl, 1.2 MgSO_4_, 1.2 KH_2_PO_4_, 25 NaHCO_3_, 0.5 EDTA, 2.50 CaCl_2_, 5.5 D-glucose, 0.5 L-glutamine, 1 lactate, 0.1 pyruvate, 1% (g/L) albumin – 0.2mM palmitic acid sodium salt, 0.05 L-carnitine and 30 mU/L insulin]. KH buffer was filtered and gassed with 95%O_2_ / 5%CO_2_ (pH=7.4) and temperature was maintained constantly at 37 ± 0.5 °C. A water filled balloon made of stretched plastic wrap was inserted into left ventricular cavity for continuous assessment of cardiac function throughout whole protocol. All hearts were subjected to 20 min stabilization, during which initial end diastolic pressure (EDP) was adjusted to 3-5 mmHg and initial perfusion pressure (Pperf) was attained to 75-85 mmHg. Hearts were excluded, a priori, when coronary flow was above 4 ml/min to achieve proper initial Pperf and/or developed left ventricular pressure (DLVP; LV systolic pressure - diastolic pressure) was below 80 mmHg, and/or heart rate (HR) was below 280 beats per minute (bpm), and/or extended period of arrhythmias after 20 min of stabilization. After stabilization, hearts were subjected to 20 min baseline normoxic perfusion. Afterwards hearts were immediately collected (Protocol 1), or continuously subjected to 35 min no flow ischemia and 90 min reperfusion (Protocol 2). For the RISK activator experiments, 2nM Urocortin (#U9507, Sigma-Aldrich) was administrated during 15min baseline (T0 to T17, Protocol 3). For mitochondria isolation, hearts were subjected to 20 min baseline perfusion followed by 35 min I and 7 min R (Protocol 4). For the mPTP inhibitor experiments, 500nM Cyclosporin A (# 239835, Sigma-Aldrich) was infused from a syringe pump through a mixing chamber just above the heart during a 20min baseline and the first 60min reperfusion (Protocol 5).

Rate pressure product was calculated from DLVP × HR. For all western blot analysis, hearts were immediately frozen in liquid nitrogen and stored at -80°C. Following 24 h freeze-drying, dry heart weight was determined, the left ventricle (LV) separated and stored at −80°C until further western blot processing.

**Porcine infarction model and tissue collection**

The porcine myocardial IR model was performed as previously described [1]. Briefly, domestic female pigs (25-35kg; University of Kaposvár, Hungary) received an intramuscular injection of ketamine hydrochloride, xylazine, and atropine (12mg/kg, 1mg/kg and 0.04mg/kg, respectively), then anesthesia was maintained with isoflurane oxygen mix (2–2.5 vol% and 3 L/min). 6F sheaths (Medtronic Inc, Minneapolis, MN) were inserted into the femoral artery to further have entry routes for the catheterization, and 5000–5000 IU unfractionated heparin was administered. A balloon catheter (2.75 mm diameter, 8 mm length) was placed in the mid part of the left anterior descending coronary artery (LAD) after the origin of the 2nd diagonal branch. The intracoronary balloon was inflated with 5 atm for 90 min, followed by deflation of the balloon, resulting in 3 h of reperfusion which was confirmed by coronarography. After the reperfused infarction procedure, the wounds were closed and anesthesia was terminated by the withdrawal of isoflurane. Animals were administered by an antibiotic cocktail containing 100 mg benzathine benzylpenicillin, 100 mg procaine benzylpenicillin, 200 mg dihydrostreptomycin-sulphate before recovery, and intramuscular injections of 1 g metamizole for analgesia. After 3 h reperfusion or 3 days, animals were euthanized under general anesthesia induced by intramuscular injection of ketamine hydrochloride, xylazine, and atropine (12mg/kg, 1mg/kg and 0.04mg/kg, respectively) with an intravenous injection of 10% potassium chloride solution. The explanted hearts were placed in ice-cold saline, and small parts of the ischemic and non-ischemic areas were collected. The samples were frozen in liquid nitrogen and kept at −80°C until further processing. Two animals were excluded from the final data set due to significant procedural deviations. These animals experienced substantial failures during the procedure, including markedly shorter ischemia or reperfusion times due to premature death or severe arrhythmias.

**Lactate dehydrogenase (LDH) measurement**

The coronary effluent was collected at 10min of reperfusion and immediately stored at -80°C for further analysis. 800 µl LDH assay (containing 0.17 mM NADH, pH=7.5) and 12.5 µl pyruvate (50 mM) were added to 200 µl effluent samples. The formation of NAD^+^ from NADH was monitored over 4 min by spectrophotometry at 340 nm and 25°C, and LDH activity as index of cell death was blindly determined.

**Infarct size determination**

At the end of 90min reperfusion the hearts were weighted, a small part of the apex was quickly frozen for protein analysis, and the major part of the heart was stored at -20°C. 2,3,5-triphenyltetrazolium chloride (TTC, Sigma-Aldrich, St. Louis, MO, USA) staining was performed within one week to determine the infarct size. Frozen heart was cut into 1mm thick slices across the short, transverse axis and quickly immersed in 1% TTC solution (PH=7.4) on a shaker (37°C, 300 rpm) for 20 min. Then slides were transferred into 4% formaldehyde (PH=7.4) for 2 h at RT. After scanning, percentage of infarct size [%, relative to ventricular area (excluding atria)] was quantified and analyzed with SigmaScan Pro5 software by an investigator not aware of treatment allocation.

**Measurement of mitochondrial respiratory activities**

Mitochondrial function was assessed using respirometry by an investigator not aware of genotype allocation. Hearts were excised following aorta cannulation and perfused with high potassium KH buffer containing (in mmol/L), 118 NaCl, 30 KCl, 1.2 MgSO_4_, 1.2 KH_2_PO_4_, 25 NaHCO_3_, 0.5 EDTA, 2.25 CaCl_2_, 5.5 D-glucose. The left ventricle free wall was quickly cut and placed in ice cold BIOPS buffer (containing (in mM) K2EGTA (7.2), CaK2EGTA (2.8), ATP (5.8), MgCl2 (6.6), taurine (20), phosphocreatine (15), imidazole (20), dithiothreitol (0.5) and 2-(N-morpholino) ethanesulfonic acid (50); pH 7.1 adjusted with KOH). Fresh left ventricular fiber bundles (≈2 mg) were gently separated using ultra-thin forceps and permeabilized in ice-cold BIOPS solution containing 50 µg/mL saponin for 25 min. Next, fiber bundles were washed twice for 10 min in ice-cold mitochondrial respiration medium (MiR05), containing (in mM) EGTA (0.5), MgCl_2_ (3), potassium lactobionate (60), taurine (20), KH_2_PO_4_ (10) HEPES (20), sucrose (110) and 1 g/L fatty acid free bovine serum albumin, pH 7.1 adjusted with KOH, rapidly blotted dry, weighed and inserted into a high-resolution respirometer (Oxygraph-2k; Oroboros Instruments). All experimental protocols were performed *in duplo* at 37°C under oxygen levels above 300 µM throughout the experiment to avoid oxygen supply limitations.

*Oxidative phosphorylation*

Leak respiration was measured using 10 mM sodium glutamate, 2 mM sodium malate and 5 mM sodium pyruvate, providing electron input into complex I via NADH. Maximal NADH-linked respiration was measured upon addition of 5 mM ADP. Cytochrome-*c* (10 µM) was injected to evaluate outer mitochondrial membrane integrity. Total OXPHOS capacity, with maximum electron input through complexes I and II was assessed after adding 10 mM succinate. Carbonyl cyanide p-trifluoro-methoxyphenyl hydrazine (FCCP) was titrated in 0.25 μM steps to uncouple respiration from complex V, thus measuring excess capacity of the electron transferring complexes (I-IV). Rotenone (0.5 µM) was added to block complex I and assess succinate-linked respiration through complex II. Antimycin-A (2.5 µM) was injected to fully block mitochondrial oxygen consumption and measure residual oxygen consumption, which was subtracted from all values as background.

*NADH-driven oxidation and kinetics*

To assess kinetics of NADH-driven respiration, we measured oxygen consumption in response to incrementally increasing concentrations of the NADH-producing substrates glutamate, malate and pyruvate. To this end, first ADP (5 mM) and cytochrome-*c* (10 µM; to avoid respiration limitations due to any efflux of cytochrome-*c*) were added. Then a master mix containing 1 M glutamate, 0.2 M malate and 1 M pyruvate was titrated in 1-2 µL steps until maximum respiration was achieved. The rate of oxygen consumption before adding NADH-producing substrates was subtracted from all values as background.

*Fatty acid oxidation and kinetics*

To evaluate functional changes in fatty acid oxidation in WT and KO mice, we measured the capacity to oxidize the activated fatty acid palmitoylcarnitine. First a low concentration of malate (0.1 mM) and ADP (5 mM) were added. Malate is converted by malate dehydrogenase to oxaloacetate which is needed for condensation with β oxidation-derived acetyl-CoA into citrate, which is needed to prevent accumulation of acetyl-CoA and consequent inhibition of β oxidation. Next, cytochrome-*c* (10 µM) was added. Palmitoylcarnitine was then titrated in 5-10 µM steps until maximum respiration was achieved. Baseline respiration induced by malate and ADP was subtracted from all values as background.

**Electron Microscopy**

Electron microscopy was performed to evaluate mitochondria density and structure. Briefly, LV tissues were cut into longitudinal strips and fixed for 4 hours at room temperature in 1x PFA+ GA buffer with 2% paraformaldehyde (PFA) plus 0.2% glutaraldehyde (GA) in 0.1 M PHEM buffer (60 mM PIPES [piperazine-N,N′-bis 2-ethanesulfonic acid], 25 mM HEPES, 2 mM MgCl_2_, and 10 mM EGTA at pH = 6.9). Then samples were transferred to storage buffer (0.1 M PHEM with 0.5% PFA) in 4 °C. After washing, tissue was embedded and sectioned for imaging. Mitochondria number was counted in 4 WT hearts and 4 KO hearts. In brief, a grid size of 0.3 × 0.3 µm was used for each image, intersection points counting (points hitting the mitochondrion) was used for estimation of mitochondrial density and normalized to total image area (µm^2^). Twenty different images were quantified and the average was calculated for each heart.

**Isolation and subfractionation of cardiac mitochondria**

Mitochondria were isolated from the hearts excised directly after in-chest cannulation with isolation buffer A or after ischemia (35min)-reperfusion (7min) with KH buffer, all isolation steps were performed on ice. Briefly, heart was placed and rinsed in ice cold isolation buffer A (mM: 200 mannitol, 50 sucrose, 5 MOPS, 5 KH_2_PO_4_, 1 EGTA, pH=7.4). After removing fat and atria, heart ventricular tissue was minced with a blade quickly to obtain a homogeneous product. Protease solution (0.5 U/ml, # P8038,Sigma, dissolved in isolation buffer A) was used for digesting tissue for 10 min, afterwards isolation Buffer B (isolation buffer A + 0.1% Bovine Serum Albumin, pH=7.4) was added to neutralize the protease, digested tissue then recovered by low-to-medium speed centrifugation at 300g for 1 min at 4 °C. Pellet was resuspended in isolation buffer B and homogenized with a motorized dounce homogenizer with a pre-chilled pestle (6 passes at 1,200 rpm) in container with ice and water. Homogenate was transferred into new tube and centrifuge at 600 g for 10 min at 4 °C to pellet nuclei and tissue debris. Supernatant was transferred and centrifuged at 8000 g for 15 min at 4 °C to get mitochondria pellet, which was then resuspened in isolation buffer A only with 0.2mM EGTA and centrifuged (8000 g, 15 min,4 °C) again. Final mitochondria pellet was resuspended and stored in isolation buffer A only with 0.2mM EGTA for further processing. Mitochondrial protein concentration was measured using the Lowry assay.

For subfractionation, crude mitochondria were first resuspended and incubated in hypotonic solution (20 mM Tris-Cl, pH=7.4) on ice for 20 min. Possible residue of outer mitochondrial membrane was digested with 100 µg/mL proteinase K (Sigma P4850) at 4°C for 30 min, this proteolytic reaction was then blocked by 5 mM phenylmethylsulfonyl fluoride (PMSF). The obtained mitoplasts were sonicated and centrifuged at 100,000 g for 30 min at 4 °C to get the matrix fraction (supernatant) and inner mitochondrial membrane fraction (pellet). All the fractions were resuspended in isotonic buffer (250 mM sucrose, 20 mM HEPES, 1mM EDTA) for further processing.

**Calcium Retention Capacity**

We measured calcium retention capacity (CRC) in the isolated mitochondria which could provide important information on the resistance of the mitochondria to Ca^2+^-induced mPTP opening. Mitochondria pellet was suspended in calcium retention capacity assay (mM: 200 mannitol, 50 sucrose, 5 MOPS, 5 KH_2_PO_4_, 5 Glutamate, 5 Malate, pH=7.4) to obtain 0.25 mg/ml mitochondria suspension, then 0.5 µM membrane impermeable Calcium Green-5N probe (C3737, Invitrogen™) was added and fluorescence signal was determined with Ex/Em = 485/532 nm at 25 °C. 5 μM CaCl_2_ pulses were applied every 4 min, each calcium addition could induce a spike in signal then the spike dissipated due to the uptake of Ca^2+^ into the mitochondrial matrix. The extra-mitochondrial Ca^2+^ concentration started accumulating with increasing Ca^2+^ loading. Decreased signal spike dissipation can be observed which reflects lower capacity of Ca^2+^ uptake. This was then followed by a sustained fluorescence signal increase indicating a massive release of the mitochondrial Ca^2+^ caused by the mPTP opening. The CRC was defined as the total amount of CaCl_2_ required to trigger this massive increase of fluorescence signal or no further decrease of signal and was expressed as nmol per mg of mitochondrial protein.

**Mitochondrial calcium measurement**

For calcium measurements, only plastic cuvettes with no calcium leakage and milli-Q water with low calcium concentration in the range of ppt were used. Calcium measurements were corrected for fluorescence signals from the lysis buffers without mitochondria being present. For free calcium measurement, mitochondria pellet was washed twice in isolation buffer A without EGTA directly after isolation. Samples were then incubated with 1% Triton-X100 for free calcium measurement. Mitochondria pellet without EGTA was incubated in 1M HCl to dissolve matrix calcium salt for total calcium content measurement. After incubation, samples were sonicated (2 times 10s, at 40% of maximal power output) on ice. Pellet was discarded by centrifuge (8000 g, 15 min,4 °C), supernatant was then fluorometrically determined with 3.7µM Calcium Green-5N probe (Ex/Em = 485/532 nm, 25 °C, pH=6.8). Calcium concentration was calculated from a calcium standard curve and expressed as nmol per mg of mitochondrial protein.

**Coimmunoprecipitation (CoIP)**

For endogenous CoIP measurement, additional WT and NLRX1^-/-^ mice were subjected to IRI protocol. After 90 min reperfusion, LV tissues were snap frozen and stored in -80°C. Afterwards, LV tissues were homogenized in pre-chilled pestle containing CoIP buffer [20 mM HEPE, 120 mM NaCl, 1 mM EDTA, 1X HaltTM Protease & Phosphatase Inhibitor] with 0.3% CHAPS (SKU 1116620001, Sigma- Aldrich) for 6 strokes at 1200rpm. Samples were centrifuged for 2 minutes at 10000 RCF and 4 °C. Lowry assay was used for protein content measurement. 16 µl protein of each sample (1200 µg/ml) were stored at -80°C as input samples. To prepare CoIP samples, 600 µg protein per sample was diluted with CoIP buffer to a total volume of 200 µl, 4 µL antibody (mTOR, #2972, CST) was added and incubated overnight at 4°C with continuous rotation. Then, 20 µL Dyna beads (#10003D, Invitrogen) were washed three times in ice cold PBS (pH=7.4) with a magnetic rack. Immune complexes were added to the beads and incubated at 4°C for 2 h with rotation. Then samples were washed for 6 times (3 times with CoIP buffer, 3 times with PBS), supernatant was removed, 20 µL loading buffer (2X) was added to CoIP samples. 4µL loading buffer (5X) was added to input sample, all samples were denatured by heating for 5min at 95°C, then loaded on the gel, then Raptor, Rictor, mLST8, NLRX1 and α-Tubulin (Supplementary Table 1) were detected following western blot procedures.

**Western blotting**

Heart tissues, isolated mitochondria or mitochondrial fractions were homogenized with 0.02 M HEPES, 0.25 M Sucrose (or 1× radio immunoprecipitation assay buffer) supplemented with 1X HaltTM Protease & Phosphatase Inhibitor (Thermofisher, #78442). Samples were sonicated and then incubated with 1% Triton-X 100 for 10 min on ice. Afterwards, samples were centrifuged at 10000 RCF and 4 °C, the supernatant was collected for following experiments. Protein concentration was determined by Lowry assay or bicinchoninic acid assay kit (Thermo Scientific, Waltham, MA). Equal amount of protein from each sample were electrophoresed onto a 4–12% precast polyacrylamide gel (Biorad, #345-0125) and transferred to polyvinylidene fluoride (PVDF) membrane. After incubation with blocking buffer (Odyssey) at room temperature (RT), membrane was probed with primary antibody (listed in Table S2) over night at 4ᵒC. Membrane was washed three times and incubated with the complementary secondary fluorescence antibody (IRdye, Licor, Lincoln, USA, 1:5000) for 1h at RT. Membrane was washed for three times and scanned with Odyssey scanner (Li-cor). For Porcine samples, membranes were incubated with corresponding HRP-conjugated secondary antibodies (Cell Signaling Technology) for 2h, signals were visualized after incubation with Clarity™ Western ECL Substrate enhanced chemiluminescence kit (Bio-Rad #1705060) by Chemidoc XRS+ (Bio-Rad, Hercules, CA, US). Image analysis was performed using Image Lab™ software (Bio-Rad, Hercules, CA, US). Coomassie blue (#161-0786, Bio-safe) staining of membrane was performed and total protein amount was quantified.

**Phosphoproteome of isolated mitochondria**

Preparing lysates and digests

Mitochondria were isolated from the hearts, and a mitochondrial pellet was obtained as described above in isolation of mitochondria section. Washed mitochondria pellets were lysed in 8 M urea buffer (8 M urea/20 mM HEPES (pH 8.0)) lysis buffer containing 1 mM sodium orthovanadate, 2.5 mM sodium pyrophosphate, and 1 mM β-glycerophosphate and sonicated for 3 times 10 seconds with cooling on ice (Branson sonicator, maximum output (~20W)). The clear supernatant after centrifugation (5 min, 16000g at RT) was used for digestion after protein determination (micro BCA assay, Pierce). 200 ug of proteins were reduced with DTT (dithiothreitol, 5 mM) and alkylated with IAA (iodoacetamide, 10 mM) followed by dilution to 2 M urea. Samples were digested with Sequencing Grade Modified Trypsin (Promega, Leiden, The Netherlands) overnight (1 : 50 ratio) and thereafter acidified by addition of 20% TFA (1% end concentration). The digests were desalted using Oasis HLB columns (10 mg capacity; Waters, Milford, MA, USA) and eluted in 200 µl 80% ACN/0.1% TFA and used for IMAC.

IMAC (Immobilized Metal-ion Affinity Chromatography)

Global phosphoproteomics was performed following the Phospho Enrichment v2.0 protocol on the AssayMAP Bravo platform. Phosphorylated peptides were enriched using 5 μL Fe(III)-NTA cartridges (Agilent Technologies, Middelburg, Nederland). The cartridges were primed with 100 μL (100% ACN/ 0.1% TFA) at a flow rate of 300 μL/min and equilibrated with 50 μL loading buffer (80% ACN/0.1% TFA) at 10 μL/min. The samples were loaded onto the cartridge at 3.5 μL/min. The columns were washed with 50 μL loading buffer and the phosphorylated peptides were eluted with 25 μL 5% ammonia/30%ACN and dried in a vacuum centrifuge at 45°C. Thereafter the dried phosphopeptides were dissolved in 60 μL 0.5%FA/4%ACN. 20 µl (~600 ng) was used to prepare EVO tips (Evosep Biosystems,Denmark). The tips were stored at 4°C until LC/MS measurement.

Protein and phosphosite quantification by mass spectrometry

Eluting peptides were ionized and introduced into a timsTOF HT mass spectrometer (Bruker). The MS data was acquired in data-dependent acquisition mode. The raw TIMS data were directly loaded into MaxQuant version 2.6.2.0 to search against a mouse Uniprot reference proteome (canonical plus isoforms, 25478 entries). Enzyme specificity was set to trypsin. Cysteine carboxamidomethylation (+57.021464 Da) was treated as a fixed modification and serine, threonine, and tyrosine phosphorylation (+79.966330 Da), methionine oxidation (+15.994915 Da), and N-terminal acetylation (+42.010565 Da) as variable modifications.

Data analysis

Data analyses at the phosphosite level were performed using the __1 (single phosphorylation), __2 (double phosphorylation), and __3 (≥3 phosphorylations) intensity levels in the Phospho (STY [serine, threonine, tyrosine]). Proteins harboring significantly differential phosphosites (p<0.05) were uploaded to the STRING tool version 12.0 [5], to generate a protein-protein association network with confidence edges. Functional enrichment analysis was conducted in the STRING tool.

**Cytokines Determination**

IL-1β, IL-6 and TNFα were determined in LV tissues after baseline as well as after 90min reperfusion using ELISA kits (#DY401, #DY406 and #DY410, R&D Systems) following manufacturer’s instructions. Final quantification was normalized to total protein concentration as determined by Lowry assay.

**Statistical analyses**

Results were expressed as Mean ± SD or Median ± IQ (violin plot), n is sample size representing the number of biological replicates which is shown in figure legends. Sample sizes were determined based on accepted standards in the field, previously published literature, and our experience in observing statistically significant differences of similar effects in mice hearts. Shapiro–Wilk test was used to test the normality distribution of data. Comparisons of two groups: Student *t* test was performed when data was normally distributed. Non-normally distributed data was analyzed with independent-samples Mann-Whitney U test. Comparisons with more than two groups were evaluated by one-way ANOVA followed by Games-Howell multiple comparisons test. Calcium retention capacity data and NADH curve was analyzed by Two-way repeated measures ANOVA with Bonferroni adjustment for multiple comparisons. Statistics were conducted using IBM SPSS statistics version 26 (International Business Machines Corp., Armond, NY, USA). Figures were made in GraphPad Prism 8.0 (GraphPad Software, Inc., La Jolla, CA, USA). In all tests, significance was accepted for *P* < 0.05.


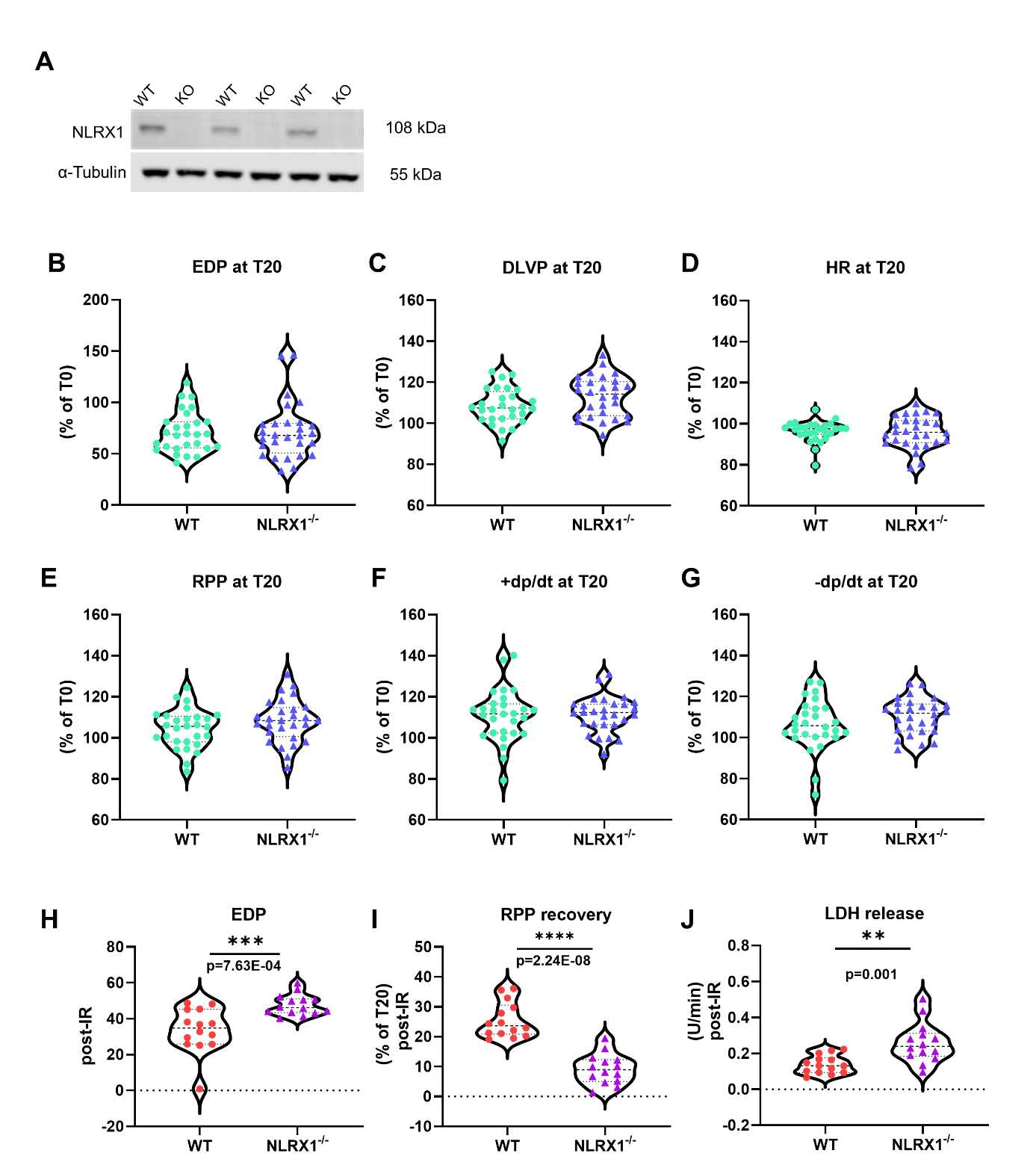


**Figure S1. NLRX1 deletion aggravates cardiac IRI without affecting baseline mechanical function.
(A-G)** Hearts from WT and NLRX1-/- mice were subjected to 20min normoxic perfusion**. A,** NLRX1 protein expression was detected to confirm the deletion in mouse hearts. Representative immunoblots of NLRX1 and α-Tubulin as loading control were shown (n=8 per group). **B-G**, Hearts from WT and NLRX1^-/-^ mice were subjected to 20min normoxic perfusion (n=28 per group). For all basal functional parameters, the change at T=20 min relative to the value at T=0 min is depicted. Mechanical function was indicated by end diastolic pressure (**B**, EDP); Developed left ventricular pressure (**C**, DLVP); Heart rate (**D**, HR); Rate pressure product (**E**, RPP = DLVP X HR); Maximum contraction rate of left ventricle (**F**, +dp/dt); and Maximum relaxation rate of left ventricle (**G**, -dp/dt). **(H-J)** Hearts from WT and NLRX1^-/-^ mice were subjected to 20min normoxic perfusion (Baseline) followed by 35min ischemia and 90 min reperfusion (Post IR). More severe IRI in NLRX1^-/-^ than in WT evident from increased end diastolic pressure (**H**, EDP) at 90 min reperfusion; decreased rate pressure product (RPP) recovery (**I**, % of RPP at 90 min reperfusion [T=145 min] related to RPP at baseline [T=20 min]) and increased lactate dehydrogenase (LDH) release in coronary effluent (**J**, normalized to coronary flow), (n=14 per group). Data shown are median ± IQ. Statistical significance was evaluated by non-paired t-test (normally distributed data) and Mann-Whitney test (non-normally distributed data).

**
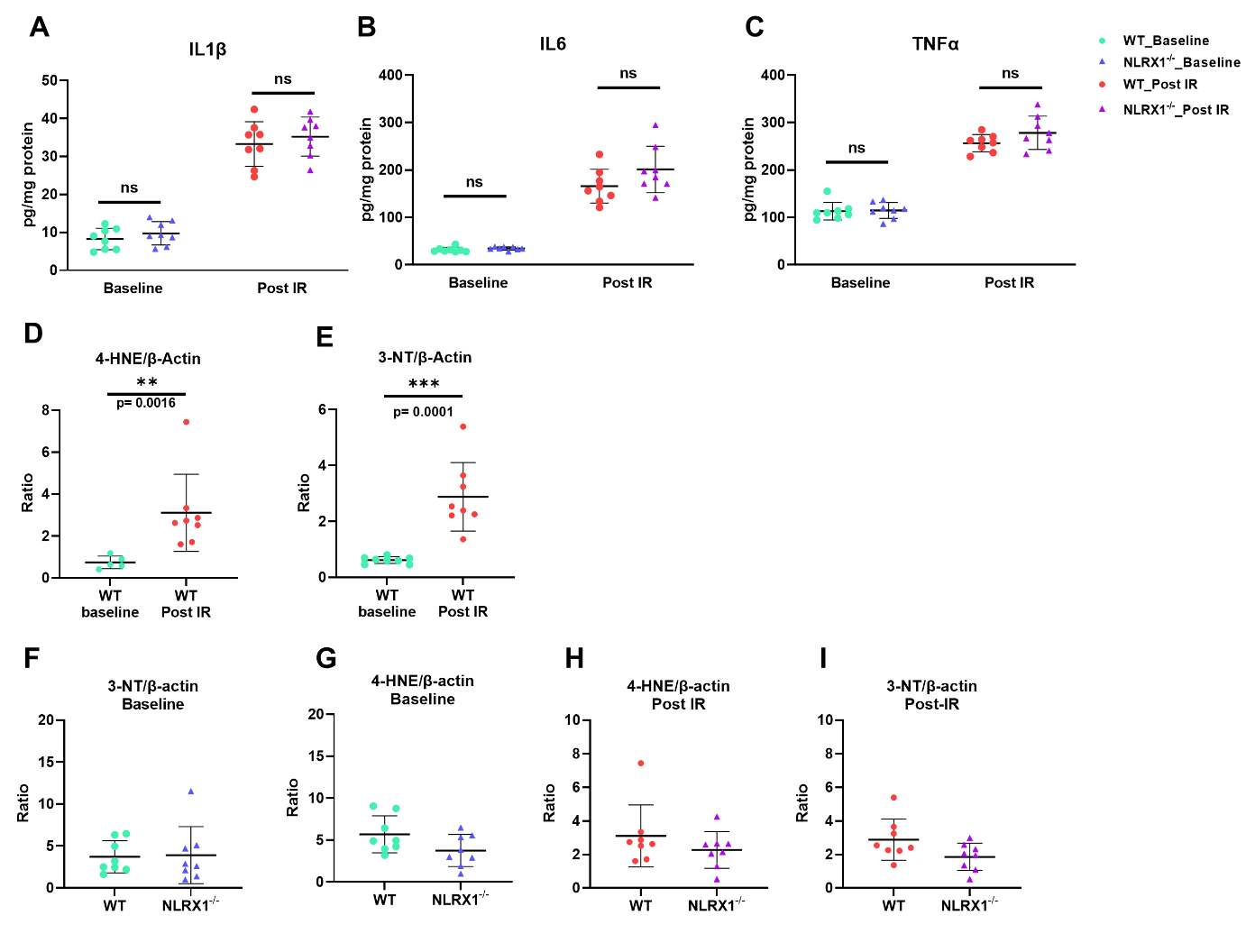
**

**Figure S2. NLRX1 deletion has no effect on inflammation or oxidative stress during acute cardiac IRI.** Heart tissues from WT and NLRX1^-/-^ mice were collected and homogenized after ex vivo 20min normoxic perfusion (Baseline) followed by 35min ischemia and 90 min reperfusion (Post IR). **A**-**C**, Inflammatory cytokines IL 1β (**A**), IL6 (**B**) and TNFα (**C**) in heart tissues at baseline as well as post IR were measured by ELISA kits (n=8 per group). **D**, Representative expression analysis of 4-HNE in WT hearts at baseline vs post IR (n=5 [WT_Baseline], 8 [WT_Post IR]). **E**, Analysis of 3-NT protein expression in WT hearts at baseline vs post IR (n=8 per group). **F** and **G**, Representative immunoblots’ analysis of 4-HNE and 3-NT in WT and NLRX1^-/-^ hearts at baseline (n=8 per group). **H** and **I**, protein expression analysis Expression of 4-HNE and 3-NT in WT and NLRX1^-/-^ hearts after IR (n=8 per group). Data shown are mean ± SD. Statistical significance was evaluated by nonpaired t test (normally distributed data) and Mann-Whitney test (non-normally distributed data). ****P*<0.001 between the groups indicated by the solid lines.


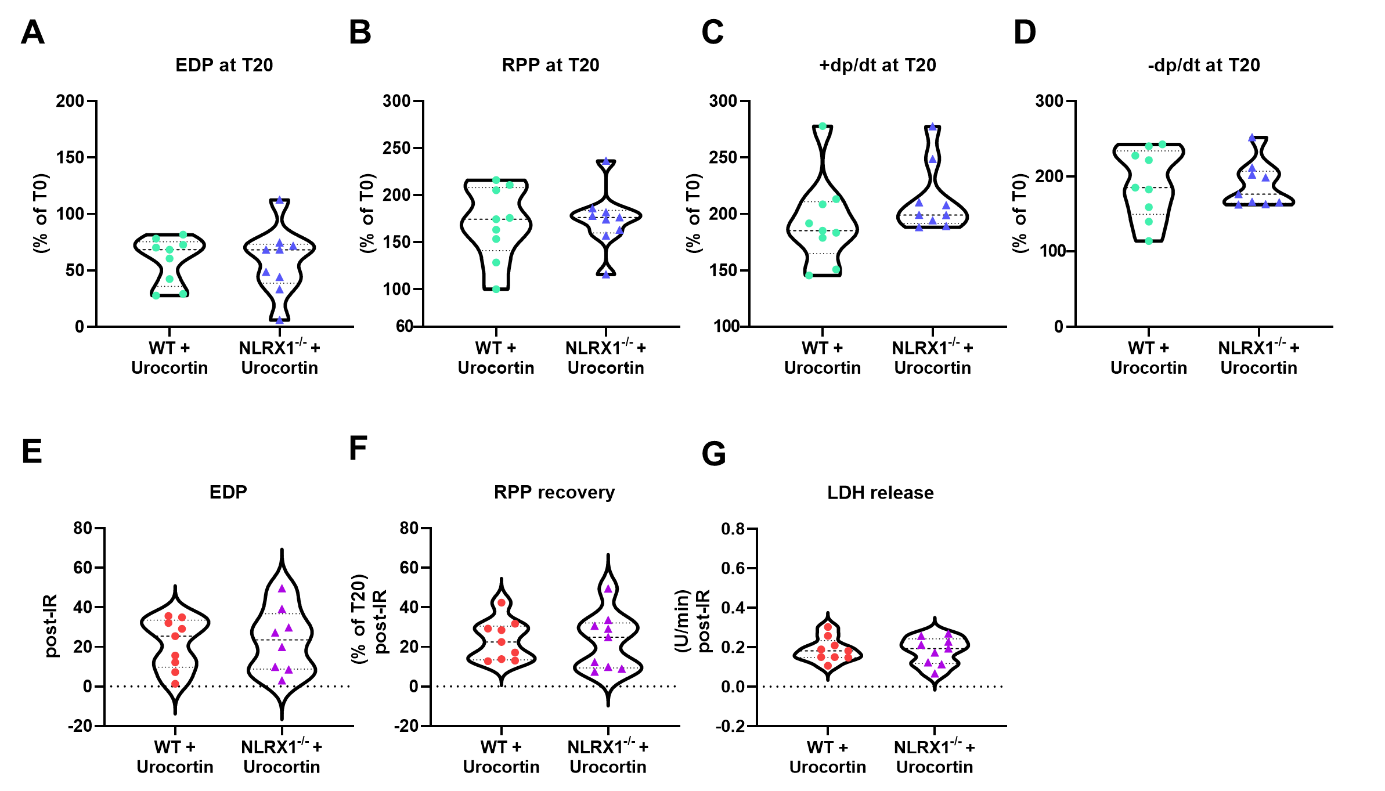


**Figure S3. RISK activator urocortin abrogates genotype effects on IRI.** Each heart was perfused with 2 nM urocortin during baseline, then subjected to IR procedure (n=9 per group). **A-D**, For all basal functional parameters, the change at T=20 min relative to the value at T=0 min was depicted. Mechanical function was indicated by end diastolic pressure (**A**, EDP); Rate pressure product (**B**, RPP = DLVP X HR); Maximum contraction rate of left ventricle (**C**, +dp/dt); and Maximum relaxation rate of left ventricle (**D**, -dp/dt. **E,** End diastolic pressure (EDP) at 90 min reperfusion (T=145 min; one EDP value in the NLRX1-/- + Urocortin group was excluded because of negative value). **F**, Rate pressure product (RPP) recovery (% of RPP at 90 min reperfusion [T=145 min] related to RPP at baseline [T=20 min]). **G**, LDH release in coronary effluent (normalized to coronary flow). Data shown are median ± IQ (G-J). Statistical significance was evaluated by nonpaired t test (normally distributed data) or Mann-Whitney test (non-normally distributed data).


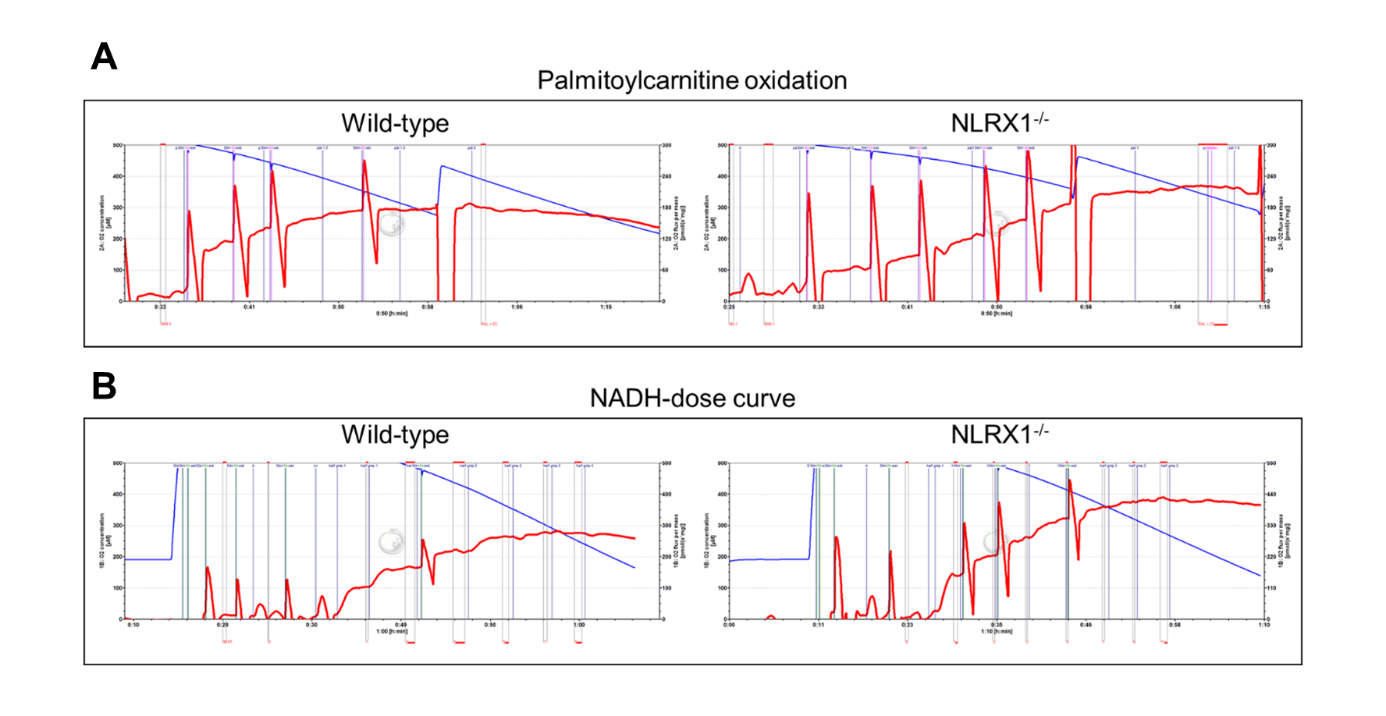
**Figure S4: Representative traces of palmitoylcarnitine oxidation (A) and NADH titration (B) measurements, showing slowed responses of mitochondrial oxygen consumption to palmitoylcarnitine but not to NADH substrates, indicative of impaired mitochondrial permeability for palmitoylcarnitine with NLRX1 deletion.** Note that the large artefacts that are seen are the result of briefly (2 sec) switching the stirrer bar off and on again, in order to keep the tissue fiber from sticking to the stirrer bar. In NLRX1 KO mice, more injections of palmitoylcarnitine were needed to achieve maximal respiration, and the responses were also delayed. With respect to gradually increasing NADH-linked respiration we observed that under any concentration of NADH-producing substrates NLRX1 KO mice displayed higher respiration values, but the mitochondrial oxygen response was not delayed.


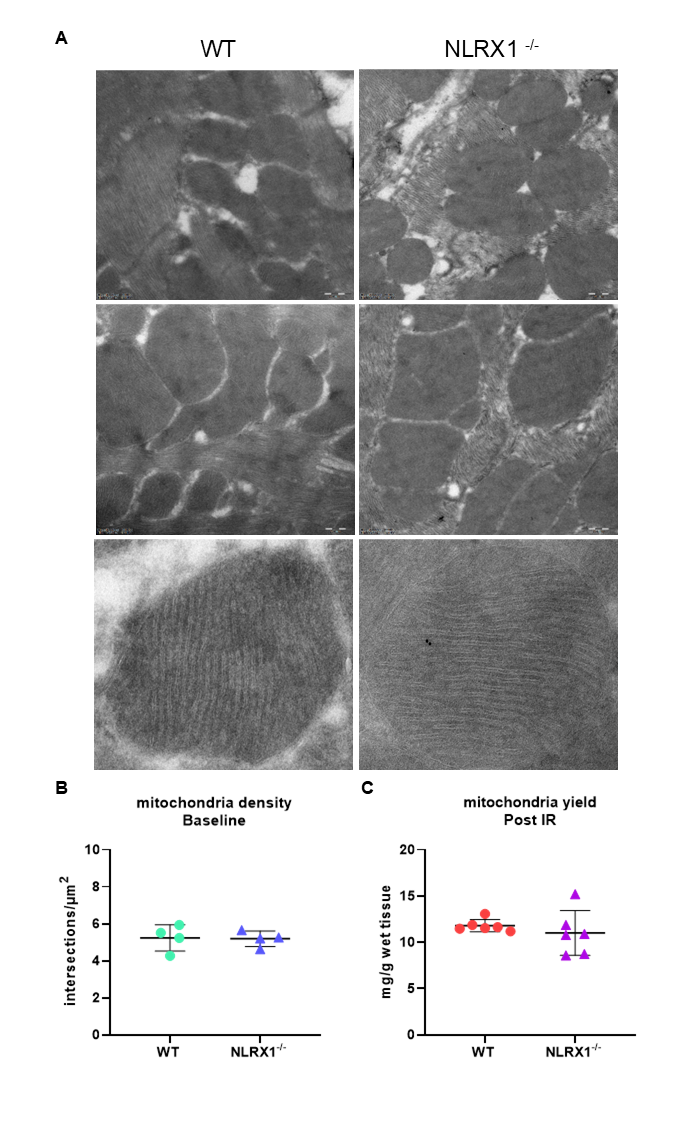


**Figure S5. NLRX1 deletion does not affect mitochondrial gross structure or density. A** and **B,** Left ventricular tissues were isolated from the hearts excised directly after in-chest cannulation (Baseline). Typical electron micrographs of left ventricular tissues from WT and NLRX1^-/-^ mice (**A**, scale = 500 nm) and mitochondrial density was quantified (**B**, n=4 per group [4 hearts per group, 20 images were quantified per heart]). **C**, Mitochondria were isolated from the hearts after 35 min ischemia and 7min reperfusion (Post IR injury) and the total protein amount of mitochondrial fraction (mitochondrial yield) was quantified (n=6 per group) and normalized to wet tissue weight. Data shown are mean ± SD. Statistical significance was evaluated by nonpaired t test (normally distributed data).

**
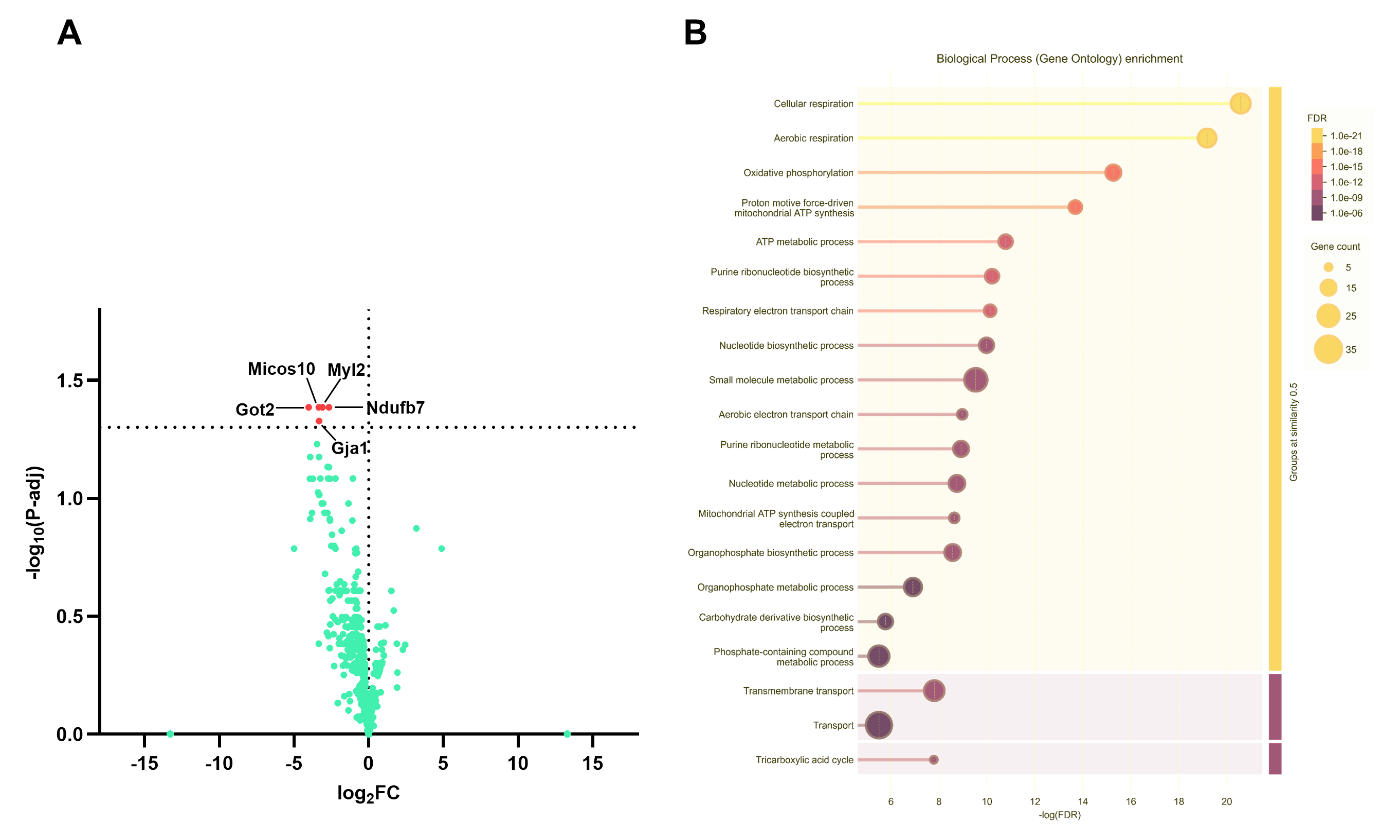
Figure S6. Phosphoproteome analysis of mitochondria isolated from WT and NLRX-1^-/-^ mice.**(**A**) Volcano plot of the fold-change changes in the phosphorylation status of mitochondrial proteins between WT and KO mitochondria (n=3 biological replicates per group), depicted for the adjusted (Benjamini-Hochberg) P-value (− log10 p value). Green points represent unchanged proteins; red spots indicate changed proteins with adjusted *P* value < 0.05. (**B**) Gene Ontology (GO) enrichment analysis of biological processes based on phosphoproteome changes affected by NLRX1 deletion in isolated mitochondria (n=3 WT, n=3 KO).


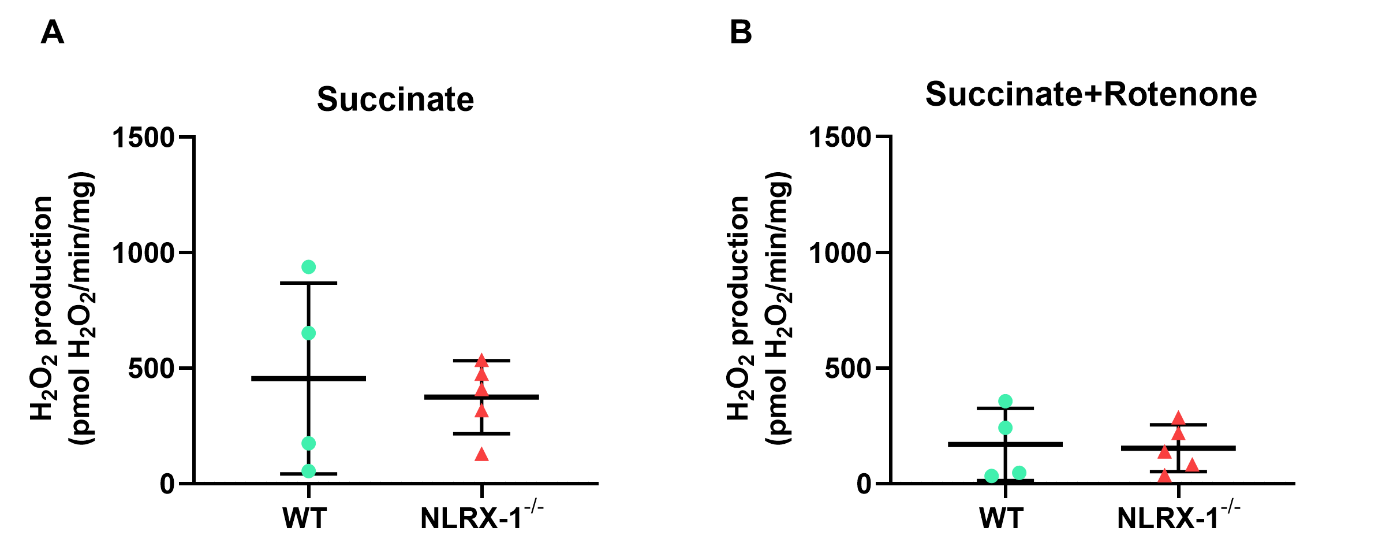
**Figure S7. H_2_O_2_ production of mitochondria isolated from hearts of WT and NLRX-1-/- mice after 35 min ischemia and 7 min reperfusion.** Mitochondrial H_2_O_2_ production was measured by using the Amplex Red reagent (Thermo Fisher). (**A)** H_2_O_2_ production with succinate (5 mM) as substrate, **(B)** H_2_O_2_ production for succinate (5 mM) with the complex 1 inhibitor Rotenone (2 μM), to prevent H_2_O_2_ production through reversed electron transport chain activity ; N=4-5, Data shown are Mean ± SD. Statistical significance was evaluated by non-paired t test**.**


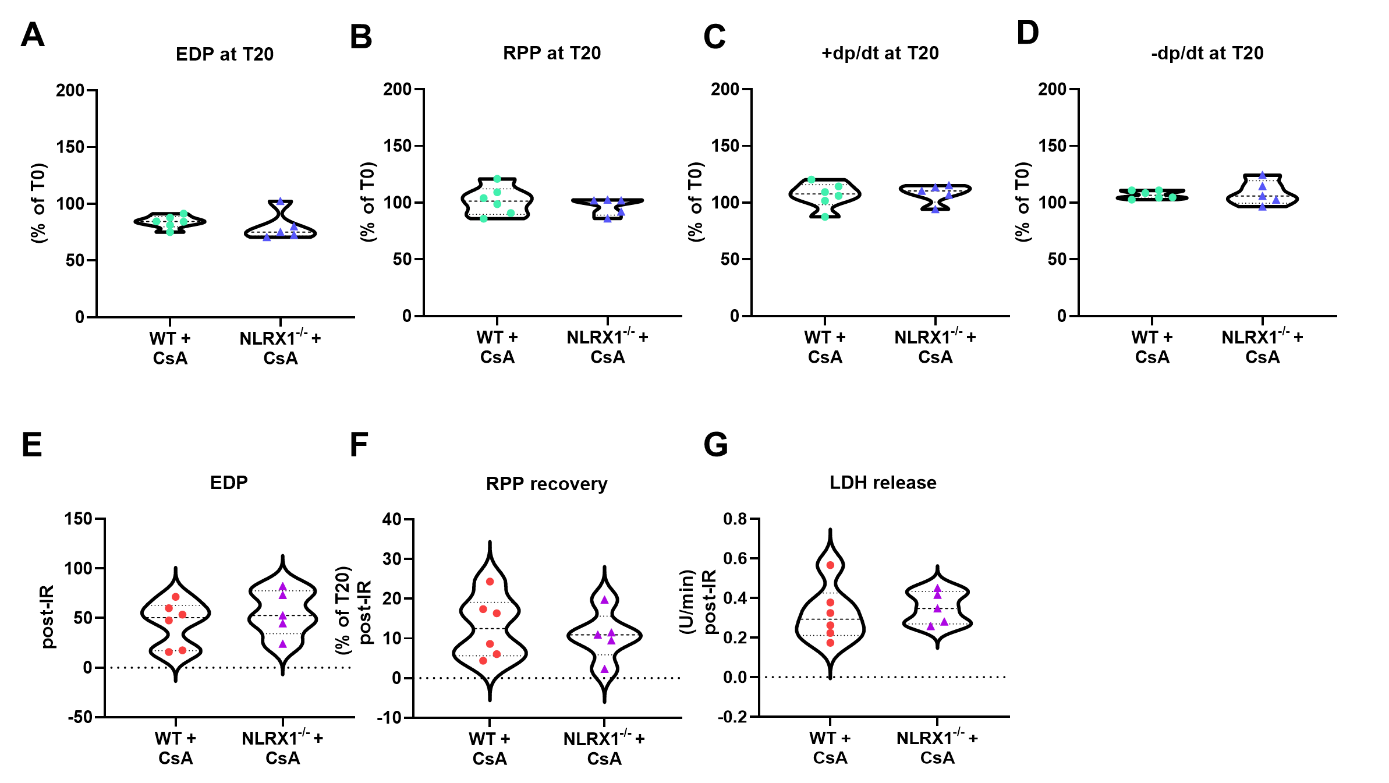


**Figure S8** **Cyclosporin A equalizes IRI between WT and NLRX1^-/-^ mice hearts.** Hearts from WT and NLRX1^-/-^ mice were subjected to IR procedure with cyclosporine A (CsA, mPTP inhibitor) administrated during 20 min baseline and first 60 min reperfusion (n=6 [WT+CsA], 5 [NLRX1-/-+CsA]). **A-D**, For all basal functional parameters, the change at T=20 min relative to the value at T=0 min was depicted. Mechanical function was indicated by end diastolic pressure (**A**, EDP); Rate pressure product (**B**, RPP = DLVP X HR); Maximum contraction rate of left ventricle (**C**, +dp/dt); and Maximum relaxation rate of left ventricle (**D**, -dp/dt). CsA equalized IRI between WT and KO, as indicated by EDP (**E**) at 90 min reperfusion (T=145 min); RPP recovery (**F**, % of RPP at 90 min reperfusion [T=145 min] related to RPP at baseline [T=20 min]); LDH release in coronary effluent (**G**, normalized to coronary flow. Data shown are median ± IQ. Statistical significance was evaluated by nonpaired t test (normally distributed data) or Mann-Whitney test (non-normally distributed data).

**Supplementary Table 1 Baseline characteristics of mice in all series**

|  |  |  | **Urocortin** | | **Cyclosporin A** | |
| --- | --- | --- | --- | --- | --- | --- |
|  | **WT** | **NLRX1^-/-^** | **WT** | **NLRX1^-/-^** | **WT** | **NLRX1^-/-^** |
| **Pperf (mmHg)** | 84±6 | 82±5 | 84±6 | 85±10 | 81±4 | 84±5 |
| **EDP (mmHg)** | 4.1±0.5 | 3.9±0.7 | 4.2±0.7 | 4.1±0.9 | 3.6±0.3 | 4.0±0.4 |
| **DLVP (mmHg)** | 107±19 | 110±17 | 121±25 | 112±15 | 97±17 | 112±24 |
| **HR (b.p.m)** | 344±21 | 348±28 | 342±20 | 320±26 | 376±39 | 339±53 |
| **RPP** | 36631±6120 | 37940±5093 | 41413±8700 | 35948±5478 | 36813±9130 | 38106±10120 |
| **+dp/dt** | 4214±714 | 4359±584 | 4429±841 | 4650±564 | 4138±561 | 4306±900 |
| **-dp/dt** | 3801±676 | 3859±470 | 4429±841 | 4085±537 | 3394±605 | 3613±602 |
| **BW (g)** | 27.1±2.6 | 26.6±2.4 | 26.6±1.5 | 26.3±1.0 | 28.2±1.9 | 26.8±0.7 |
| **Flow (ml/min)** | 1.8±0.3 | 1.9±0.4 | 2.1±0.4 | 2.1±0.4 | 2.1±0.2 | 2.3±0.5 |
| **T (°C)** | 36.7±0.1 | 36.7±0.1 | 36.8±0.1 | 36.8±0.1 | 36.8±0.1 | 36.8±0.1 |
| **HW (mg)** | 29.2±2.9**^¡^** | 29.5±3.0**^¡^** | 131.0±8.4 | 140.8±112 | 156.5±8.0 | 152.2±7.8 |
| **n** | 28 | 28 | 9 | 9 | 6 | 5 |

No differences in baseline parameters were observed between groups within each series across all groups. Pperf (mmHg): perfusion pressure; EDP (mmHg): end diastolic pressure; DLVP (mmHg): developed left ventricular pressure; HR (b.p.m): heart rate; RPP (mmHg*bpm): rate pressure product; +dp/dt (mmHg/s): maximal rate of rise of left ventricular pressure; -dp/dt (mmHg/s): maximal rate of drop of left ventricular pressure; BW (g): body weight; T (°C): temperature; HW (mg): heart weight, **^¡^**dry weight with n= 16,wet weight for Cyclosporin A and Urocortin series; n: sample size. Data shown are mean±SD. Statistical significance was evaluated by nonpaired t test (normally distributed data) and Mann-Whitney test (non-normally distributed data).

**Supplementary Table 2 List of antibodies**

| **Antibody** | **Product nr.** | **Company** |
| --- | --- | --- |
| NLRX1 | ab107611 | Abcam |
| GAPDH | #2118 | Cell Signaling Technology |
| α-Tubulin | T9026 | Sigma |
| VDAC | #4661 | Cell Signaling Technology |
| Cyclophilin D | ab110324 | Abcam |
| ANT1/2 | ab110322 | Abcam |
| ATP F0/F1 | ab14748 | Abcam |
| phospho-AMPKa | #2535 | Cell Signaling Technology |
| AMPKa | #2603 | Cell Signaling Technology |
| OXPHOS Cocktail | ab110413 | Abcam |
| P62 | ab91526 | Abcam |
| LC3 | NB100-2220 | Novusbio |
| LAMP1 | ab24170 | Abcam |
| LAMP2 | ABL-93 | DSHB |
| CTSB | #31718 | Cell Signaling Technology |
| phosphor-mTOR | #5536 | Cell Signaling Technology |
| mTOR | #2983 | Cell Signaling Technology |
| Raptor | #2280 | Cell Signaling Technology |
| Rictor | #2114 | Cell Signaling Technology |
| mLST8 | #3274 | Cell Signaling Technology |
| phospho-Akt | #9271 | Cell Signaling Technology |
| Akt | #9272 | Cell Signaling Technology |
| phospho-ERK1/2 | #9101 | Cell Signaling Technology |
| ERK1/2 | #9102 | Cell Signaling Technology |
| phospho-S6K | #9205 | Cell Signaling Technology |
| S6K | #9202 | Cell Signaling Technology |
| MCU | #14997 | Cell Signaling Technology |
| MICU1 | #12524 | Cell Signaling Technology |
| MICU2 | ab10146 | Abcam |
| SLC24A6 | Sab2102181 | Sigma-Aldrich |
| TOM20 | #42406 | Cell Signaling Technology |
| HSP60 | #12165 | Cell Signaling Technology |
| COXIV | #11967 | Cell Signaling Technology |

**References**

1. Brenner GB, Giricz Z, Garamvolgyi R, Makkos A, Onodi Z, Sayour NV, Gergely TG, Baranyai T, Petnehazy O, Korosi D, Szabo GP, Vago H, Dohy Z, Czimbalmos C, Merkely B, Boldin-Adamsky S, Feinstein E, Horvath IG, Ferdinandy P (2021) Post-Myocardial Infarction Heart Failure in Closed-chest Coronary Occlusion/Reperfusion Model in Gottingen Minipigs and Landrace Pigs. J Vis Expdoi: 10.3791/61901
2. Durgan DJ, Pulinilkunnil T, Villegas-Montoya C, Garvey ME, Frangogiannis NG, Michael LH, Chow CW, Dyck JR, Young ME (2010) Short communication: ischemia/reperfusion tolerance is time-of-day-dependent: mediation by the cardiomyocyte circadian clock. Circ Res 106:546-550. doi: 10.1161/CIRCRESAHA.109.209346
3. Xiao Y, Yim K, Zhang H, Bakker D, Nederlof R, Smeitink JAM, Renkema H, Hollmann MW, Weber NC, Zuurbier CJ (2021) The Redox Modulating Sonlicromanol Active Metabolite KH176m and the Antioxidant MPG Protect Against Short-Duration Cardiac Ischemia-Reperfusion Injury. Cardiovasc Drugs Ther 35:745-758. doi: 10.1007/s10557-021-07189-9
4. Zhang H, Xiao Y, Nederlof R, Bakker D, Zhang P, Girardin SE, Hollmann MW, Weber NC, Houten SM, van Weeghel M, Kibbey RG, Zuurbier CJ (2020) NLRX1 Deletion Increases Ischemia-Reperfusion Damage and Activates Glucose Metabolism in Mouse Heart. Front Immunol 11:591815. doi: 10.3389/fimmu.2020.5918155.
5. Doncheva NT, Pyysalo S, Bork P, Jensen LJ, von Mering C (2023) The STRING database in 2023: protein-protein association networks and functional enrichment analyses for any sequenced genome of interest. Nucleic Acids Res 51(D1):D638-D646. doi: 10.1093/nar/gkac1000
