# Supplementary material for: The innate immune receptor NLRX1 is a novel required modulator for mPTP opening: implications for cardioprotection
Source: Basic Res Cardiol. 2025 Jun 19;120(4):707–25. doi: 10.1007/s00395-025-01124-x (PMC12325489; doi:10.1007/s00395-025-01124-x)
Supplement: Supplementary file 2 — Supplementary file2 (PPTX 82440 KB) [file 395_2025_1124_MOESM2_ESM.pptx]

## Slide 1
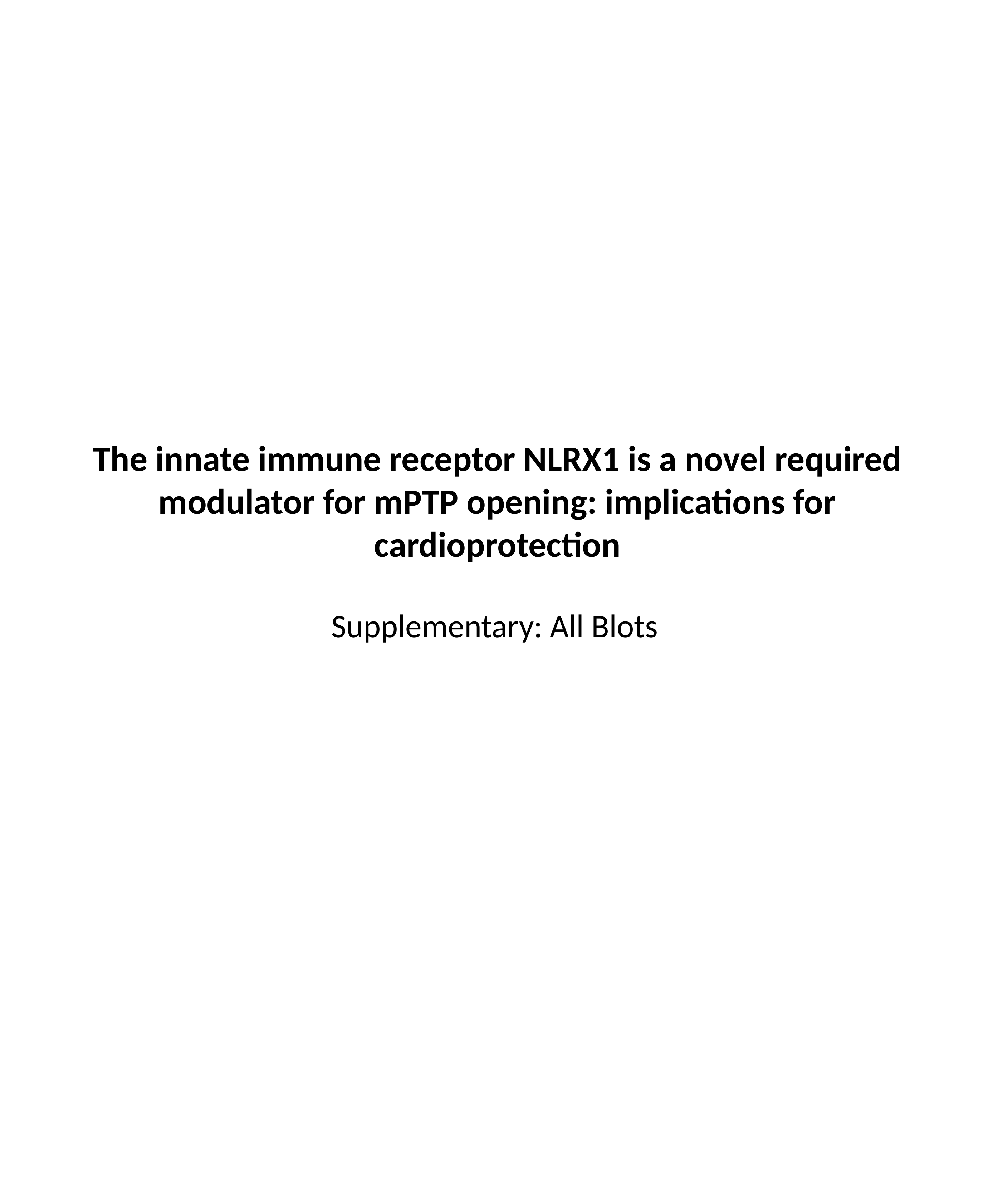

The innate immune receptor NLRX1 is a novel required modulator for mPTP opening: implications for cardioprotection
Supplementary: All Blots

## Slide 2
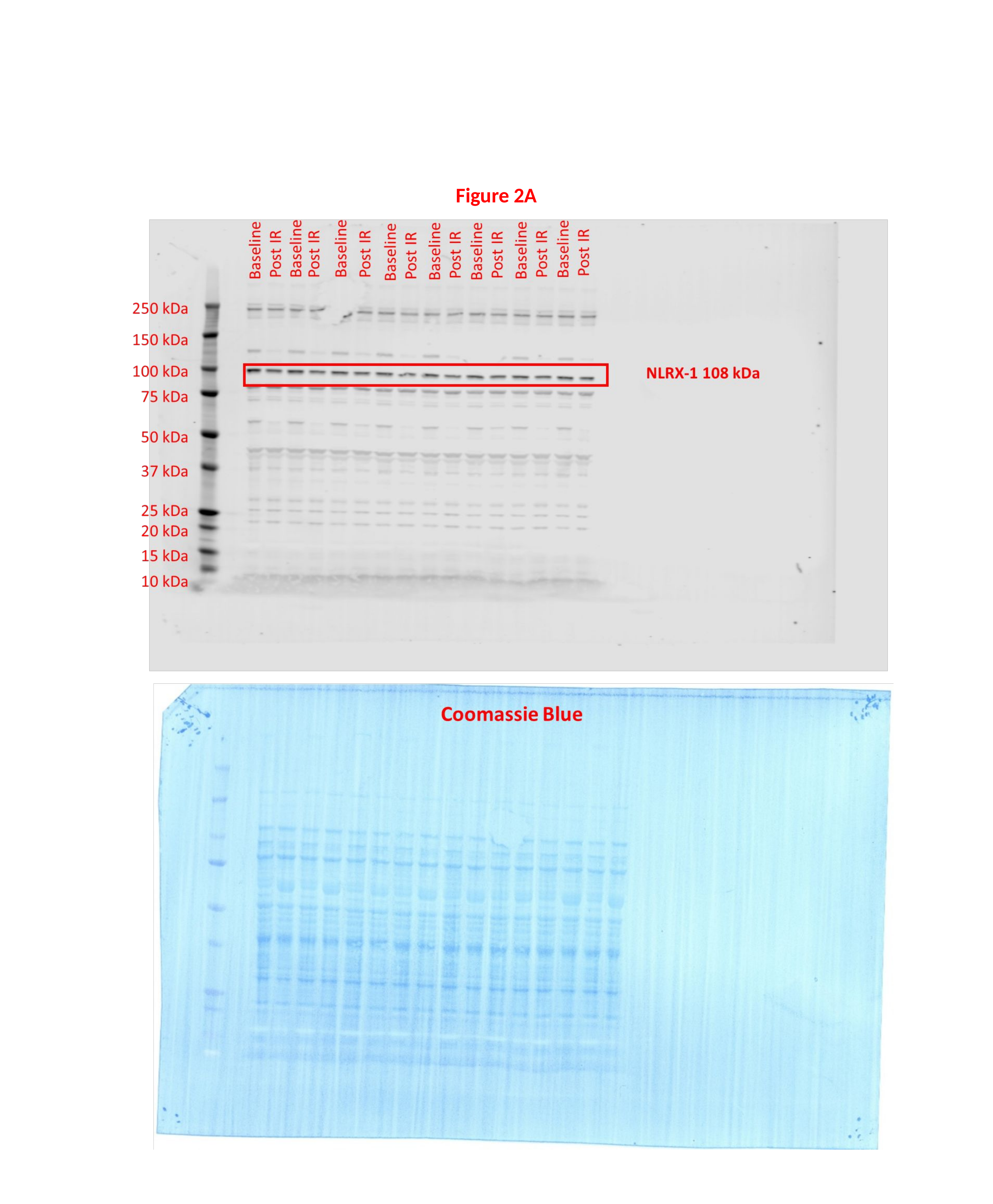

Figure 2A

## Slide 3
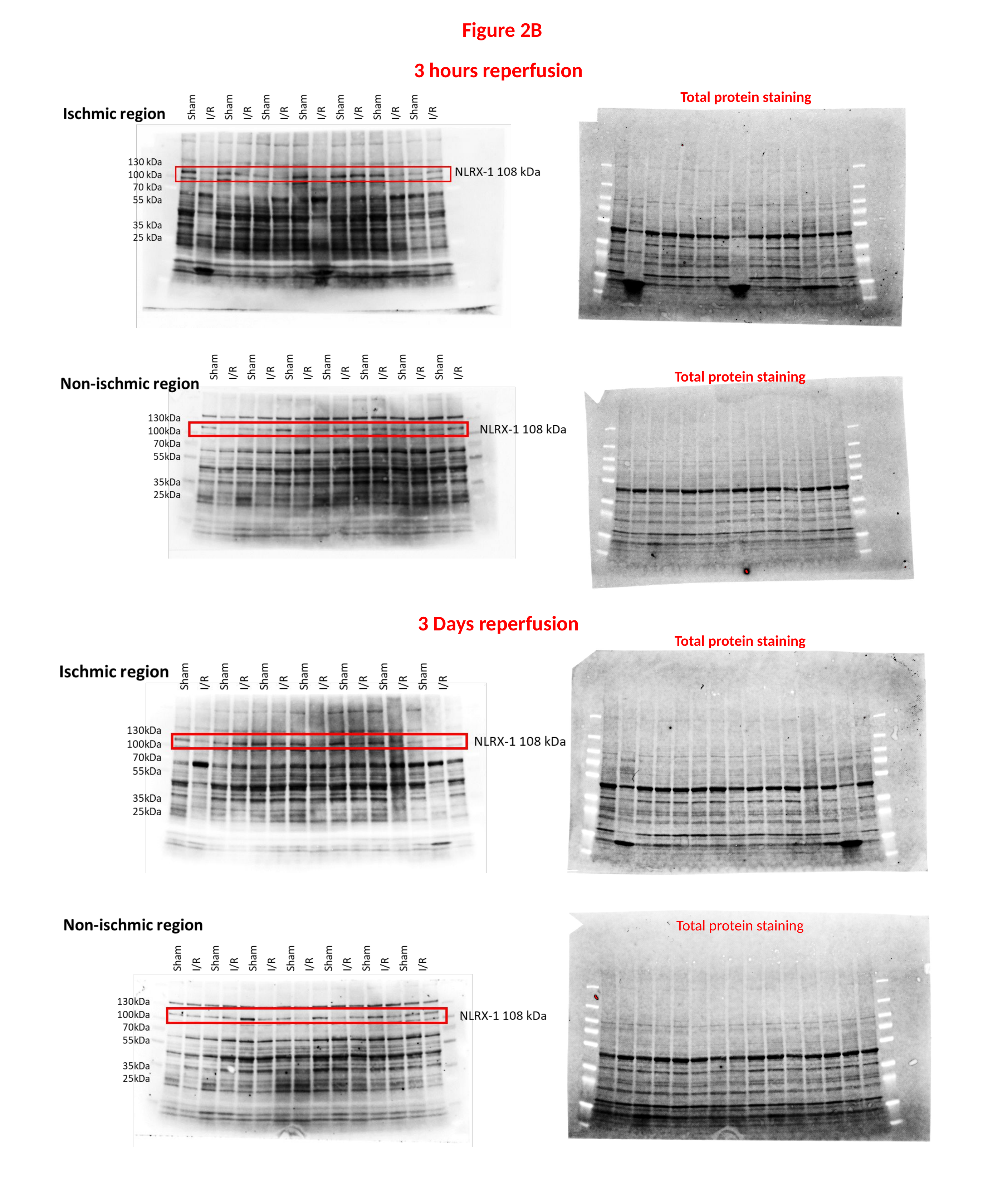

Figure 2B
3 hours reperfusion
Total protein staining
Total protein staining
3 Days reperfusion
Total protein staining
Total protein staining

## Slide 4
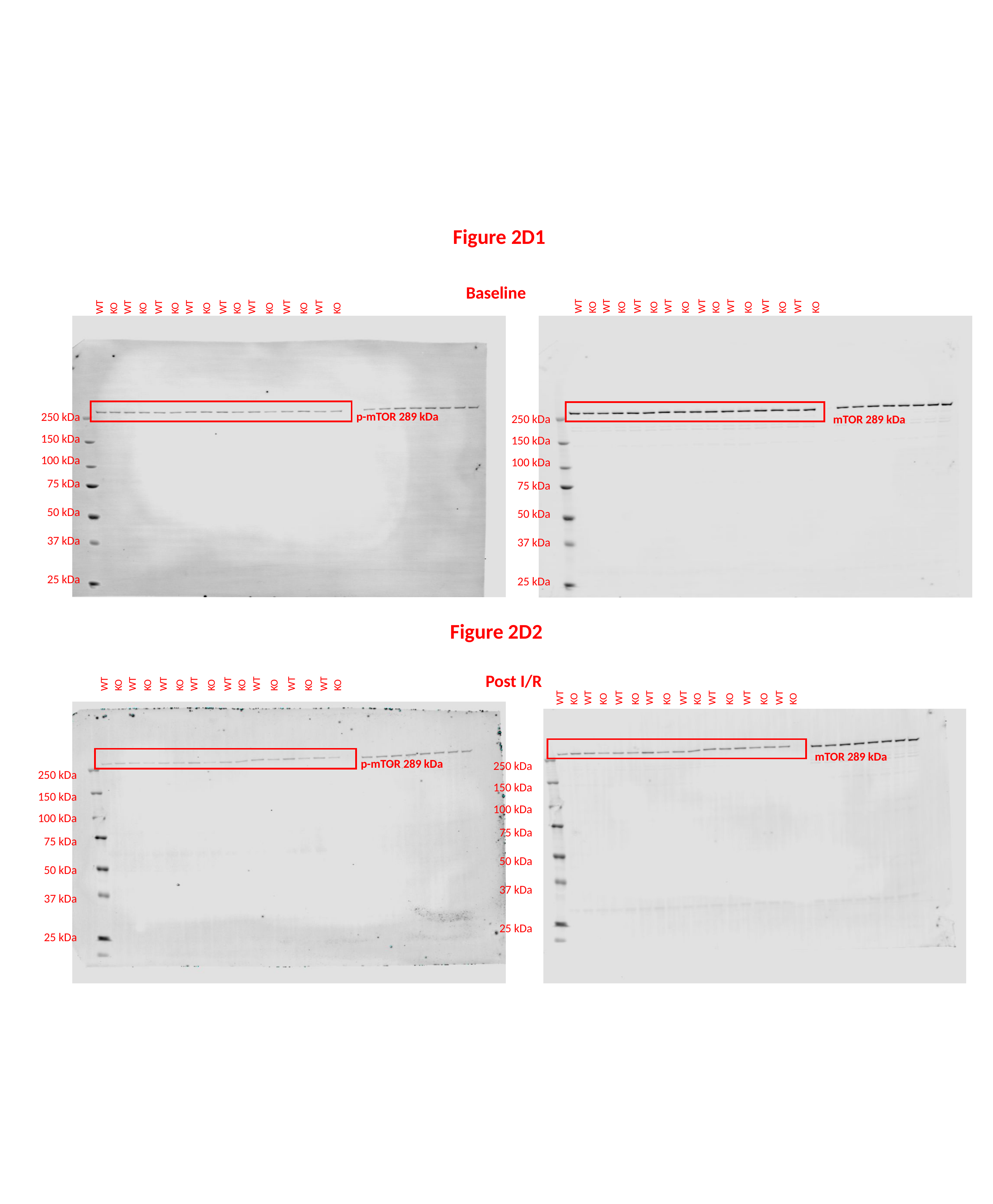

Figure 2D1
WT
WT
WT
WT
WT
WT
WT
WT
KO
KO
KO
KO
KO
KO
KO
KO
WT
WT
WT
WT
WT
WT
WT
WT
KO
KO
KO
KO
KO
KO
KO
KO
Baseline
p-mTOR 289 kDa
250 kDa
150 kDa
100 kDa
75 kDa
50 kDa
37 kDa
25 kDa
250 kDa
150 kDa
100 kDa
75 kDa
50 kDa
37 kDa
25 kDa
mTOR 289 kDa
WT
WT
WT
WT
WT
WT
WT
WT
KO
KO
KO
KO
KO
KO
KO
KO
WT
WT
WT
WT
WT
WT
WT
WT
KO
KO
KO
KO
KO
KO
KO
KO
Post I/R
mTOR 289 kDa
p-mTOR 289 kDa
250 kDa
150 kDa
100 kDa
75 kDa
50 kDa
37 kDa
25 kDa
250 kDa
150 kDa
100 kDa
75 kDa
50 kDa
37 kDa
25 kDa
Figure 2D2

## Slide 5
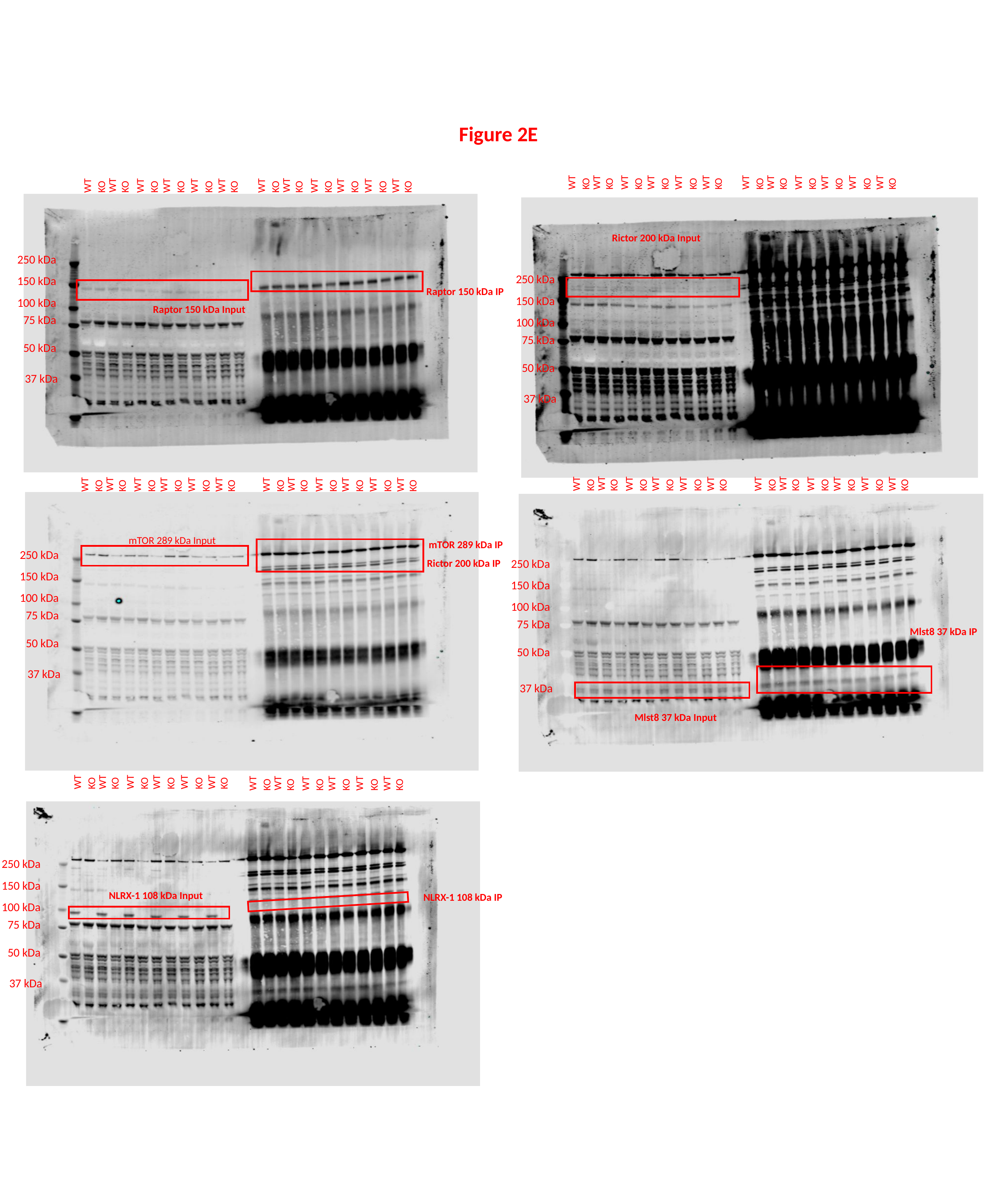

Figure 2E
WT
WT
WT
WT
WT
WT
KO
KO
KO
KO
KO
KO
WT
WT
WT
WT
WT
WT
KO
KO
KO
KO
KO
KO
WT
WT
WT
WT
WT
WT
KO
KO
KO
KO
KO
KO
WT
WT
WT
WT
WT
WT
KO
KO
KO
KO
KO
KO
Rictor 200 kDa Input
250 kDa
150 kDa
100 kDa
75 kDa
50 kDa
37 kDa
250 kDa
150 kDa
100 kDa
75 kDa
50 kDa
37 kDa
Raptor 150 kDa IP
Raptor 150 kDa Input
WT
WT
WT
WT
WT
WT
KO
KO
KO
KO
KO
KO
WT
WT
WT
WT
WT
WT
KO
KO
KO
KO
KO
KO
WT
WT
WT
WT
WT
WT
KO
KO
KO
KO
KO
KO
WT
WT
WT
WT
WT
WT
KO
KO
KO
KO
KO
KO
mTOR 289 kDa Input
mTOR 289 kDa IP
250 kDa
150 kDa
100 kDa
75 kDa
50 kDa
37 kDa
250 kDa
150 kDa
100 kDa
75 kDa
50 kDa
37 kDa
Rictor 200 kDa IP
Mlst8 37 kDa IP
Mlst8 37 kDa Input
WT
WT
WT
WT
WT
WT
KO
KO
KO
KO
KO
KO
WT
WT
WT
WT
WT
WT
KO
KO
KO
KO
KO
KO
250 kDa
150 kDa
100 kDa
75 kDa
50 kDa
37 kDa
NLRX-1 108 kDa Input
NLRX-1 108 kDa IP

## Slide 6
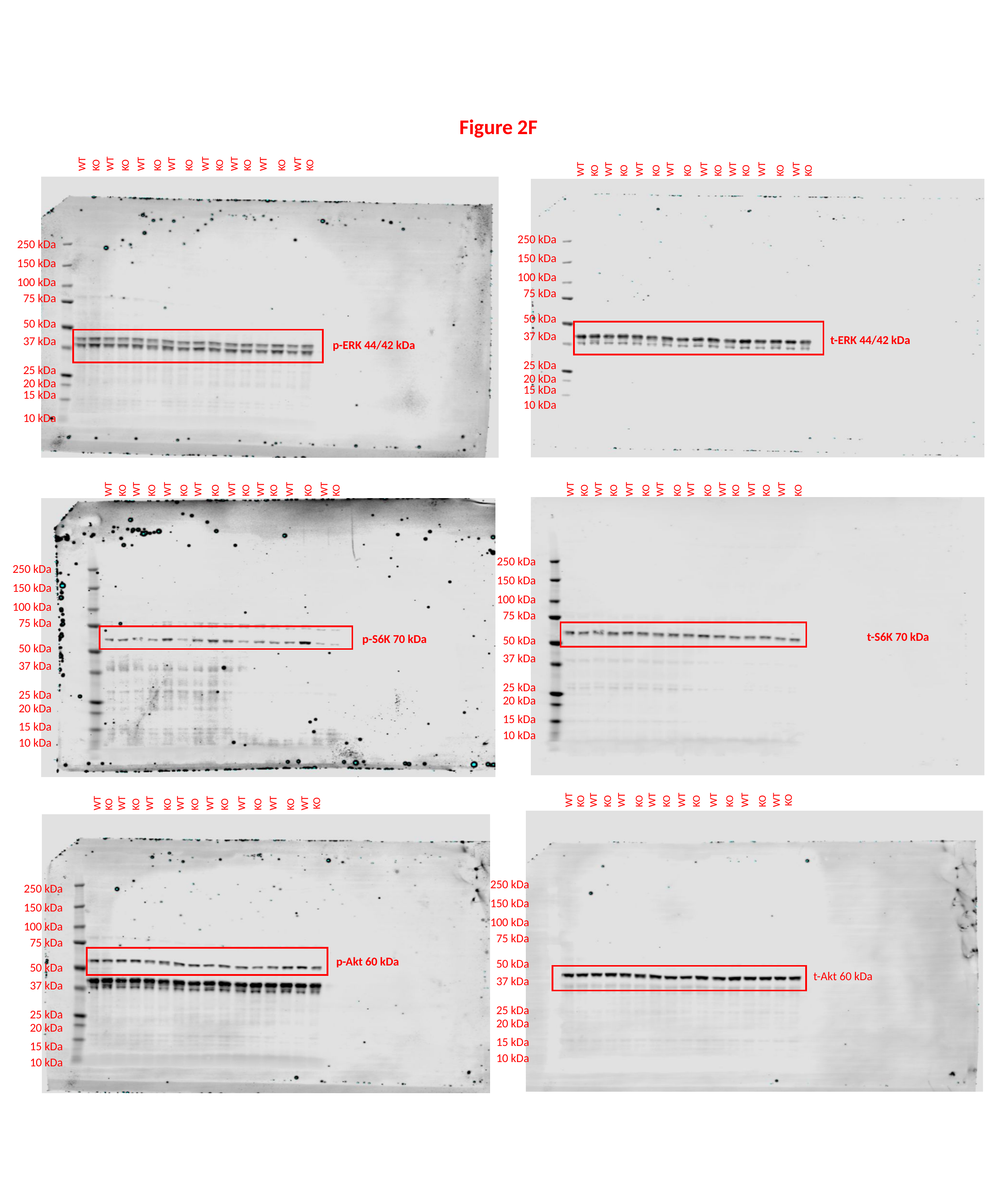

Figure 2F
WT
WT
WT
WT
WT
WT
WT
WT
KO
KO
KO
KO
KO
KO
KO
KO
WT
WT
WT
WT
WT
WT
WT
WT
KO
KO
KO
KO
KO
KO
KO
KO
250 kDa
150 kDa
100 kDa
75 kDa
50 kDa
37 kDa
25 kDa
20 kDa
15 kDa
10 kDa
250 kDa
150 kDa
100 kDa
75 kDa
50 kDa
37 kDa
25 kDa
20 kDa
15 kDa
10 kDa
t-ERK 44/42 kDa
p-ERK 44/42 kDa
WT
WT
WT
WT
WT
WT
WT
WT
KO
KO
KO
KO
KO
KO
KO
KO
WT
WT
WT
WT
WT
WT
WT
WT
KO
KO
KO
KO
KO
KO
KO
KO
250 kDa
150 kDa
100 kDa
75 kDa
50 kDa
37 kDa
25 kDa
20 kDa
15 kDa
10 kDa
250 kDa
150 kDa
100 kDa
75 kDa
50 kDa
37 kDa
25 kDa
20 kDa
15 kDa
10 kDa
	t-S6K 70 kDa
p-S6K 70 kDa
WT
WT
WT
WT
WT
WT
WT
WT
KO
KO
KO
KO
KO
KO
KO
KO
WT
WT
WT
WT
WT
WT
WT
WT
KO
KO
KO
KO
KO
KO
KO
KO
250 kDa
150 kDa
100 kDa
75 kDa
50 kDa
37 kDa
25 kDa
20 kDa
15 kDa
10 kDa
250 kDa
150 kDa
100 kDa
75 kDa
50 kDa
37 kDa
25 kDa
20 kDa
15 kDa
10 kDa
p-Akt 60 kDa
t-Akt 60 kDa

## Slide 7
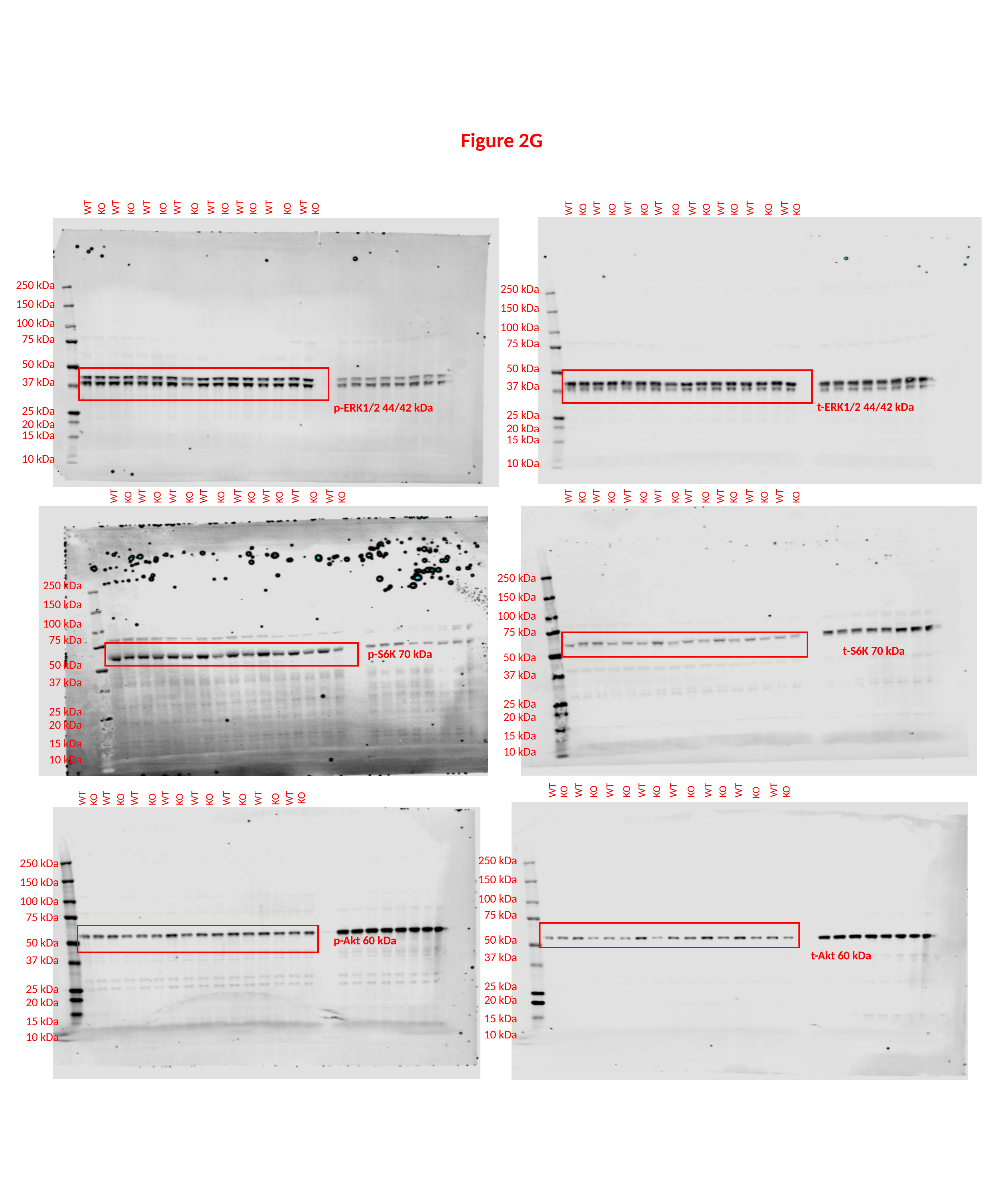

Figure 2G
WT
WT
WT
WT
WT
WT
WT
WT
KO
KO
KO
KO
KO
KO
KO
KO
WT
WT
WT
WT
WT
WT
WT
WT
KO
KO
KO
KO
KO
KO
KO
KO
250 kDa
150 kDa
100 kDa
75 kDa
50 kDa
37 kDa
25 kDa
20 kDa
15 kDa
10 kDa
250 kDa
150 kDa
100 kDa
75 kDa
50 kDa
37 kDa
25 kDa
20 kDa
15 kDa
10 kDa
t-ERK1/2 44/42 kDa
p-ERK1/2 44/42 kDa
WT
WT
WT
WT
WT
WT
WT
WT
KO
KO
KO
KO
KO
KO
KO
KO
WT
WT
WT
WT
WT
WT
WT
WT
KO
KO
KO
KO
KO
KO
KO
KO
250 kDa
150 kDa
100 kDa
75 kDa
50 kDa
37 kDa
25 kDa
20 kDa
15 kDa
10 kDa
250 kDa
150 kDa
100 kDa
75 kDa
50 kDa
37 kDa
25 kDa
20 kDa
15 kDa
10 kDa
	t-S6K 70 kDa
p-S6K 70 kDa
WT
WT
WT
WT
WT
WT
WT
WT
KO
KO
KO
KO
KO
KO
KO
KO
WT
WT
WT
WT
WT
WT
WT
WT
KO
KO
KO
KO
KO
KO
KO
KO
250 kDa
150 kDa
100 kDa
75 kDa
50 kDa
37 kDa
25 kDa
20 kDa
15 kDa
10 kDa
250 kDa
150 kDa
100 kDa
75 kDa
50 kDa
37 kDa
25 kDa
20 kDa
15 kDa
10 kDa
p-Akt 60 kDa
t-Akt 60 kDa

## Slide 8
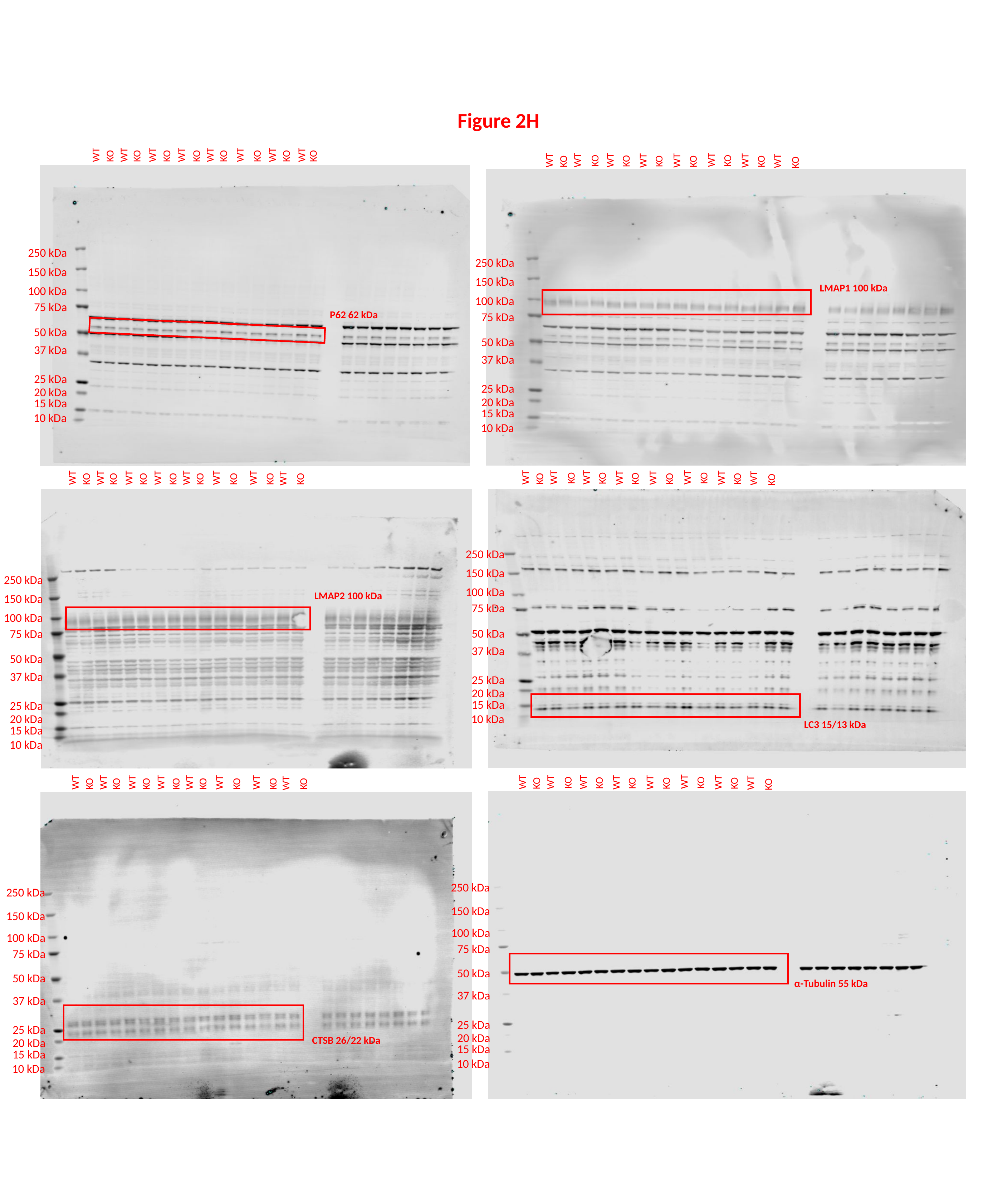

Figure 2H
WT
WT
WT
WT
WT
WT
WT
WT
KO
KO
KO
KO
KO
KO
KO
KO
WT
WT
WT
WT
WT
WT
WT
WT
KO
KO
KO
KO
KO
KO
KO
KO
250 kDa
250 kDa
150 kDa
100 kDa
75 kDa
50 kDa
37 kDa
25 kDa
20 kDa
15 kDa
10 kDa
150 kDa
LMAP1 100 kDa
100 kDa
75 kDa
P62 62 kDa
50 kDa
37 kDa
25 kDa
20 kDa
15 kDa
10 kDa
WT
WT
WT
WT
WT
WT
WT
WT
KO
KO
KO
KO
KO
KO
KO
KO
WT
WT
WT
WT
WT
WT
WT
WT
KO
KO
KO
KO
KO
KO
KO
KO
250 kDa
150 kDa
100 kDa
75 kDa
50 kDa
37 kDa
25 kDa
20 kDa
15 kDa
10 kDa
250 kDa
150 kDa
100 kDa
75 kDa
50 kDa
37 kDa
25 kDa
20 kDa
15 kDa
10 kDa
LMAP2 100 kDa
LC3 15/13 kDa
WT
WT
WT
WT
WT
WT
WT
WT
KO
KO
KO
KO
KO
KO
KO
KO
WT
WT
WT
WT
WT
WT
WT
WT
KO
KO
KO
KO
KO
KO
KO
KO
250 kDa
150 kDa
100 kDa
75 kDa
50 kDa
37 kDa
25 kDa
20 kDa
15 kDa
10 kDa
250 kDa
150 kDa
100 kDa
75 kDa
50 kDa
37 kDa
25 kDa
20 kDa
15 kDa
10 kDa
α-Tubulin 55 kDa
CTSB 26/22 kDa

## Slide 9
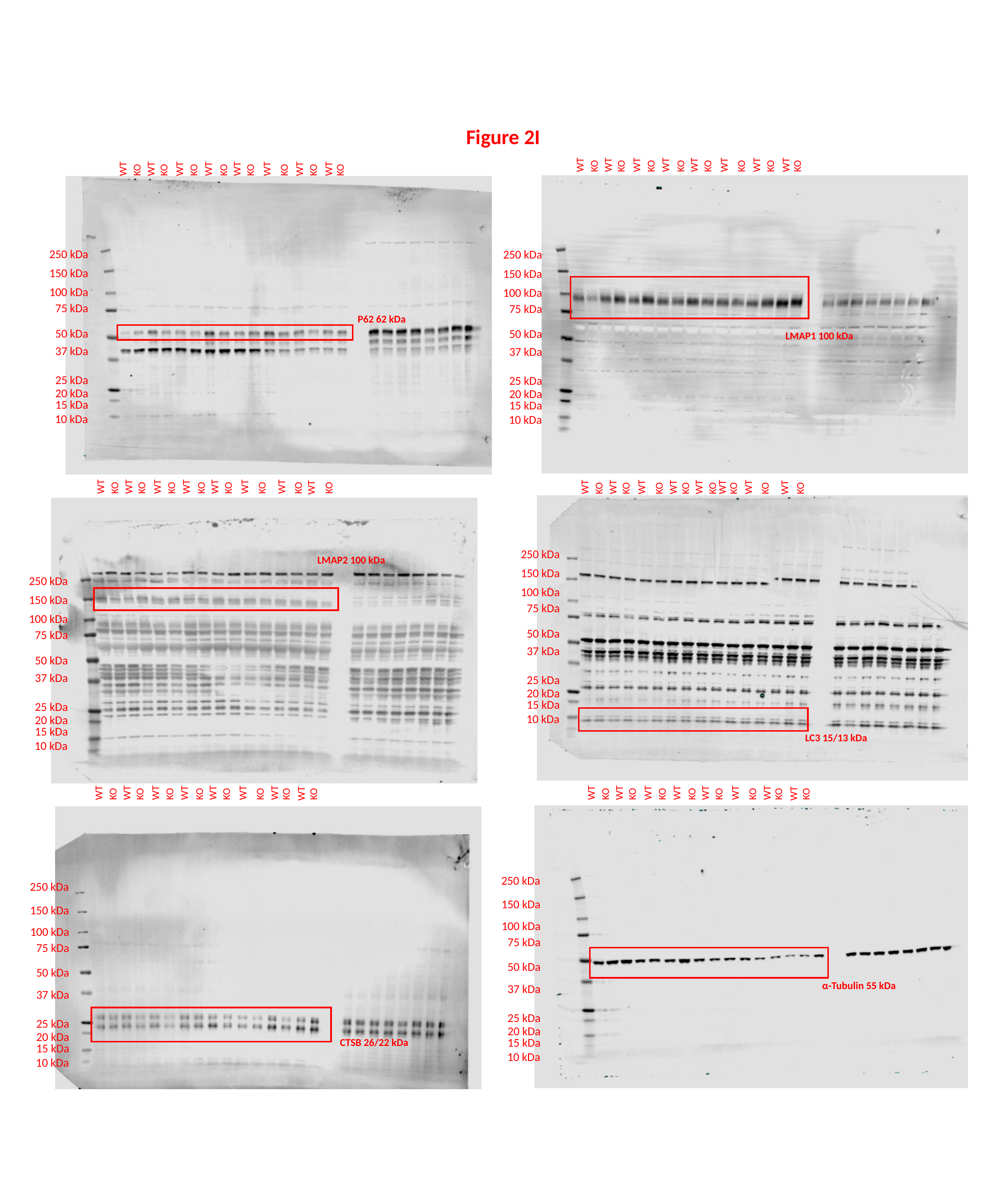

WT
WT
WT
WT
WT
WT
WT
WT
KO
KO
KO
KO
KO
KO
KO
KO
WT
WT
WT
WT
WT
WT
WT
WT
KO
KO
KO
KO
KO
KO
KO
KO
Figure 2I
250 kDa
150 kDa
100 kDa
75 kDa
50 kDa
37 kDa
25 kDa
20 kDa
15 kDa
10 kDa
250 kDa
150 kDa
100 kDa
75 kDa
50 kDa
37 kDa
25 kDa
20 kDa
15 kDa
10 kDa
P62 62 kDa
LMAP1 100 kDa
WT
WT
WT
WT
WT
WT
WT
WT
KO
KO
KO
KO
KO
KO
KO
KO
WT
WT
WT
WT
WT
WT
WT
WT
KO
KO
KO
KO
KO
KO
KO
KO
250 kDa
150 kDa
100 kDa
75 kDa
50 kDa
37 kDa
25 kDa
20 kDa
15 kDa
10 kDa
LMAP2 100 kDa
250 kDa
150 kDa
100 kDa
75 kDa
50 kDa
37 kDa
25 kDa
20 kDa
15 kDa
10 kDa
LC3 15/13 kDa
WT
WT
WT
WT
WT
WT
WT
WT
KO
KO
KO
KO
KO
KO
KO
KO
WT
WT
WT
WT
WT
WT
WT
WT
KO
KO
KO
KO
KO
KO
KO
KO
250 kDa
150 kDa
100 kDa
75 kDa
50 kDa
37 kDa
25 kDa
20 kDa
15 kDa
10 kDa
250 kDa
150 kDa
100 kDa
75 kDa
50 kDa
37 kDa
25 kDa
20 kDa
15 kDa
10 kDa
α-Tubulin 55 kDa
CTSB 26/22 kDa

## Slide 10
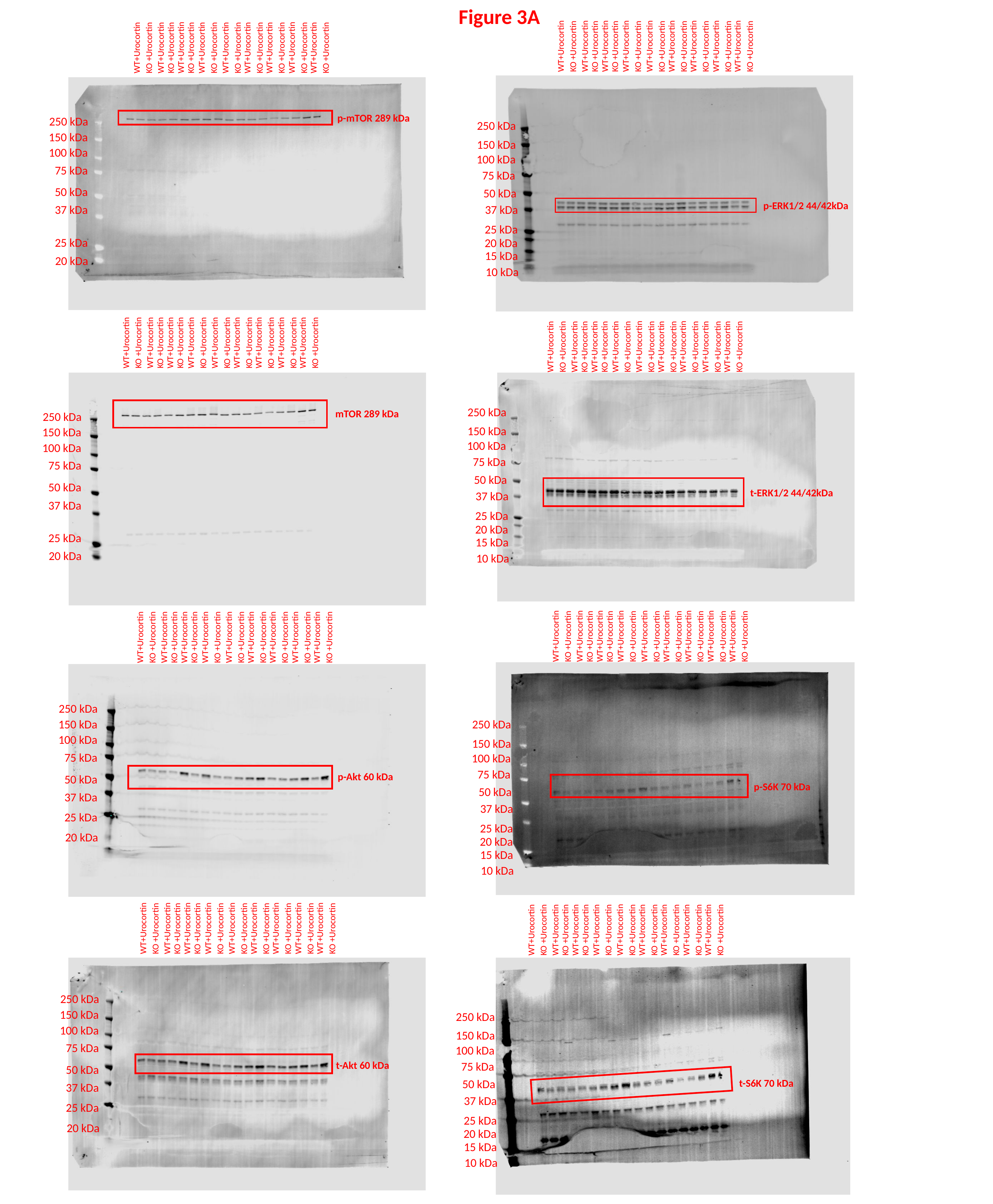

Figure 3A
WT+Urocortin
KO +Urocortin
KO +Urocortin
KO +Urocortin
KO +Urocortin
KO +Urocortin
KO +Urocortin
KO +Urocortin
KO +Urocortin
KO +Urocortin
WT+Urocortin
WT+Urocortin
WT+Urocortin
WT+Urocortin
WT+Urocortin
WT+Urocortin
WT+Urocortin
WT+Urocortin
WT+Urocortin
KO +Urocortin
KO +Urocortin
KO +Urocortin
KO +Urocortin
KO +Urocortin
KO +Urocortin
KO +Urocortin
KO +Urocortin
KO +Urocortin
WT+Urocortin
WT+Urocortin
WT+Urocortin
WT+Urocortin
WT+Urocortin
WT+Urocortin
WT+Urocortin
WT+Urocortin
p-mTOR 289 kDa
250 kDa
150 kDa
100 kDa
75 kDa
50 kDa
37 kDa
25 kDa
20 kDa
250 kDa
150 kDa
100 kDa
75 kDa
50 kDa
37 kDa
25 kDa
20 kDa
15 kDa
10 kDa
p-ERK1/2 44/42kDa
WT+Urocortin
KO +Urocortin
KO +Urocortin
KO +Urocortin
KO +Urocortin
KO +Urocortin
KO +Urocortin
KO +Urocortin
KO +Urocortin
KO +Urocortin
WT+Urocortin
WT+Urocortin
WT+Urocortin
WT+Urocortin
WT+Urocortin
WT+Urocortin
WT+Urocortin
WT+Urocortin
WT+Urocortin
KO +Urocortin
KO +Urocortin
KO +Urocortin
KO +Urocortin
KO +Urocortin
KO +Urocortin
KO +Urocortin
KO +Urocortin
KO +Urocortin
WT+Urocortin
WT+Urocortin
WT+Urocortin
WT+Urocortin
WT+Urocortin
WT+Urocortin
WT+Urocortin
WT+Urocortin
250 kDa
150 kDa
100 kDa
75 kDa
50 kDa
37 kDa
25 kDa
20 kDa
15 kDa
10 kDa
mTOR 289 kDa
250 kDa
150 kDa
100 kDa
75 kDa
50 kDa
37 kDa
25 kDa
20 kDa
t-ERK1/2 44/42kDa
WT+Urocortin
KO +Urocortin
KO +Urocortin
KO +Urocortin
KO +Urocortin
KO +Urocortin
KO +Urocortin
KO +Urocortin
KO +Urocortin
KO +Urocortin
WT+Urocortin
WT+Urocortin
WT+Urocortin
WT+Urocortin
WT+Urocortin
WT+Urocortin
WT+Urocortin
WT+Urocortin
WT+Urocortin
KO +Urocortin
KO +Urocortin
KO +Urocortin
KO +Urocortin
KO +Urocortin
KO +Urocortin
KO +Urocortin
KO +Urocortin
KO +Urocortin
WT+Urocortin
WT+Urocortin
WT+Urocortin
WT+Urocortin
WT+Urocortin
WT+Urocortin
WT+Urocortin
WT+Urocortin
250 kDa
150 kDa
100 kDa
75 kDa
50 kDa
37 kDa
25 kDa
20 kDa
250 kDa
150 kDa
100 kDa
75 kDa
50 kDa
37 kDa
25 kDa
20 kDa
15 kDa
10 kDa
p-Akt 60 kDa
p-S6K 70 kDa
t-Akt
WT+Urocortin
KO +Urocortin
KO +Urocortin
KO +Urocortin
KO +Urocortin
KO +Urocortin
KO +Urocortin
KO +Urocortin
KO +Urocortin
KO +Urocortin
WT+Urocortin
WT+Urocortin
WT+Urocortin
WT+Urocortin
WT+Urocortin
WT+Urocortin
WT+Urocortin
WT+Urocortin
WT+Urocortin
KO +Urocortin
KO +Urocortin
KO +Urocortin
KO +Urocortin
KO +Urocortin
KO +Urocortin
KO +Urocortin
KO +Urocortin
KO +Urocortin
WT+Urocortin
WT+Urocortin
WT+Urocortin
WT+Urocortin
WT+Urocortin
WT+Urocortin
WT+Urocortin
WT+Urocortin
250 kDa
150 kDa
100 kDa
75 kDa
50 kDa
37 kDa
25 kDa
20 kDa
250 kDa
150 kDa
100 kDa
75 kDa
50 kDa
37 kDa
25 kDa
20 kDa
15 kDa
10 kDa
t-Akt 60 kDa
t-S6K 70 kDa

## Slide 11
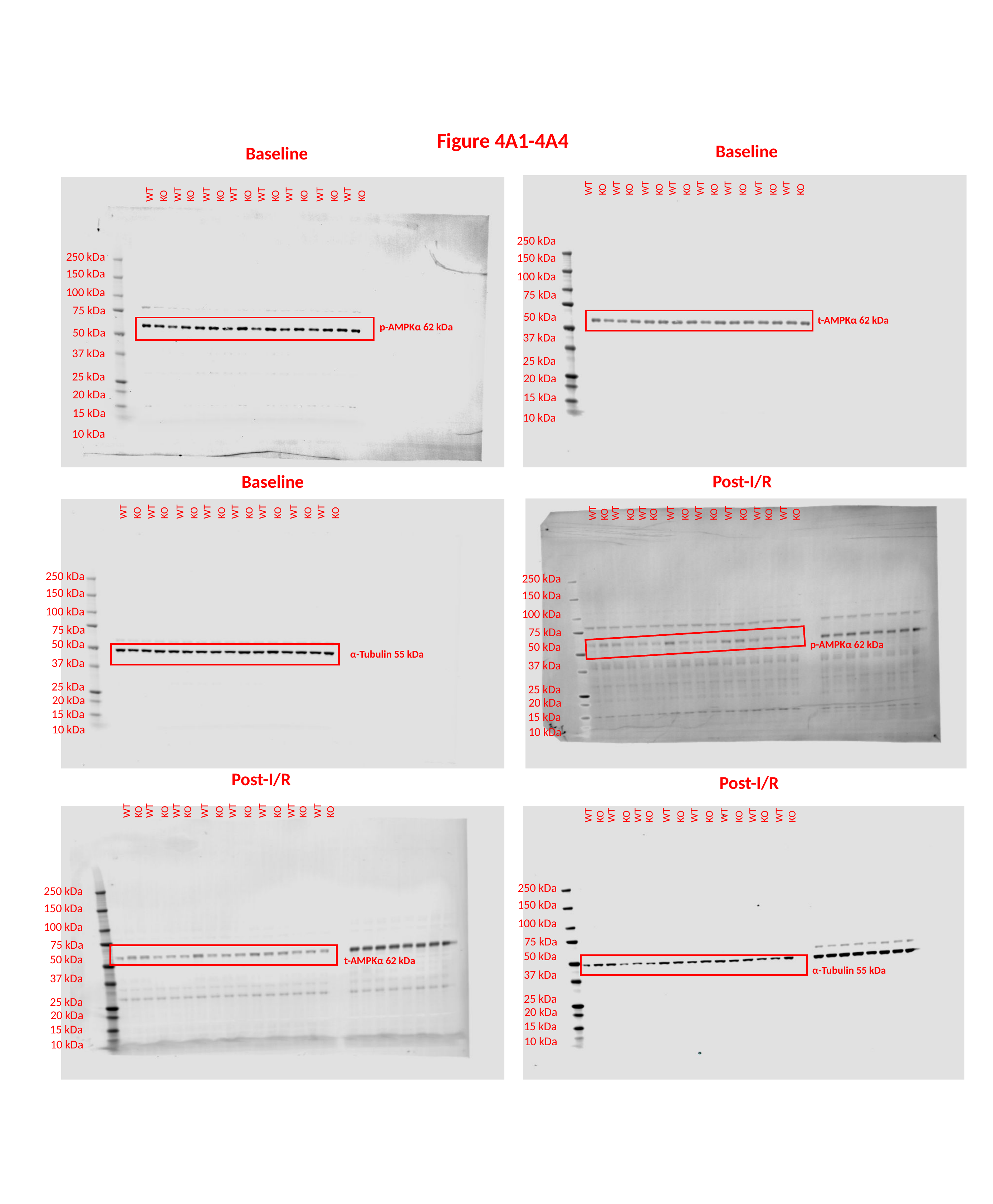

Figure 4A1-4A4
Baseline
WT
WT
WT
WT
WT
WT
WT
WT
KO
KO
KO
KO
KO
KO
KO
KO
Baseline
WT
WT
WT
WT
WT
WT
WT
WT
KO
KO
KO
KO
KO
KO
KO
KO
250 kDa
150 kDa
100 kDa
75 kDa
50 kDa
37 kDa
25 kDa
20 kDa
15 kDa
10 kDa
250 kDa
150 kDa
100 kDa
75 kDa
50 kDa
37 kDa
25 kDa
20 kDa
15 kDa
10 kDa
t-AMPKα 62 kDa
p-AMPKα 62 kDa
WT
WT
WT
WT
WT
WT
WT
WT
KO
KO
KO
KO
KO
KO
KO
KO
WT
WT
WT
WT
WT
WT
WT
WT
KO
KO
KO
KO
KO
KO
KO
KO
Post-I/R
Baseline
250 kDa
150 kDa
100 kDa
75 kDa
50 kDa
37 kDa
25 kDa
20 kDa
15 kDa
10 kDa
250 kDa
150 kDa
100 kDa
75 kDa
50 kDa
37 kDa
25 kDa
20 kDa
15 kDa
10 kDa
p-AMPKα 62 kDa
α-Tubulin 55 kDa
WT
WT
WT
WT
WT
WT
WT
WT
KO
KO
KO
KO
KO
KO
KO
KO
Post-I/R
WT
WT
WT
WT
WT
WT
WT
WT
KO
KO
KO
KO
KO
KO
KO
KO
Post-I/R
250 kDa
150 kDa
100 kDa
75 kDa
50 kDa
37 kDa
25 kDa
20 kDa
15 kDa
10 kDa
250 kDa
150 kDa
100 kDa
75 kDa
50 kDa
37 kDa
25 kDa
20 kDa
15 kDa
10 kDa
t-AMPKα 62 kDa
α-Tubulin 55 kDa

## Slide 12
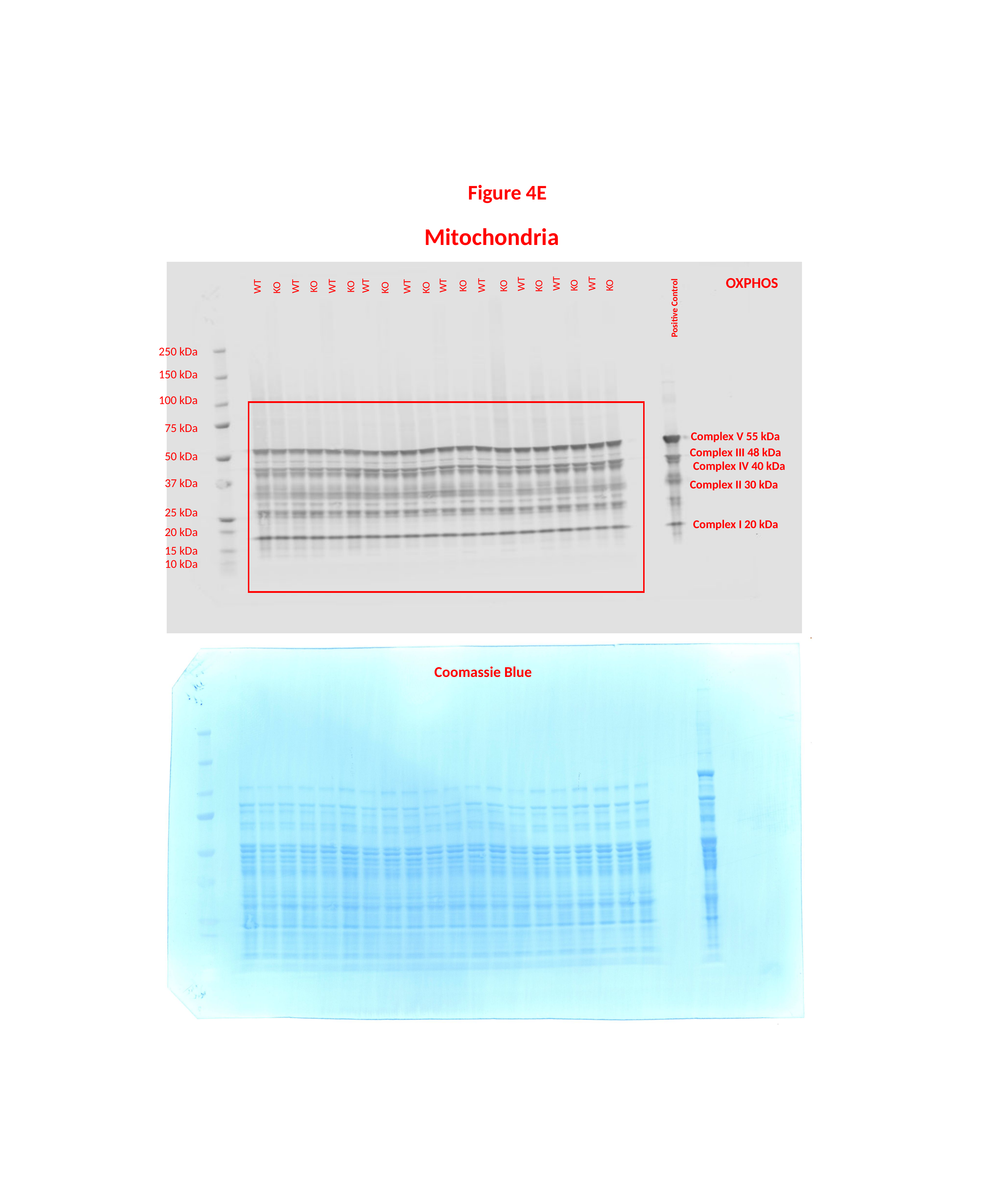

Figure 4E
Mitochondria
WT
WT
WT
WT
WT
WT
WT
WT
KO
KO
KO
KO
KO
KO
KO
KO
WT
WT
KO
KO
OXPHOS
Positive Control
250 kDa
150 kDa
100 kDa
75 kDa
50 kDa
37 kDa
25 kDa
20 kDa
15 kDa
10 kDa
Complex V 55 kDa
Complex III 48 kDa
Complex IV 40 kDa
Complex II 30 kDa
Complex I 20 kDa
Coomassie Blue

## Slide 13
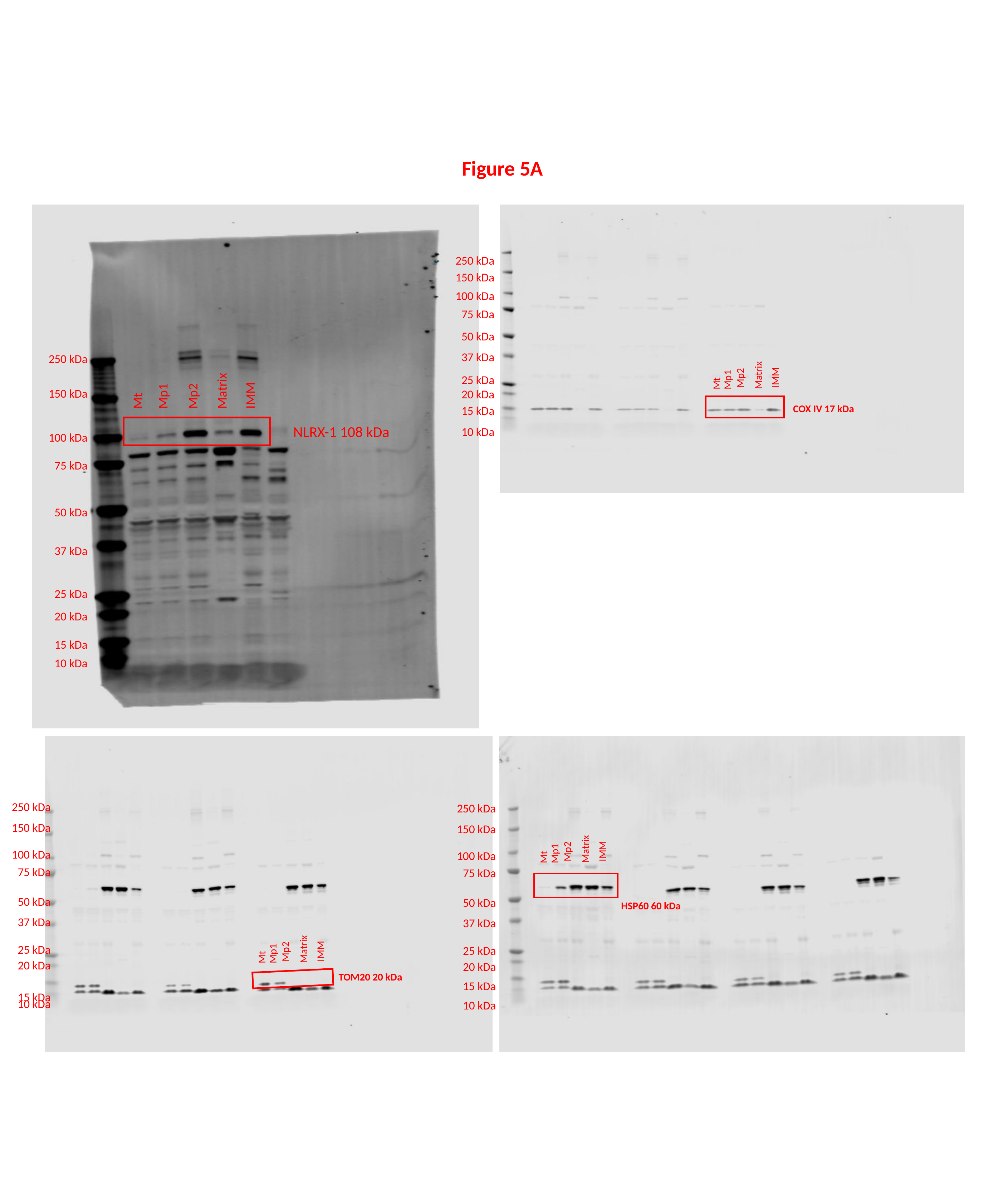

Figure 5A
250 kDa
150 kDa
100 kDa
75 kDa
50 kDa
37 kDa
25 kDa
20 kDa
15 kDa
10 kDa
IMM
Mp2
Matrix
Mt
Mp1
250 kDa
150 kDa
100 kDa
75 kDa
50 kDa
37 kDa
25 kDa
20 kDa
15 kDa
10 kDa
Mt
Mp1
Mp2
Matrix
IMM
COX IV 17 kDa
NLRX-1 108 kDa
250 kDa
150 kDa
100 kDa
75 kDa
50 kDa
37 kDa
25 kDa
20 kDa
15 kDa
10 kDa
250 kDa
150 kDa
100 kDa
75 kDa
50 kDa
37 kDa
25 kDa
20 kDa
15 kDa
10 kDa
IMM
Mp2
Matrix
Mt
Mp1
HSP60 60 kDa
IMM
Mp2
Matrix
Mt
Mp1
TOM20 20 kDa

## Slide 14
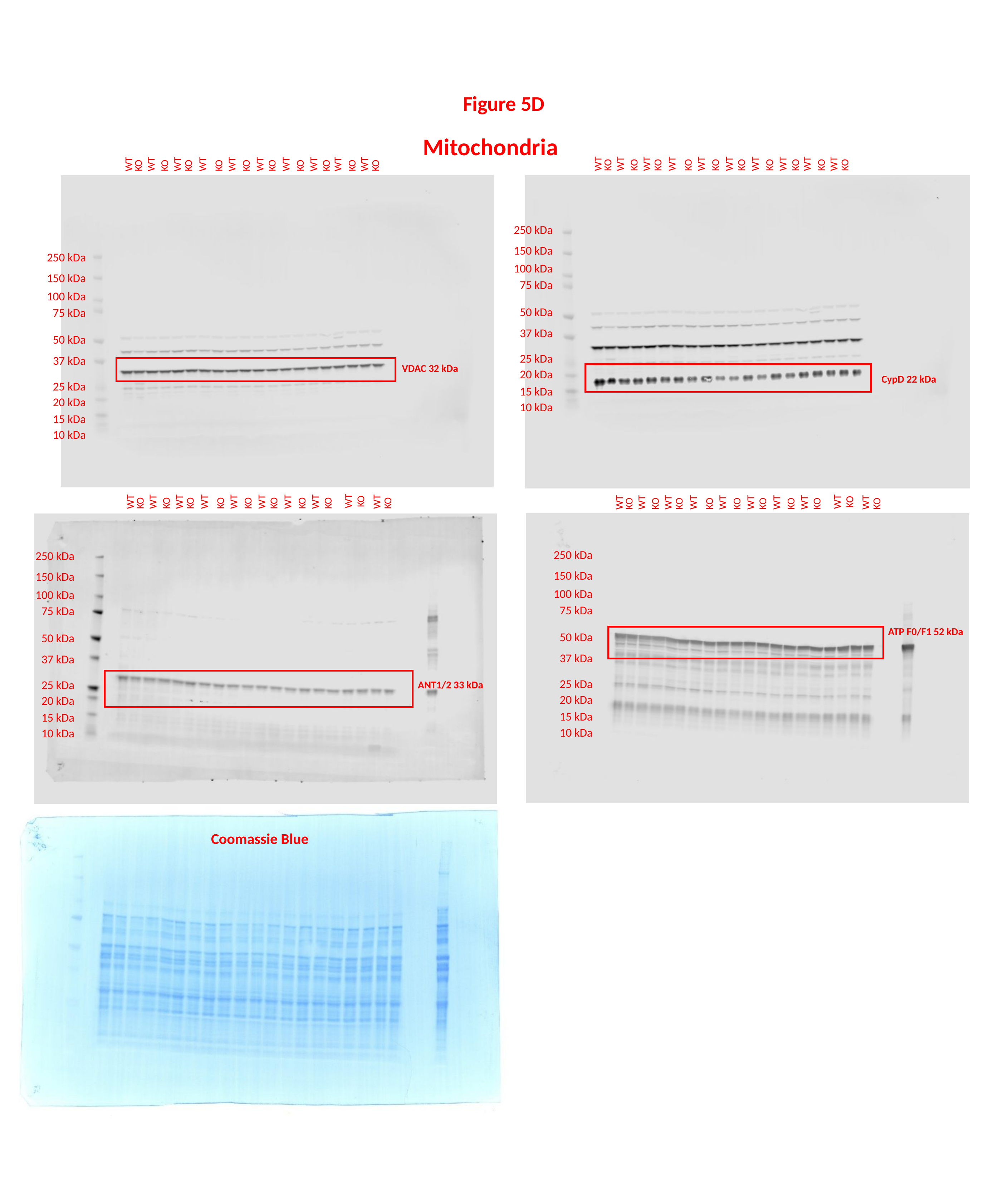

Figure 5D
WT
WT
WT
WT
WT
WT
WT
WT
KO
KO
KO
KO
KO
KO
KO
KO
WT
WT
KO
KO
WT
WT
WT
WT
WT
WT
WT
WT
KO
KO
KO
KO
KO
KO
KO
KO
WT
WT
KO
KO
Mitochondria
250 kDa
150 kDa
100 kDa
75 kDa
50 kDa
37 kDa
25 kDa
20 kDa
15 kDa
10 kDa
250 kDa
150 kDa
100 kDa
75 kDa
50 kDa
37 kDa
25 kDa
20 kDa
15 kDa
10 kDa
VDAC 32 kDa
CypD 22 kDa
WT
WT
WT
WT
WT
WT
WT
WT
KO
KO
KO
KO
KO
KO
KO
KO
WT
WT
KO
KO
WT
WT
WT
WT
WT
WT
WT
WT
KO
KO
KO
KO
KO
KO
KO
KO
WT
WT
KO
KO
250 kDa
150 kDa
100 kDa
75 kDa
50 kDa
37 kDa
25 kDa
20 kDa
15 kDa
10 kDa
250 kDa
150 kDa
100 kDa
75 kDa
50 kDa
37 kDa
25 kDa
20 kDa
15 kDa
10 kDa
ATP F0/F1 52 kDa
ANT1/2 33 kDa
Coomassie Blue

## Slide 15
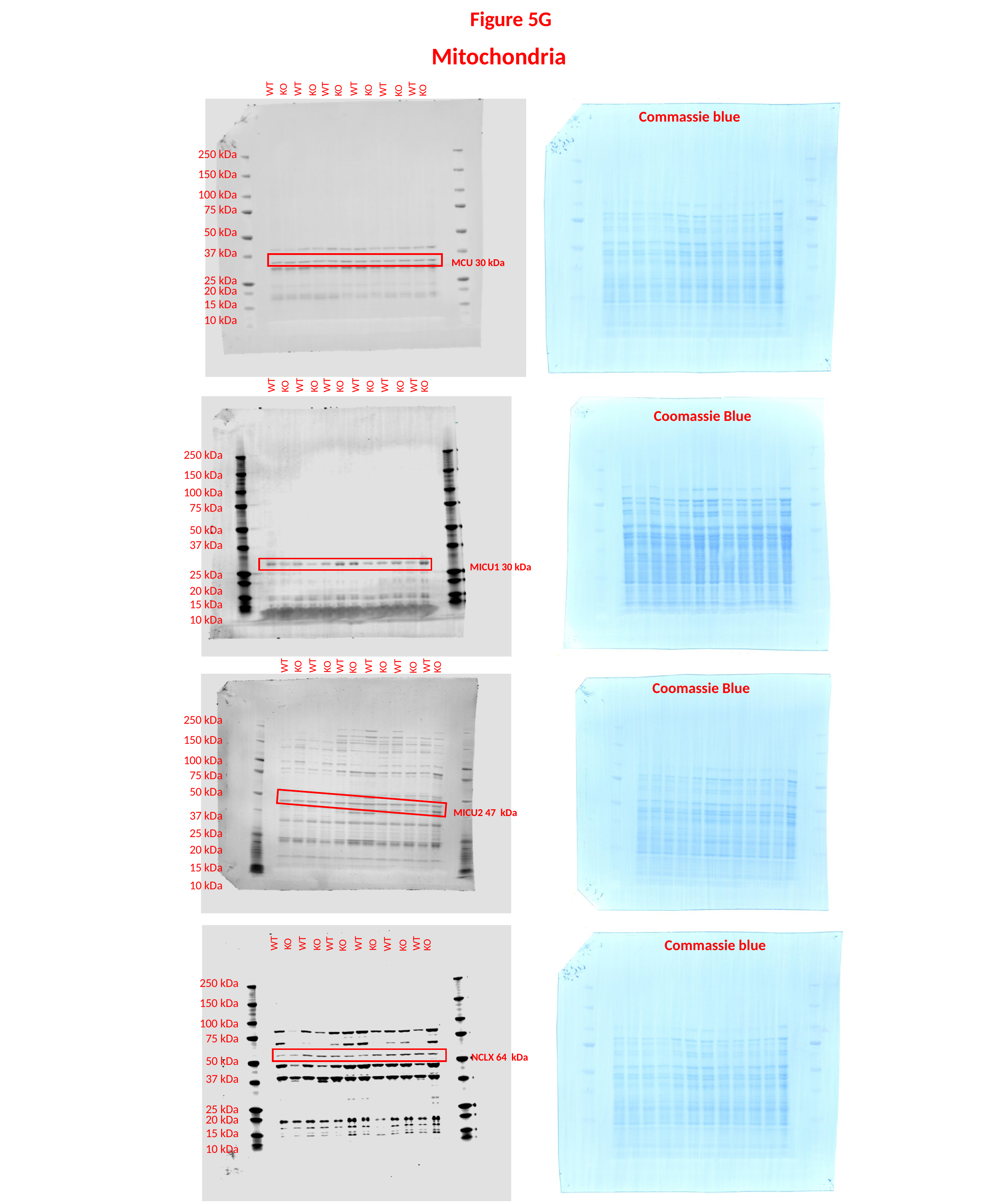

Figure 5G
Mitochondria
WT
WT
WT
WT
WT
WT
KO
KO
KO
KO
KO
KO
Commassie blue
250 kDa
150 kDa
100 kDa
75 kDa
50 kDa
37 kDa
25 kDa
20 kDa
15 kDa
10 kDa
MCU 30 kDa
WT
WT
WT
WT
WT
WT
KO
KO
KO
KO
KO
KO
Coomassie Blue
250 kDa
150 kDa
100 kDa
75 kDa
50 kDa
37 kDa
25 kDa
20 kDa
15 kDa
10 kDa
MICU1 30 kDa
WT
WT
WT
WT
WT
WT
KO
KO
KO
KO
KO
KO
Coomassie Blue
250 kDa
150 kDa
100 kDa
75 kDa
50 kDa
37 kDa
25 kDa
20 kDa
15 kDa
10 kDa
MICU2 47 kDa
WT
WT
WT
WT
WT
WT
KO
KO
KO
KO
KO
KO
Commassie blue
250 kDa
150 kDa
100 kDa
75 kDa
50 kDa
37 kDa
25 kDa
20 kDa
15 kDa
10 kDa
NCLX 64 kDa

## Slide 16
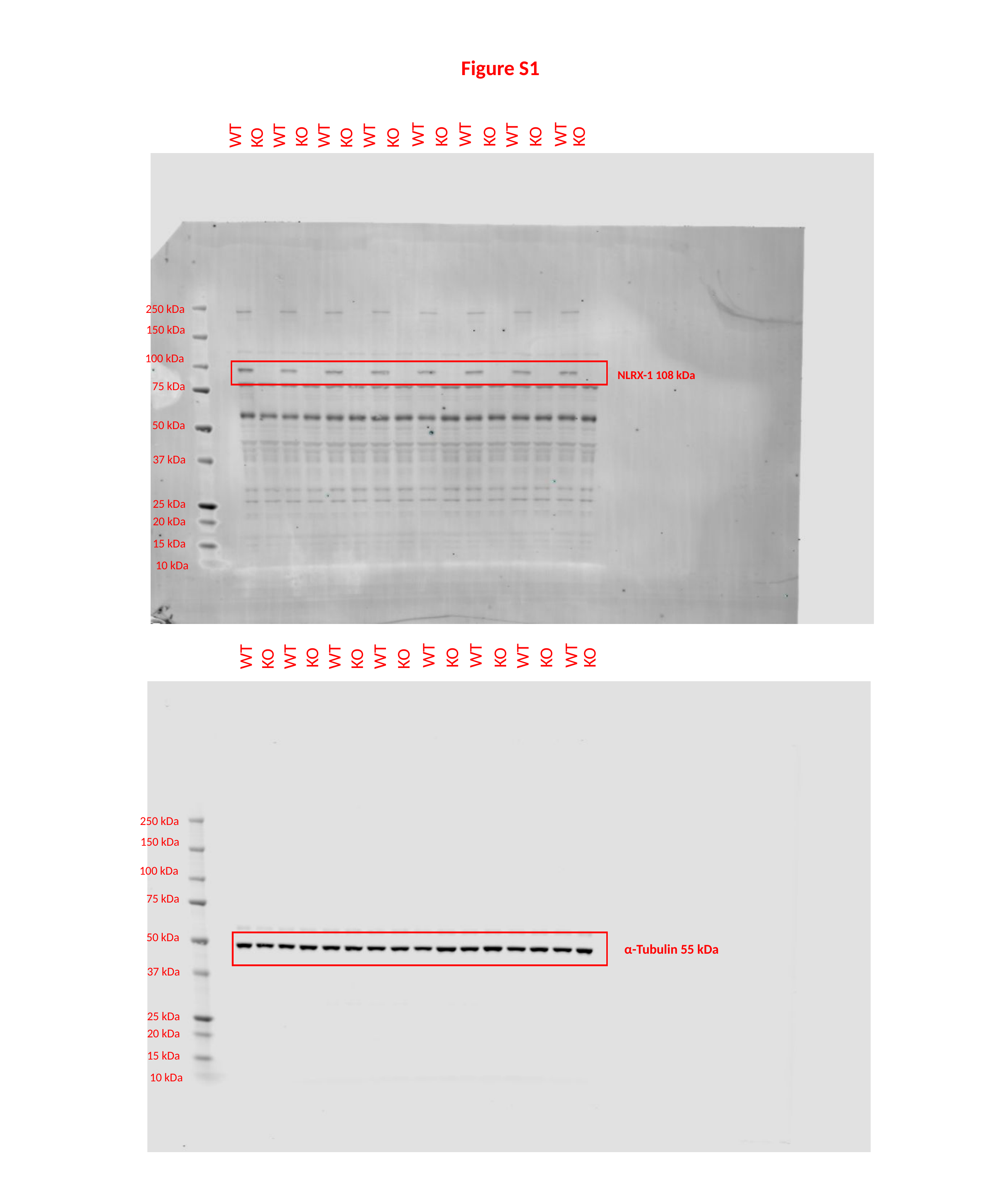

Figure S1
WT
WT
WT
WT
WT
WT
WT
WT
KO
KO
KO
KO
KO
KO
KO
KO
250 kDa
150 kDa
100 kDa
75 kDa
50 kDa
37 kDa
25 kDa
20 kDa
15 kDa
10 kDa
NLRX-1 108 kDa
α-Tubulin
WT
WT
WT
WT
WT
WT
WT
WT
KO
KO
KO
KO
KO
KO
KO
KO
250 kDa
150 kDa
100 kDa
75 kDa
50 kDa
37 kDa
25 kDa
20 kDa
15 kDa
10 kDa
α-Tubulin 55 kDa

## Slide 17
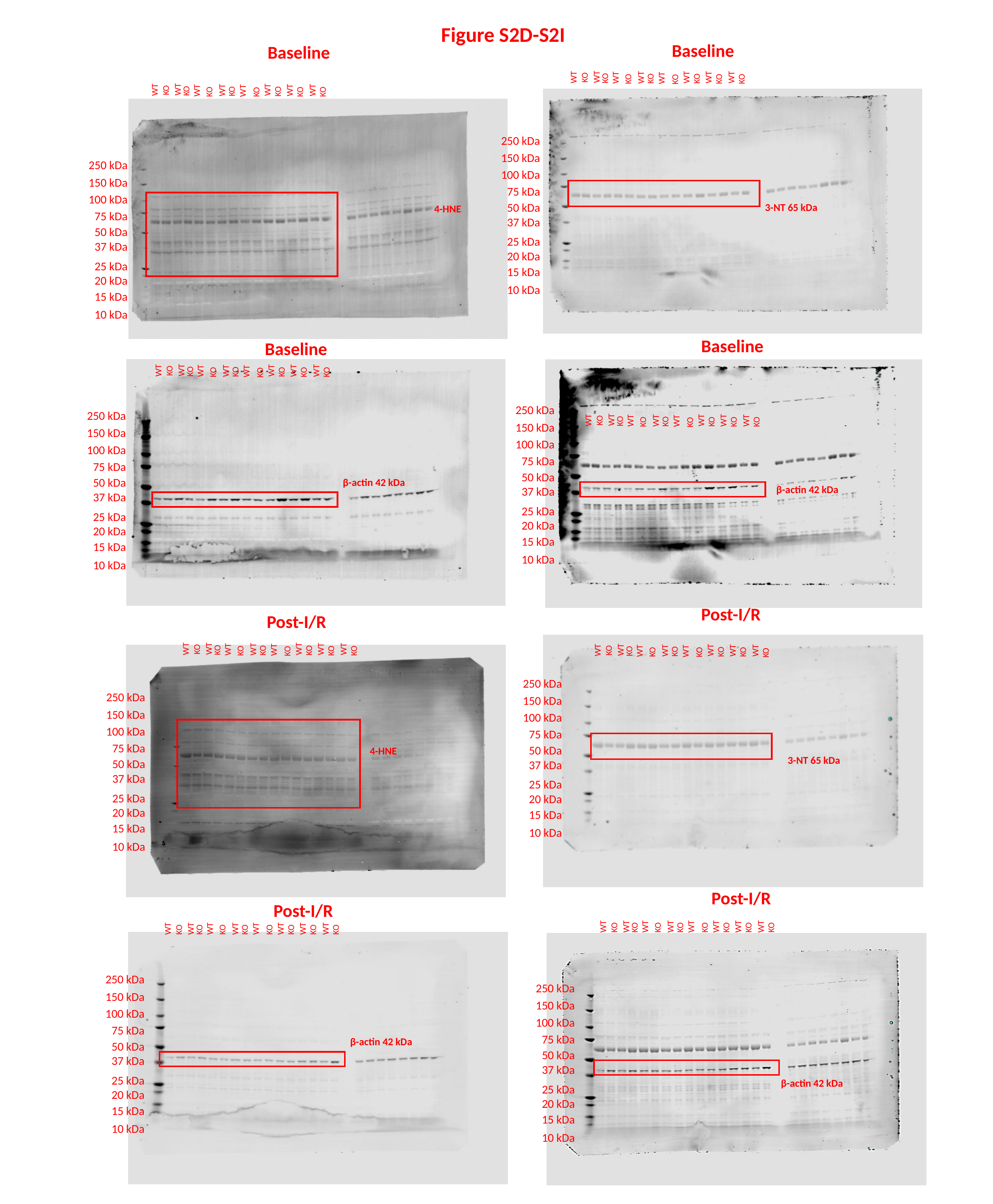

Figure S2D-S2I
WT
WT
WT
WT
WT
WT
KO
KO
KO
KO
KO
KO
WT
WT
KO
KO
Baseline
Baseline
WT
WT
WT
WT
WT
WT
KO
KO
KO
KO
KO
KO
WT
WT
KO
KO
250 kDa
150 kDa
100 kDa
75 kDa
50 kDa
37 kDa
25 kDa
20 kDa
15 kDa
10 kDa
250 kDa
150 kDa
100 kDa
75 kDa
50 kDa
37 kDa
25 kDa
20 kDa
15 kDa
10 kDa
3-NT 65 kDa
4-HNE
WT
WT
WT
WT
WT
WT
KO
KO
KO
KO
KO
KO
WT
WT
KO
KO
Baseline
Baseline
WT
WT
WT
WT
WT
WT
KO
KO
KO
KO
KO
KO
WT
WT
KO
KO
250 kDa
150 kDa
100 kDa
75 kDa
50 kDa
37 kDa
25 kDa
20 kDa
15 kDa
10 kDa
250 kDa
150 kDa
100 kDa
75 kDa
50 kDa
37 kDa
25 kDa
20 kDa
15 kDa
10 kDa
β-actin 42 kDa
β-actin 42 kDa
WT
WT
WT
WT
WT
WT
KO
KO
KO
KO
KO
KO
WT
WT
KO
KO
WT
WT
WT
WT
WT
WT
KO
KO
KO
KO
KO
KO
WT
WT
KO
KO
Post-I/R
Post-I/R
250 kDa
150 kDa
100 kDa
75 kDa
50 kDa
37 kDa
25 kDa
20 kDa
15 kDa
10 kDa
250 kDa
150 kDa
100 kDa
75 kDa
50 kDa
37 kDa
25 kDa
20 kDa
15 kDa
10 kDa
4-HNE
3-NT 65 kDa
WT
WT
WT
WT
WT
WT
WT
WT
KO
KO
KO
KO
KO
KO
KO
KO
WT
WT
WT
WT
WT
WT
WT
WT
KO
KO
KO
KO
KO
KO
KO
KO
Post-I/R
Post-I/R
250 kDa
150 kDa
100 kDa
75 kDa
50 kDa
37 kDa
25 kDa
20 kDa
15 kDa
10 kDa
250 kDa
150 kDa
100 kDa
75 kDa
50 kDa
37 kDa
25 kDa
20 kDa
15 kDa
10 kDa
β-actin 42 kDa
β-actin 42 kDa
